# Determination of genetic correlation between tobacco smoking and coronary artery disease

**DOI:** 10.3389/fpsyt.2023.1279962

**Published:** 2023-09-26

**Authors:** Zhouhai Zhu, Qiang Liu, Meng Li, Yinghao Yao, Feiyan Qi, Yi Xu, Sheming Lu, Zhongli Yang, Ying Guan, Ming D. Li, Jianhua Yao

**Affiliations:** ^1^Joint Institute of Tobacco and Health, Kunming, Yunnan, China; ^2^State Key Laboratory for Diagnosis and Treatment of Infectious Diseases, The First Affiliated Hospital, Collaborative Innovation Center for Diagnosis and Treatment of Infectious Diseases, Zhejiang University School of Medicine, Hangzhou, Zhejiang, China

**Keywords:** smoking, CAD, coronary artery disease, genome-wide association study, mendelian randomization analysis

## Abstract

**Backgrounds:**

Tobacco smoking is an important risk factor for coronary artery disease (CAD), but the genetic mechanisms linking smoking to CAD remain largely unknown.

**Methods:**

We analyzed summary data from the genome-wide association study (GWAS) of the UK Biobank for CAD, plasma lipid concentrations (*n* = 184,305), and smoking (*n* = 337,030) using different biostatistical methods, which included LD score regression and Mendelian randomization (MR).

**Results:**

We identified SNPs shared by CAD and at least one smoking behavior, the genes where these SNPs are located were found to be significantly enriched in the processes related to lipoprotein metabolic, chylomicron-mediated lipid transport, lipid digestion, mobilization, and transport. The MR analysis revealed a positive correlation between smoking cessation and decreased risk for CAD when smoking cessation was considered as exposure (*p* = 0.001), and a negative correlation between the increased risk for CAD and smoking cessation when CAD was considered as exposure (*p* = 2.95E-08). This analysis further indicated that genetic liability for smoking cessation increased the risk of CAD.

**Conclusion:**

These findings inform the concomitant conditions of CAD and smoking and support the idea that genetic liabilities for smoking behaviors are strongly associated with the risk of CAD.

## Introduction

Tobacco smoking is one of the most important public health problems world-wide, accounting for 9% of deaths (1). Several epidemiologic studies have proved that tobacco smoking is a major risk factor for many diseases such as lung cancer and cardiovascular and respiratory diseases (1). Lung cancer is the most prevalent smoking-associated cause of death, followed by ischemic heart disease and chronic airway obstruction. Tobacco smoking and its impact on the respiratory system caused an estimated 8 million deaths per year, with more than 10% of these deaths related to second-hand smoke (2). In the United States, tobacco smoking is associated with 30% of all CAD-related deaths each year (3) and also doubles the risk of premature cardiovascular deaths (4).

Several epidemiologic studies have revealed that tobacco smoking increases the incidence of fatal CAD and associates with various cardiovascular diseases (5–8). Extensive clinical evidence has supported the idea that tobacco smoking causes multiple genetic and epigenetic abnormalities in the respiratory epithelium (9, 10). In a previous study, Sabater-Ileal and colleagues identified a genetic locus that influences both lung function and CAD (11), although the findings were not genome-wide in scale and were underpowered due to a small sample size.

Tobacco contains more than 4,000 chemicals (12), and the exact toxic components and the mechanisms involved in tobacco-related CAD and cardiovascular dysfunction are still unknown. Recently, a genetic predisposition to the development of atherogenesis in individuals exposed to cigarette smoke has been reported. The commonly documented examples are *CYP1A1* MSP polymorphism and certain endothelial NO synthase intron 4 polymorphisms. Both increase the susceptibility to cigarette smoke exposure-related atherosclerotic diseases including multi-vessel CAD and myocardial infarction (MI) (13, 14). Given that much of the available data were derived from observational studies, which are unable to account fully for confounding and reverse causation, the genetic correlation and causal relations between smoking and cardiovascular diseases remain to be determined.

The principle of Mendelian randomization (MR) relies on the basic laws of Mendelian genetics, segregation, and independent association. When these principles hold at a population level, the influence of confounding factors can be reduced because religion, growth, environment, and other confounding factors are considered to be random (15). Given that alleles are randomly allocated and become fixed at conception, MR studies are less susceptible to reverse causality than are observational studies.

In this study, we examined the pleiotropic effect of tobacco smoking and CAD using publicly available GWAS summary statistics. Then, we used bidirectional MR method to reveal the nature of the causal relations between CAD and tobacco smoking. Finally, we determined the biological processes or pathways involved in the comorbidity of these two diseases.

## Materials and methods

### GWAS summary data sets

The GWAS summary data for CAD and plasma lipid concentrations have been described in a previous report (16). Briefly, the summary statistics of a large GWAS meta-analysis comprising more than 120,000 CAD cases and 339,115 controls were obtained from CARDIoGRAMplusC4D Consortium website (http://www.cardiogramplusc4d.org/data-downloads/). A total of 9,149,595 variants were included either in the CARDIoGRAMplusC4D 1,000 Genomes–imputed GWAS or the MIGen/CARDIoGRAM Exome chip study. The smoking data were obtained from the large GSCAN summary statistics (17). The GSCAN investigated four smoking-related phenotypes, including age at initiation of regular smoking (AgeSmk; *n* = 341,427), whether an individual had ever smoked regularly (SmkInit; *n* = 1,232,091), cigarettes smoked per day (CPD; *n* = 337,334), and smoking cessation (SmkCes; *n* = 547,219). The GWAS summary statistics for different smoking phenotypes can be found at https://conservancy.umn.edu/handle/11299/201564.

Further, we obtained published GWAS meta-analysis association data for lipid concentrations from the Center for Statistical Genetics, which was a joint analysis that examined 188,577 individuals whose genomic DNA samples were genotyped with two platforms from multiple studies (18). Complete GWAS summary statistics were downloaded from webpage http://csg.sph.umich.edu/willer/public/lipids2013/.

### Estimation of genetic correlation by LD score regression (LDSC)

The genetic correlations (*r_g_*) between CAD and smoking behaviors were estimated by LDSC (19). Pairwise LD *r^2^* among SNPs was conducted using pre-computed LD scores with the 1,000 Genomes Project reference panel of subjects of European ancestry. Quality control steps were adopted from LD scores default procedures, including imputation quality >0.9 and minor allele frequency > 0.1. Moreover, all SNPs retained for further analysis were merged with SNPs in the HapMap 3 reference panel. Correlation was considered significant at a corrected *p* value of <0.05 by Bonferroni correction.

### Mendelian randomization (MR)

We extracted the effect estimates and standard errors (Ses) from relevant GWAS and employed TwoSampleMR (v. 0.4.22) R package to clarify the potential causal effect for both smoking and CAD (20). The following strategies were used to identify genetic instruments. First, we filtered GWAS summary datasets to require shared susceptible loci in both smoking and CAD. The variants showing genome-wide significance (*p* < 5 × 10^−8^) in GWAS for CAD were considered to be candidate variants, and then we checked the significance of these genetic loci separately in four other smoking behaviors: smoking initiation age, smoking cessation, CPD, and age of initiation in GSCAN studies. The common SNPs were harmonized using default parameters within the built-in “harmonize data” function and then trimmed by PLINK (v. 1.07) to obtain independent risk variants for each disease (21).

To start, the MR analysis was performed by generating instrumental variable estimates for each SNP. The averaged causal estimate of each SNP was calculated using the inverse–variance-weighted (IVW) method, i.e., specifically defined as the beta coefficient associated with SNP-CAD divided by the beta coefficient associated with SNP-smoking behaviors (22). In addition, we used a series of sensitivity analyses, which included weighted median and MR Egger, to evaluate the reliability of our results.

### Gene and pathway analysis

The gene-based analysis that links SNPs to genes was conducted using MAGMA with default settings. To gain biological insights into shared genes, we used the WebGestalt tool (23) to assess enrichment of the identified shared gene set in the Gene Ontology (GO) biological processes with redundant GO terms been removed. Both analyses were based on shared genes that were identified from cross-trait meta-analysis. Pathways with a false discovery rate (FDR) < 0.05 were considered significant.

## Results

### Susceptible loci shared by CAD and smoking behaviors

**Figure 1:**
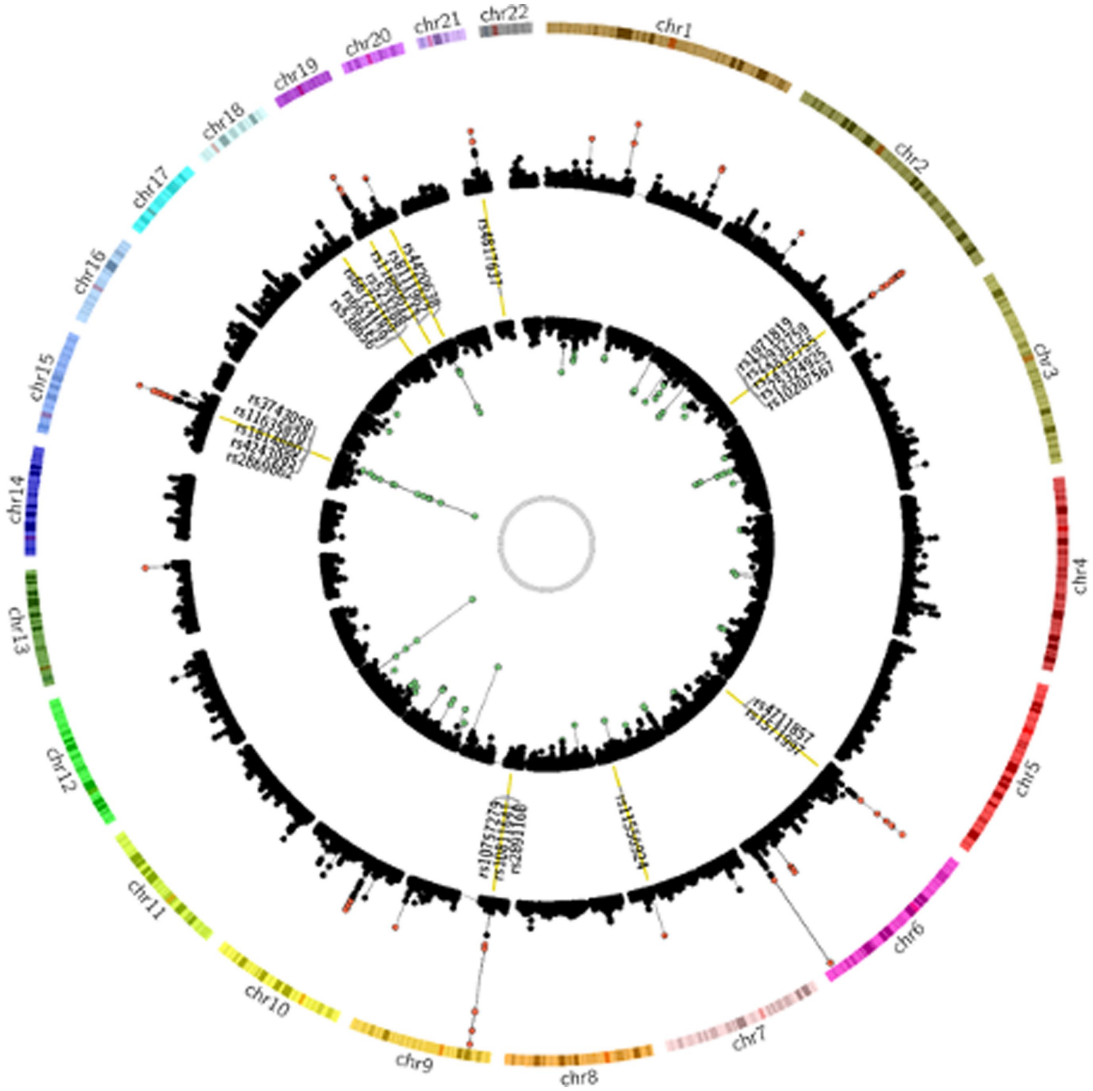
Genetic susceptibility map for CAD and smoking behaviors. Outer ring defines location of 22 human autosomes. Scatter plots in second and fourth rings demonstrate analogy of Manhattan plot for association results from CAD and smoking behaviors, respectively. Altitude of each dot represents statistical significance as −log_10_ (P). SNPs that reached genome-wide significance are colored red for CAD and green for smoking behaviors. Yellow bars in third ring mark 24 CAD risk loci at least nominally associated with smoking behaviors, and tag SNPs in these loci are labeled.

To investigate the genetic overlap between CAD and smoking behaviors, we used GWAS summary data from large-scale genome-wide studies (Supplementary Table S1). We detected a great number of significantly associated SNPs overlapped between CAD risk loci and at least one smoking phenotype (Supplementary Table S2). Of the CAD GWAS loci, 2091 SNPs (35.01%) showed nominal significance in CPD, 526 SNPs (25.16%) showed nominal significance for smoking initiation, 317 SNPs (15.16%) for smoking cession, and 85 SNPs (4.07%) for age at smoking initiation. Notably, 24 SNPs reached genome-wide significance (Figure 1).

### Genetic correlations between CAD and smoking behaviors

**Figure 2:**
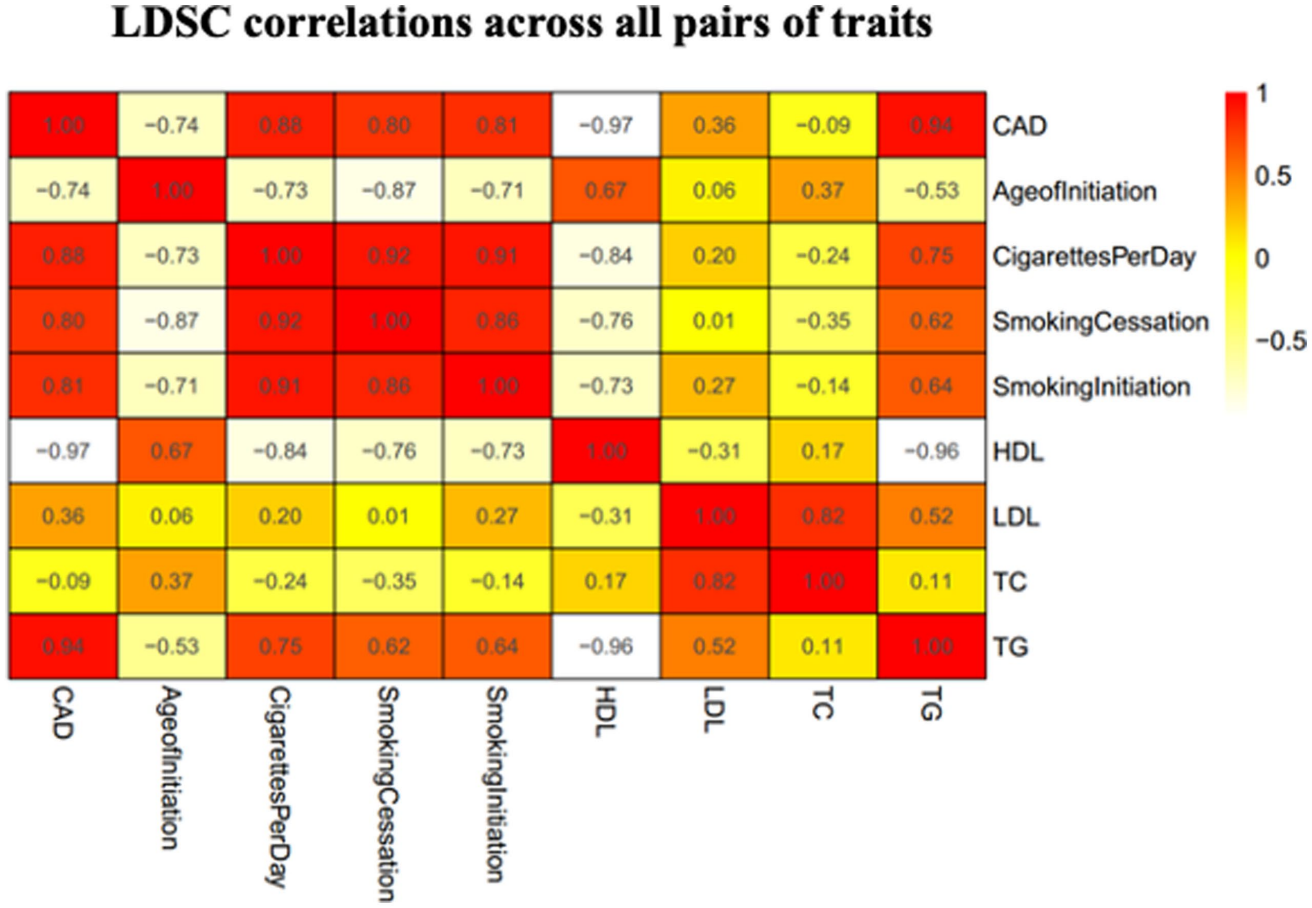
Heatmap showing patterns of genetic correlations across CAD and smoking. Red color indicates a positive correlation and yellow color indicates a negative correlation. CAD, coronary artery disease; HDL, high-density lipoprotein; AgeofInitiation, age of Initiation; CigarettesPerDay, cigarettes per day; SmokingCessation, smoking cessation; SmokingInitiation, smoking Initiation; LDL, low-density lipoprotein; TG, triglycerides; TC, total cholesterol.

We used the LDSC method to test for the genome-wide correlations between CAD and smoking behaviors. Significant genetic correlations were found between CAD and all the smoking-related traits with the smallest *p* values <1 × 10^−18^ (Figure 2). We observed significant positive genetic correlations between CAD and CPD, smoking initiation, and smoking cessation (r_g_ > 0.2), but a negative genetic correlation between CAD and age at initiation (r_g_ < −0.2).

### Mendelian randomization analysis

Considering the presence of potential LD relations among those significant SNPs of interest, we performed *p* value-informed LD pruning with the goal of obtaining independent GWAS SNPs. This led to the identification of 15, 63, 18, and 152 independent SNPs for Age of Initiation, CPD, Smoking Cessation and Smoking Initiation, respectively. Bi-directional MR analysis provided strong evidence that smoking initiation increased the risk of CAD (IVW: *β* = 0.191; *p* = 2.59 × 10^−6^) with a consistent direction of effect in all three MR methods (Table 1). There also was evidence for a consistent but weaker genetic liability for smoking cessation on CAD (IVW: *β* = 0.234; *p* = 0.001). The same findings were observed for age at initiation as the instrument on CAD (IVW: *β* = 0.295; *p* = 0.039).

**Table 1:** Effect of smoking behaviors on CAD using two-sample MR analysis.

Exposure	Outcome	No. SNPs	Beta	*p*-value	Method
Age of initiation	CAD	15	0.295	0.039	Inverse-variance weighted
		15	0.295	0.026	Weighted median
		15	−0.443	0.609	MR Egger
CPD	CAD	63	−0.039	0.406	Inverse-variance weighted
		63	−0.072	0.097	Weighted median
		63	−0.175	0.025	MR Egger
Smoking cessation	CAD	18	0.234	0.001	Inverse-variance weighted
		18	0.234	0.001	Weighted median
		18	0.091	0.559	MR Egger
Smoking initiation	CAD	152	0.191	2.59E-06	Inverse-variance weighted
		152	0.193	3.35E-06	Weighted median
		152	0.419	0.052	MR Egger

On the other hand, we obtained negative correlations between CPD and CAD, although the statistic was less significant. Only the result of MR Egger was significant. When treating CAD as an instrument, strong evidence of decreased risk of smoking cessation by CAD was observed (IVW: *β* = −0.046; *p* = 2.95 × 10^−8^; Table 2). The effects of CAD on other smoking behaviors became even weaker and inconsistent across the different methods.

**Table 2:** Effect of CAD on smoking behaviors using two-sample MR analysis.

Exposure	Outcome	No. SNPs	Beta	*p*-value	Method
	Age of initiation	68	0.012	0.013	Inverse-variance weighted
CAD		68	0.012	0.03	Weighted median
		68	−0.001	0.932	MR Egger
		68	−0.047	0.075	Inverse-variance weighted
CAD	CPD	68	−0.063	0.018	Weighted median
		68	−0.055	0.366	MR Egger
	Smoking cessation	75	−0.046	2.95E-08	Inverse-variance weighted
CAD		75	−0.046	4.29E-06	Weighted median
		75	−0.079	6.10E-04	MR Egger
	Smoking initiation	68	0.004	0.584	Inverse-variance weighted
CAD		68	0.003	0.637	Weighted median
		68	−0.019	0.299	MR Egger

### Biological pathway and enrichment analysis

We performed pathway analyses to identify biological pathways enriched for shared genetic loci related to smoking and CAD based on significant cross-trait meta-analysis results. For a detailed list of the overlapped genes and SNPs between CAD and smoking-related phenotypes, please refer to Table 3. Pathway analysis showed that the SNP-related genes were significantly enriched in lipoprotein metabolic, chylomicron-mediated lipid transport, lipid digestion, mobilization, and transport (Table 4). The GO analysis suggested that shared genes in CAD and smoking behaviors were enriched in triglyceride-rich lipoprotein particle clearance, blood vessel development, and very-low-density lipoprotein particle clearance (Table 5).

**Table 3:** The overlapped genes and SNPs between CAD and smoking-related phenotypes.

Gene	Gene function	SNPs
ABI2	ABL Interactor 2	rs116426890
ADAMTS7	ADAM metallopeptidase with Thrombospondin type 1 motif 7	rs7182642; rs4887096; rs55834964; rs7174367; rs12916326; rs12916648; rs1809419; rs1809409; rs11635870; rs11635931; rs1807006; rs12286; rs1807007; rs8043119; rs11633351; rs6495267; rs1809420; rs4886591; rs2904223; rs4887102; rs35934157
ALDH2	Aldehyde dehydrogenase 2 family member	rs11513729
APOB	Apolipoprotein B	rs503105
APOC1	Apolipoprotein C1	rs12721051; rs56131196; rs4420638; rs12721046; rs111789331; rs66626994; rs10414043; rs7256200
APOC1P1	Apolipoprotein C1 pseudogene 1	rs111789331; rs66626994
APOE	Apolipoprotein E	rs429358; rs10414043; rs7256200; rs769449
ATXN2	Ataxin 2	rs4766578;rs10774625;rs653178;rs597808;rs7137828;rs11065979; rs11065987
BRAP	BRCA1 associated protein	rs11065979; rs11065987; rs11065991
C19orf38	Chromosome 19 open reading frame 38	rs11881156; rs12973042; rs12979495; rs67987899; rs11881438; rs7260254; rs35443547
CARF	Calcium responsive transcription factor	rs140244541; rs72928613; rs72936866; rs72932559; rs114702158; rs72928620; rs72932566; rs72928610; rs114123510; rs72928609; rs72932560; rs72926798; rs141002954; rs72932554;rs72932561; rs9749722; rs74421437; rs72936872; rs72936873
CARM1	Coactivator associated arginine methyltransferase 1	rs12971616; rs4804547; rs17616105; rs12976693; rs35991287; rs74179956; rs12977506; rs73009538; rs4804142; rs11880628; rs35734575; rs11879571; rs36045835; rs7256879; rs34857893; rs2053064; rs1971038; rs8105092; rs2053065; rs1541595; rs1971039; rs2304089
CDKN2B-AS1	CDKN2B antisense RNA 1	rs568447; rs545226; rs1333045; rs10217586; rs10738604; rs10811641; rs3217992; rs3218020; rs2891168; rs4977574; rs10757274; rs504318; rs10757264; rs7028268; rs567453; rs496892; rs490005; rs10757272; rs10738606; rs10120688; rs10738607; rs10811645; rs10811643; rs10738610; rs7035484; rs7049105; rs1591136; rs10811647; rs3218012; rs10115049; rs10811656; rs10757266; rs10757279; rs10811650
CELSR2	Cadherin EGF LAG seven-pass G-type receptor 2	rs11102967; rs611917
CHRNB4	Cholinergic receptor nicotinic beta 4 subunit	rs56354501; rs8031513; rs56195905; rs1809412; rs1814880; rs11072799; rs1809414; rs11856441; rs12901228; rs2219939; rs1825086; rs1383635; rs1383634; rs58717592; rs59671175; rs1809415
CNNM2	Cyclin and CBS domain divalent metal cation transport mediator 2	rs12414777
COL4A1	Collagen type IV alpha 1 chain	rs12866570
COL4A2	Collagen type IV alpha 2 chain	rs55940034; rs9515201
CYP20A1	Cytochrome P450 family 20 subfamily A member 1	rs72938351; rs72938315; rs115600411; rs114863726; rs114407963; rs116443099
DMRTA1	DMRT like family A1	rs10738610; rs10811656; rs10757279; rs10757277; rs1333046; rs1333049; rs10757278; rs1333048; rs4977575; rs7857118; rs1333047
DNM2	Dynamin 2	rs117159625; rs11880613; rs117786851; rs8111962; rs1109375; rs2287029
EDNRA	Endothelin receptor type A	rs7668383
FAM117B	Family with sequence similarity 117 member B	rs146289328
FES	FES proto-oncogene, tyrosine kinase	rs2521501
GGCX	Gamma-glutamyl carboxylase	rs7568458; rs35215812; rs10198569; rs10187424; rs12473819; rs6547624; rs6757263
HECTD4	HECT domain E3 ubiquitin protein ligase 4	rs11066188; rs7953257
ICA1L	Islet cell autoantigen 1 like	rs11675462; rs4675290; rs2036927; rs12693975; rs10207567; rs1971819; rs934287; rs72932720; rs72932716; rs72932711; rs72932709; rs72932707; rs76122535; rs72932722; rs72932723; rs72932731; rs72932737; rs72934734; rs72932725; rs143911965; rs6435168; rs6732078; rs72932746; rs80087860; rs6705330; rs72932752; rs78128841
IL6R	Interleukin 6 receptor	rs6694258; rs6689393; rs4845625; rs12129500; rs6686750; rs4845619; rs7549338; rs6667434; rs7553796; rs4553185; rs59632925; rs7549250; rs4845371; rs11265612; rs6687726; rs4845618; rs6694817; rs11265611; rs6427658; rs12118721; rs10908838; rs12118770; rs12117832; rs6689306
KCNE2	Potassium voltage-gated channel subfamily E regulatory subunit 2	rs1018757; rs2211693; rs2211695; rs2211694; rs8128536; rs7279974; rs8129119; rs4817636; rs2096469; rs4817637; rs2096467; rs2834439; rs7280276; rs9982672; rs1023354; rs28451064; rs762158; rs9980618; rs743339; rs7277800; rs9982601; rs4817639; rs9974878; rs7281592; rs60687229; rs2834431; rs28591415; rs16991453; rs9978142; rs7283231; rs8132042; rs8131284
KCNK5	Potassium two pore domain channel subfamily K member 5	rs1155347; rs10456468; rs56015508; rs55902013; rs9394577; rs12211281; rs55856036; rs1544935
LDLR	Low density lipoprotein receptor	rs8103309; rs11666925; rs876794; rs10409001; rs10415811; rs11085757; rs8102273; rs11879026; rs73013202; rs73013198
LINC00310	Long intergenic non-protein coding RNA 310	rs1018757; rs2211693; rs2211695; rs2211694; rs8128536; rs7279974; rs8129119; rs4817636; rs2096469; rs4817637; rs2096467; rs2834439; rs7280276; rs9982672; rs1023354; rs28451064
LINC00841	Long intergenic non-protein coding RNA 841	rs2051120; rs6593388; rs2624688; rs6593393; rs4948800; rs2085797; rs7478408; rs898549; rs10899956; rs12359058; rs10899955; rs7924201; rs7088951; rs10899963; rs10899954; rs898551; rs10793515; rs66887775
LOC646938	TBC1 domain family member 2B pseudogene	rs56354501; rs8031513; rs55834964; rs56195905; rs1809412; rs1814880; rs11072799; rs1809414; rs11856441; rs12901228; rs2219939; rs1825086
MAP3K4	Mitogen-activated protein kinase kinase kinase 4	rs117340856
MAPKAPK5-AS1	MAPKAPK5 antisense RNA 1	rs11513729
MC4R	Melanocortin 4 receptor	rs523288; rs538656; rs571312; rs663129; rs6567160; rs34633411; rs11152213; rs66723169; rs1942872; rs12958167; rs12955983; rs12954782; rs11663816
MIA3	MIA SH3 domain ER export factor 3	rs1909196; rs34679168; rs34767248; rs2378584; rs17163301; rs4618978; rs4846769; rs4846384; rs4846770; rs17163345; rs17163313
MIR4422	Microrna 4,422	rs55694910; rs72664304; rs72664303
MORF4L1	Mortality factor 4 like 1	rs11634042; rs8034274; rs7168915; rs11852830; rs11857877; rs4439728; rs12232282; rs4420501; rs4438276; rs8037171; rs11637783
MRAS	Muscle RAS oncogene homolog	rs13324341; rs1199337; rs1199338; rs185244
MTAP	Methylthioadenosine phosphorylase	rs7041637
NAA25	N-alpha-acetyltransferase 25, NatB auxiliary subunit	rs17696736
NBEAL1	Neurobeachin like 1	rs72932566; rs76461893; rs145299755; rs72932573; rs148707292; rs72932572; rs72932574; rs72932583; rs6728861; rs72932575; rs4675310; rs148812085; rs2351524; rs140750546; rs72934573; rs140168762; rs72934505; rs145538381; rs115654617
NOS3	Nitric oxide synthase 3	rs3918226
PHACTR1	Phosphatase and actin regulator 1	rs10807323; rs13209002; rs9381401; rs12202891; rs9395224; rs9472790; rs1571997; rs9381462; rs6916397; rs2026458; rs6916421; rs7776079
PLG	Plasminogen	rs117340856
PMAIP1	Phorbol-12-myristate-13-acetate-induced protein 1	rs523288; rs538656; rs571312; rs663129; rs6567160; rs34633411; rs11152213; rs66723169; rs1942872; rs12958167; rs12955983; rs12954782; rs11663816; rs11664883; rs2045438; rs35476226; rs17175602; rs2045439; rs11660069; rs12957325; rs12970134
RAPH1	RAS association (RalGDS/AF-6) and pleckstrin homology domains 1	rs139644567; rs140274075
SAYSD1	SAYSVFN motif domain containing 1	rs1155347; rs10456468; rs56015508; rs55902013; rs9394577; rs12211281; rs55856036; rs1544935
SH2B3	SH2B adaptor protein 3	rs7310615; rs3184504; rs10774624
SH2D6	SH2 domain containing 6	rs6750847; rs7591175; rs6722691; rs11895399; rs11895401; rs1446669; rs2166529; rs6547620; rs6721924; rs6739015; rs2044474; rs13394343; rs6719046; rs6733913; rs17026396
SMARCA4	SWI/SNF related, matrix associated, actin dependent regulator of chromatin, subfamily a, member 4	rs8103309; rs60314748; rs60448955; rs73013159; rs7275; rs55677033; rs3786727; rs11666925; rs11670205; rs3786725; rs10417578; rs68010235; rs6511718; rs876794
SPECC1L-ADORA2A	SPECC1L-ADORA2A readthrough (NMD candidate)	rs5760347; rs62233136; rs5760359; rs5751841; rs5760368; rs62233133; rs5760350; rs2298379
SVIL	Supervillin	rs1774241; rs1774240; rs1832864; rs12259037; rs12779954; rs1418276; rs4749520; rs11007851; rs10826749
TAF1A-AS1	TAF1A antisense RNA 1	rs1909196; rs34679168; rs4618978
TMED1	Transmembrane p24 trafficking protein 1	rs73007593; rs11881156
TRAFD1	TRAF-type zinc finger domain containing 1	rs17630235
TTC29	Tetratricopeptide repeat domain 29	rs7668383
VAMP8	Vesicle associated membrane protein 8	rs35215812; rs10198569; rs10187424; rs6547624; rs6757263
WDR12	WD repeat domain 12	rs72934734; rs35212307; rs114395475; rs7582720; rs72936856; rs72934751; rs72934763; rs72934749; rs6738618; rs150788469; rs6725887; rs6435169; rs7560547; rs77931721; rs142603618; rs72936846; rs72936852; rs72936830; rs143035655; rs77268589
YIPF2	Yip1 domain family member 2	rs17850995
ZC3HC1	Zinc finger C3HC-type Containing 1	rs11556924; rs56179563

**Table 4:** Detected shared pathway between smoking and CAD based on pathway analysis.

Pathway ID	Pathway name	*p*-value	*q*-value (FDR B&H)	Genes included
PW:0000482	Lipoprotein metabolic	5.15E-06	9.66E-04	*APOB, APOC1, APOE*
1,270,005	Lipoprotein metabolism	4.57E-05	3.86E-03	*APOB, APOC1, APOE, LDLR*
1,270,006	Chylomicron-mediated lipid transport	5.74E-05	4.22E-03	*APOB, APOE, LDLR*
1,270,002	Lipid digestion, mobilization, and transport	2.54E-04	1.18E-02	*APOB, APOC1, APOE, LDLR*
M12950	Angiotensin-converting enzyme 2 regulates heart function	5.49E-04	1.62E-02	*COL4A1, COL4A2*
1,270,115	Metabolism of nitric oxide	1.60E-03	3.41E-02	*NOS3, DNM2*
1,427,851	VLDL interactions	1.75E-03	3.41E-02	*APOB, APOC1*
172,847	Protein digestion and absorption	1.83E-03	3.41E-02	*KCNK5, COL4A1, COL4A2*
1,270,008	LDL-mediated lipid transport	1.91E-03	3.41E-02	*APOB, LDLR*

**Table 5:** Detected shared pathways between CAD and smoking based on GO analysis.

Gene Ontology (GO) ID	Gene Ontology (GO) term	*p*-value	*q*-value (FDR B&H)	Genes included
GO:0071830	Triglyceride-rich lipoprotein particle clearance	5.75E-09	7.17E-06	*APOB, APOC1, APOE, LDLR*
GO:0001568	Blood vessel development	1.94E-06	1.34E-03	*APOB, APOE, NOS3, PLG, COL4A1, COL4A2, DNM2, EDNRA, MIA3, SMARCA4, IL6R, LDLR*
GO:0034447	Very-low-density lipoprotein particle clearance	2.16E-06	1.34E-03	*APOB, APOC1, APOE*
GO:0001944	Vasculature development	2.95E-06	1.43E-03	*APOB, APOE, NOS3, PLG, COL4A1, COL4A2, DNM2, EDNRA, MIA3, SMARCA4, IL6R, LDLR*
GO:0072358	Cardiovascular system development	3.43E-06	1.43E-03	*APOB, APOE, NOS3, PLG, COL4A1, COL4A2, DNM2, EDNRA, MIA3, SMARCA4, IL6R, LDLR*
GO:0042159	Lipoprotein catabolic process	8.10E-06	2.53E-03	*APOB, APOE, LDLR*
GO:0030195	Negative regulation of blood coagulation	1.53E-05	3.72E-03	*APOE, NOS3, PLG, SH2B3*
GO:0048514	Blood vessel morphogenesis	2.76E-05	4.31E-03	*APOB, APOE, NOS3, PLG, COL4A1, COL4A2, EDNRA, MIA3, SMARCA4, LDLR*
GO:1990777	Lipoprotein particle	2.51E-06	2.30E-04	*APOB, APOC1, APOE, LDLR*
GO:0034358	Plasma lipoprotein particle	2.51E-06	2.30E-04	*APOB, APOC1, APOE, LDLR*
GO:0032994	Protein-lipid complex	3.09E-06	2.30E-04	*APOB, APOC1, APOE, LDLR*
GO:0034362	Low-density lipoprotein particle	6.77E-06	3.00E-04	*APOB, APOE, LDLR*
GO:0034364	High-density lipoprotein particle	4.26E-05	1.13E-03	*APOB, APOC1, APOE*

## Discussion

In this study, we revealed the genetic correlation and causal relations between smoking and CAD, providing a comprehensive evaluation of the shared genetic etiology of tobacco smoking and cardiovascular diseases. Our findings have highlighted the discovery that different smoking behaviors have strong associations with CAD, specifically, the correlation between smoking initiation, smoking cessation, and CAD.

The approach to MR is based on the assumptions that: (1) the genetic marker is associated with the exposure; (2) the genetic marker is independent of any confounding factors; and (3) there is no association between the genetic marker and outcome except through confounding factors. However, it should be acknowledged that these assumptions generally are not easy to evaluate. Results from the present MR study were based on data from the GWAS, which has corroborated the results obtained from conventional prospective observational studies that confirmed that tobacco smoking is a risk factor for CAD (5–8).

To our knowledge, this study represents one of a few large-scale genome-wide analysis to investigate the genetic overlap between smoking and CAD (24–26). Similar to the findings from these reports, our analyses also revealed strong associations between smoking initiation, smoking cessation, and CAD. Further, we found a significant positive association between smoking initiation and CAD when smoking initiation was considered as exposure (inverse–variance-weighted: *β* = 0.191; *p* = 2.59E-06; weighted median: *β* = 0.193; *p* = 3.35E-06), suggesting that smokers are more susceptible to CAD. In addition, we found a negative correlation between CAD and smoking cessation when CAD was considered as exposure (inverse–variance-weighted: *β* = −0.046; *p* = 2.95E-08; weighted median: *β* = 0.193; p = 3.35E-06). This indicates that patients with CAD are less likely to quit smoking, possibly because of tobacco addiction. Together, these findings demonstrate the presence of shared genetic etiologies between tobacco smoking and CAD.

Moreover, we found strong evidence of a genetic correlation between CAD and serum high-density lipoprotein (HDL) and low-density lipoprotein (LDL) as well, which is consistent with the results reported previously (27–29). We observed a positive correlation between LDL and CAD when LDL was considered as the exposure (inverse–variance-weighted: *β* = 0.387; *p* = 4.96E-43; weighted median: *β* = 0.406; *p* = 7.41E-39), and a negative correlation between HDL and CAD when HDL was considered as the exposure (inverse–variance-weighted: *β* = −0.245; *p* = 1.19E-14; weighted median: *β* = −0.188; *p* = 2.66E-10). As is well documented in the literature, LDL is a strong risk factor for CAD (27–29) whereas HDL is an anti-atherosclerotic plasma lipoprotein and a protective factor against CAD (30–32).

Further, we found that smoking has significant associations with HDL and LDL. A positive correlation between CPD and LDL was observed when CPD was considered as exposure (inverse–variance-weighted: β =0.06; *p* = 0.01; weighted median: *β* = 0.06; *p* = 0.02), and a negative correlation between CPD and HDL when CPD was considered as exposure (inverse–variance-weighted: *β* = −0.06; *p* = 0.005; weighted median: *β* = −0.06; p = 0.02). There also is a positive correlation between LDL and smoking cessation when LDL was considered as exposure (inverse–variance-weighted: *β* = −0.03; *p* = 0.002; weighted median: *β* = −0.04; *p* = 2.43E-5). Taken together, these findings provided a clear indication that smoking increases the risk of CAD by affecting the regulation of LDL and HDL, which needs to be further investigated.

We also performed GO enrichment and KEGG pathway analyses based on the genes where the SNPs overlapped between CAD and smoking-related phenotypes are located. We found several functions and pathways to be related to the lipoprotein metabolic and blood vessel development, which are all closely associated with CAD. It has been reported that the APOE–APOC1–APOC2–APOC4 cluster was significantly related to lipoprotein-associated phospholipase A2 mass and activity and CAD (33). Interestingly, the SNP rs4420638 located downstream of the APOC1 gene was found to be significantly related to smoking cessation (*p* = 7.4E-6) (34). Moreover, as a brain eQTL based on the information from BRAINEAC, rs4420638, this SNP has been linked to Alzheimer’s disease (35–40) and cognitive decline (41).

We used non-overlapping data sources in the context of summary-level MR analysis of exposure and outcome, which greatly improved the confidence in the causal effect estimates. In addition, through a range of sensitivity analysis methods, similar causal estimates and consistent causal inferences could be drawn. However, this study had limitations as well. First, there was a stark difference in sample sizes among different phenotypes, which might contribute to discrepancies in statistical power. Second, the information available on the summary-level GWAS data had limited us to divide samples into subgroups, which prevented us from studying the age-related heterogeneity.

In conclusion, this was a systematic analysis of the shared etiology and possible causal relations of smoking and CAD by employing large-scale GWASs. Genetic methods represent another option for assessing causality when there are too many confounding factors in randomized controlled traits, and our findings strongly support the hypothesis that smoking behavior is causally related to CAD risk. We found significant genetic overlap and correlations between CAD and smoking at the SNP level. Taken together, the data from this study enhance the understanding of the genetic etiology of the relations between CAD and smoking and might help to dissect smoking behaviors and develop preventive strategies to reduce the burden of cardiovascular disease in public health.

## Data availability statement

The original contributions presented in the study are included in the article/Supplementary material, further inquiries can be directed to the corresponding authors.

## Author contributions

ZZ: Writing – original draft, Writing – review & editing, Data curation, Formal analysis. QL: Data curation, Formal analysis, Writing – original draft, Methodology. MeL: Data curation, Formal analysis, Methodology, Writing – review & editing. YY: Data curation, Formal analysis, Methodology, Software, Writing – original draft. FQ: Data curation, Formal analysis, Software, Writing – original draft. YX: Data curation, Formal analysis, Methodology, Software, Writing – original draft. SL: Data curation, Formal analysis, Methodology, Writing – review & editing. ZY: Data curation, Project administration, Writing – review & editing. YG: Project administration, Writing – review & editing, Investigation, Resources, Supervision. MiL: Funding acquisition, Investigation, Resources, Writing – review & editing, Conceptualization, Supervision, Writing – original draft. JY: Funding acquisition, Investigation, Project administration, Resources, Writing – review & editing.

## Funding

The author(s) declare financial support was received for the research, authorship, and/or publication of this article. This study was supported by the Joint Institute of Tobacco and Health Open Project Fund, the China Precision Medicine Initiative (2016YFC0906300), Research Center for Air Pollution and Health of Zhejiang University, and the State Key Laboratory for Diagnosis and Treatment of Infectious Diseases of the First Affiliated Hospital of Zhejiang University.

## Conflict of interest

The authors declare that the research was conducted in the absence of any commercial or financial relationships that could be construed as a potential conflict of interest.

The author(s) declared that they were an editorial board member of Frontiers, at the time of submission. This had no impact on the peer review process and the final decision.

## Publisher’s note

All claims expressed in this article are solely those of the authors and do not necessarily represent those of their affiliated organizations, or those of the publisher, the editors and the reviewers. Any product that may be evaluated in this article, or claim that may be made by its manufacturer, is not guaranteed or endorsed by the publisher.
