# Supplementary material for: Determination of genetic correlation between tobacco smoking and coronary artery disease
Source: Front Psychiatry. 2023 Sep 26;14:1279962. doi: 10.3389/fpsyt.2023.1279962 (PMC10562694; doi:10.3389/fpsyt.2023.1279962)
Supplement: Supplementary file 1 [file Table_1.docx]

Supplementary Table 1. GWAS summary association statistics used in current study

| Trait | Sample Size | Reference | URL |
| --- | --- | --- | --- |
| Coronary artery disease | 322,183 | Nelson et al. 2017 | http://www.cardiogramplusc4d.org/data-downloads/ |
| AgeSmk | 341,427 | Liu et al. 2019 |  |
| SmkInit | 1,232,091 | Liu et al. 2019 |  |
| CigDay | 337,334 | Liu et al. 2019 |  |
| SmkCes | 547,219 | Liu et al. 2019 |  |
| LDL Cholesterol | 188,577 | Willer et al. 2013 | http://csg.sph.umich.edu//abecasis/public/lipids2013/ |
| HDL Cholesterol | 188,577 | Willer et al. 2013 | http://csg.sph.umich.edu//abecasis/public/lipids2013/ |
| Triglycerides | 188,577 | Willer et al. 2013 | http://csg.sph.umich.edu//abecasis/public/lipids2013/ |
| Total cholesterol TC | 188,577 | Willer et al. 2013 | http://csg.sph.umich.edu//abecasis/public/lipids2013/ |

Note: AgeSmk: age of initiation of regular smoking; SmkInit: ever smoked regularly; CigDay: cigarettes per day; SmkCes: Smoking cessation

Supplementary Table 2. Significantly associated SNPs overlapped between CAD and at least one smoking phenotype

| SNP ID | P-cad | P-CPD | | P-SmkInt | | P-SmkCes | | P-AgeSmk | |
| --- | --- | --- | --- | --- | --- | --- | --- | --- | --- |
| rs12748266 | 9.74E-08 | 0.235 | 0.539 | | 0.218 | | 0.207 | |  |
| rs11206510 | 2.34E-08 | 0.225 | 0.521 | | 0.489 | | 0.206 | |  |
| rs72664303 | 7.12E-11 | 0.207 | 0.0948 | | 0.0244 | | 0.961 | |  |
| rs72664304 | 5.65E-11 | 0.224 | 0.0803 | | 0.0228 | | 0.932 | |  |
| rs55694910 | 3.84E-11 | 0.219 | 0.1 | | 0.0184 | | 0.972 | |  |
| rs17114036 | 2.22E-13 | 0.17 | 0.227 | | 0.0159 | | 0.774 | |  |
| rs72664318 | 1.18E-13 | 0.207 | 0.209 | | 0.0134 | | 0.812 | |  |
| rs9970807 | 5.00E-14 | 0.21 | 0.156 | | 0.0159 | | 0.883 | |  |
| rs17114046 | 6.06E-14 | 0.228 | 0.167 | | 0.0169 | | 0.919 | |  |
| rs56186267 | 1.50E-13 | 0.25 | 0.136 | | 0.017 | | 0.799 | |  |
| rs72664324 | 1.67E-13 | 0.226 | 0.165 | | 0.0183 | | 0.631 | |  |
| rs2404716 | 9.57E-10 | 0.859 | 0.202 | | 0.104 | | 0.889 | |  |
| rs72664332 | 1.40E-13 | 0.162 | 0.147 | | 0.0118 | | 0.735 | |  |
| rs55869368 | 3.05E-13 | 0.163 | 0.122 | | 0.0107 | | 0.742 | |  |
| rs72664335 | 1.05E-13 | 0.161 | 0.128 | | 0.00996 | | 0.745 | |  |
| rs56348932 | 1.17E-13 | 0.149 | 0.132 | | 0.011 | | 0.732 | |  |
| rs72664341 | 2.31E-13 | 0.188 | 0.146 | | 0.0139 | | 0.684 | |  |
| rs56322312 | 3.03E-13 | 0.214 | 0.116 | | 0.0127 | | 0.679 | |  |
| rs12082242 | 4.18E-08 | 0.944 | 0.34 | | 0.0281 | | 0.0814 | |  |
| rs4634932 | 9.41E-14 | 0.183 | 0.206 | | 0.00295 | | 0.665 | |  |
| rs953857 | 1.76E-08 | 0.687 | 0.505 | | 0.0254 | | 0.183 | |  |
| rs953856 | 3.59E-08 | 0.909 | 0.513 | | 0.0138 | | 0.0902 | |  |
| rs10888977 | 2.74E-08 | 0.98 | 0.339 | | 0.026 | | 0.0824 | |  |
| rs2017955 | 1.80E-08 | 0.668 | 0.517 | | 0.0254 | | 0.185 | |  |
| rs72664353 | 1.98E-13 | 0.177 | 0.102 | | 0.00903 | | 0.786 | |  |
| rs72664355 | 1.03E-13 | 0.18 | 0.0972 | | 0.00871 | | 0.863 | |  |
| rs2404715 | 1.55E-13 | 0.179 | 0.0896 | | 0.00853 | | 0.83 | |  |
| rs12134109 | 9.85E-09 | 0.707 | 0.479 | | 0.0236 | | 0.18 | |  |
| rs11206838 | 1.68E-08 | 0.675 | 0.497 | | 0.0217 | | 0.166 | |  |
| rs72664358 | 3.13E-08 | 0.419 | 0.229 | | 0.0267 | | 0.42 | |  |
| rs56170783 | 9.84E-14 | 0.171 | 0.0902 | | 0.0103 | | 0.878 | |  |
| rs7551402 | 2.59E-08 | 0.614 | 0.457 | | 0.0354 | | 0.192 | |  |
| rs55751848 | 3.39E-08 | 0.941 | 0.421 | | 0.182 | | 0.81 | |  |
| rs72664362 | 1.07E-08 | 0.542 | 0.601 | | 0.0636 | | 0.521 | |  |
| rs6657811 | 2.74E-08 | 0.422 | 0.847 | | 0.684 | | 0.791 | |  |
| rs4970834 | 7.33E-15 | 0.61 | 0.64 | | 0.0817 | | 0.506 | |  |
| rs611917 | 5.40E-14 | 0.718 | 0.607 | | 0.0482 | | 0.254 | |  |
| rs7528419 | 1.97E-23 | 0.594 | 0.888 | | 0.247 | | 0.976 | |  |
| rs11102967 | 4.48E-11 | 0.678 | 0.684 | | 0.043 | | 0.204 | |  |
| rs12740374 | 4.63E-23 | 0.578 | 0.896 | | 0.267 | | 0.934 | |  |
| rs660240 | 8.66E-19 | 0.729 | 0.84 | | 0.255 | | 0.938 | |  |
| rs629301 | 6.66E-19 | 0.647 | 0.939 | | 0.275 | | 0.948 | |  |
| rs646776 | 9.01E-19 | 0.621 | 0.927 | | 0.273 | | 0.925 | |  |
| rs3902354 | 2.13E-11 | 0.674 | 0.591 | | 0.0586 | | 0.292 | |  |
| rs583104 | 7.25E-17 | 0.734 | 0.885 | | 0.272 | | 0.801 | |  |
| rs602633 | 6.97E-17 | 0.769 | 0.837 | | 0.17 | | 0.844 | |  |
| rs4970836 | 1.11E-16 | 0.756 | 0.943 | | 0.265 | | 0.961 | |  |
| rs4970837 | 2.97E-11 | 0.898 | 0.609 | | 0.0553 | | 0.181 | |  |
| rs1277930 | 3.28E-17 | 0.764 | 0.951 | | 0.264 | | 0.799 | |  |
| rs599839 | 5.20E-17 | 0.767 | 0.943 | | 0.261 | | 0.732 | |  |
| rs11265611 | 1.35E-08 | 0.997 | 0.338 | | 0.0122 | | 0.417 | |  |
| rs6689306 | 2.60E-09 | 0.922 | 0.619 | | 0.0179 | | 0.536 | |  |
| rs12118721 | 5.27E-09 | 0.69 | 0.451 | | 0.0145 | | 0.576 | |  |
| rs12118770 | 3.15E-09 | 0.845 | 0.533 | | 0.0156 | | 0.614 | |  |
| rs12117832 | 1.60E-08 | 0.726 | 0.393 | | 0.0165 | | 0.561 | |  |
| rs10908838 | 6.93E-09 | 0.79 | 0.541 | | 0.0145 | | 0.664 | |  |
| rs4845618 | 9.20E-09 | 0.684 | 0.404 | | 0.0115 | | 0.582 | |  |
| rs6687726 | 6.59E-09 | 0.672 | 0.419 | | 0.0113 | | 0.603 | |  |
| rs6427658 | 5.49E-09 | 0.771 | 0.511 | | 0.0122 | | 0.694 | |  |
| rs6694817 | 2.96E-09 | 0.8 | 0.432 | | 0.0121 | | 0.712 | |  |
| rs7549250 | 8.45E-09 | 0.724 | 0.301 | | 0.0091 | | 0.578 | |  |
| rs7549338 | 7.60E-09 | 0.871 | 0.417 | | 0.00851 | | 0.621 | |  |
| rs7553796 | 6.19E-09 | 0.871 | 0.426 | | 0.0089 | | 0.632 | |  |
| rs4845619 | 5.76E-09 | 0.716 | 0.344 | | 0.00827 | | 0.563 | |  |
| rs59632925 | 6.83E-09 | 0.875 | 0.416 | | 0.00909 | | 0.668 | |  |
| rs4845620 | 7.64E-08 | 0.753 | 0.19 | | 0.397 | | 0.271 | |  |
| rs7521458 | 5.70E-08 | 0.789 | 0.179 | | 0.362 | | 0.275 | |  |
| rs4845371 | 1.03E-08 | 0.869 | 0.426 | | 0.00958 | | 0.667 | |  |
| rs6667434 | 1.10E-08 | 0.882 | 0.409 | | 0.00885 | | 0.682 | |  |
| rs4845621 | 6.52E-08 | 0.761 | 0.195 | | 0.398 | | 0.277 | |  |
| rs4553185 | 7.51E-09 | 0.774 | 0.31 | | 0.00898 | | 0.591 | |  |
| rs4845622 | 9.03E-08 | 0.768 | 0.202 | | 0.403 | | 0.289 | |  |
| rs11265612 | 3.74E-08 | 0.912 | 0.462 | | 0.0097 | | 0.661 | |  |
| rs4845373 | 5.16E-08 | 0.781 | 0.198 | | 0.39 | | 0.317 | |  |
| rs11265613 | 4.96E-08 | 0.823 | 0.161 | | 0.304 | | 0.263 | |  |
| rs6686750 | 2.10E-08 | 0.854 | 0.721 | | 0.00429 | | 0.784 | |  |
| rs4845625 | 3.93E-08 | 0.89 | 0.75 | | 0.00286 | | 0.76 | |  |
| rs12129500 | 6.01E-08 | 0.894 | 0.739 | | 0.00287 | | 0.758 | |  |
| rs6689393 | 8.39E-08 | 0.932 | 0.73 | | 0.00229 | | 0.758 | |  |
| rs6694258 | 4.83E-08 | 0.982 | 0.744 | | 0.00213 | | 0.726 | |  |
| rs4618978 | 1.81E-11 | 0.18 | 0.0333 | | 0.53 | | 0.45 | |  |
| rs34679168 | 1.90E-08 | 0.495 | 0.0315 | | 0.0952 | | 0.193 | |  |
| rs1909196 | 4.02E-10 | 0.34 | 0.0109 | | 0.349 | | 0.299 | |  |
| rs2378584 | 1.45E-11 | 0.783 | 0.0326 | | 0.375 | | 0.34 | |  |
| rs34767248 | 1.49E-11 | 0.809 | 0.0323 | | 0.367 | | 0.328 | |  |
| rs17163301 | 1.47E-11 | 0.808 | 0.0326 | | 0.36 | | 0.331 | |  |
| rs4846769 | 2.16E-11 | 0.978 | 0.0341 | | 0.405 | | 0.343 | |  |
| rs4846770 | 2.90E-11 | 0.918 | 0.036 | | 0.375 | | 0.37 | |  |
| rs4846384 | 1.04E-11 | 0.787 | 0.0353 | | 0.334 | | 0.358 | |  |
| rs17163313 | 2.45E-12 | 0.804 | 0.046 | | 0.37 | | 0.31 | |  |
| rs3748626 | 4.66E-12 | 0.744 | 0.0518 | | 0.413 | | 0.288 | |  |
| rs17163345 | 3.65E-12 | 0.729 | 0.0454 | | 0.396 | | 0.287 | |  |
| rs35700460 | 1.38E-12 | 0.702 | 0.157 | | 0.238 | | 0.24 | |  |
| rs2133187 | 3.27E-12 | 0.689 | 0.165 | | 0.23 | | 0.241 | |  |
| rs2133189 | 2.42E-12 | 0.589 | 0.188 | | 0.247 | | 0.217 | |  |
| rs35626308 | 1.89E-12 | 0.707 | 0.168 | | 0.248 | | 0.256 | |  |
| rs1909197 | 6.37E-08 | 0.685 | 0.119 | | 0.197 | | 0.745 | |  |
| rs17163358 | 2.66E-12 | 0.706 | 0.172 | | 0.254 | | 0.245 | |  |
| rs17163360 | 1.90E-12 | 0.704 | 0.178 | | 0.253 | | 0.248 | |  |
| rs61824282 | 8.80E-10 | 0.355 | 0.165 | | 0.109 | | 0.119 | |  |
| rs28709375 | 3.82E-12 | 0.586 | 0.172 | | 0.212 | | 0.235 | |  |
| rs17465637 | 3.52E-12 | 0.584 | 0.156 | | 0.212 | | 0.225 | |  |
| rs67180937 | 1.01E-12 | 0.641 | 0.149 | | 0.223 | | 0.258 | |  |
| rs17011681 | 1.81E-12 | 0.712 | 0.179 | | 0.245 | | 0.248 | |  |
| rs10495198 | 3.46E-12 | 0.587 | 0.166 | | 0.213 | | 0.231 | |  |
| rs2291832 | 2.28E-12 | 0.591 | 0.166 | | 0.226 | | 0.243 | |  |
| rs17163363 | 1.23E-12 | 0.579 | 0.161 | | 0.213 | | 0.229 | |  |
| rs35158675 | 1.03E-12 | 0.558 | 0.164 | | 0.213 | | 0.232 | |  |
| rs2291834 | 1.60E-12 | 0.576 | 0.166 | | 0.216 | | 0.221 | |  |
| rs4575092 | 2.30E-12 | 0.689 | 0.179 | | 0.25 | | 0.247 | |  |
| rs1133220 | 4.24E-12 | 0.59 | 0.157 | | 0.214 | | 0.242 | |  |
| rs17465940 | 7.47E-12 | 0.705 | 0.174 | | 0.234 | | 0.295 | |  |
| rs2270707 | 7.07E-08 | 0.686 | 0.139 | | 0.189 | | 0.759 | |  |
| rs17465982 | 1.09E-11 | 0.687 | 0.184 | | 0.243 | | 0.243 | |  |
| rs12138316 | 2.72E-11 | 0.743 | 0.168 | | 0.319 | | 0.286 | |  |
| rs34032254 | 2.84E-11 | 0.655 | 0.323 | | 0.373 | | 0.288 | |  |
| rs35175385 | 2.84E-11 | 0.653 | 0.323 | | 0.369 | | 0.285 | |  |
| rs71524942 | 3.95E-11 | 0.724 | 0.191 | | 0.295 | | 0.278 | |  |
| rs2378597 | 6.43E-09 | 0.305 | 0.244 | | 0.16 | | 0.146 | |  |
| rs61824331 | 1.32E-09 | 0.931 | 0.22 | | 0.406 | | 0.203 | |  |
| rs17532708 | 3.14E-09 | 0.86 | 0.246 | | 0.374 | | 0.183 | |  |
| rs12043288 | 4.80E-09 | 0.867 | 0.241 | | 0.363 | | 0.185 | |  |
| rs61824334 | 9.06E-08 | 0.472 | 0.306 | | 0.163 | | 0.121 | |  |
| rs4282830 | 4.34E-09 | 0.841 | 0.235 | | 0.362 | | 0.194 | |  |
| rs2378607 | 5.56E-10 | 0.715 | 0.214 | | 0.358 | | 0.144 | |  |
| rs2068814 | 8.85E-10 | 0.763 | 0.202 | | 0.345 | | 0.147 | |  |
| rs34885880 | 6.54E-10 | 0.747 | 0.19 | | 0.327 | | 0.161 | |  |
| rs12749296 | 6.63E-10 | 0.734 | 0.188 | | 0.335 | | 0.157 | |  |
| rs61825253 | 7.66E-10 | 0.566 | 0.118 | | 0.305 | | 0.227 | |  |
| rs61825254 | 1.30E-09 | 0.509 | 0.127 | | 0.325 | | 0.183 | |  |
| rs28521319 | 4.50E-10 | 0.51 | 0.124 | | 0.302 | | 0.192 | |  |
| rs28654270 | 1.64E-09 | 0.578 | 0.124 | | 0.304 | | 0.191 | |  |
| rs61825258 | 4.08E-09 | 0.539 | 0.145 | | 0.331 | | 0.169 | |  |
| rs61825259 | 1.30E-08 | 0.621 | 0.141 | | 0.34 | | 0.203 | |  |
| rs6700083 | 2.03E-09 | 0.527 | 0.114 | | 0.324 | | 0.178 | |  |
| rs146451521 | 3.59E-08 | 0.537 | 0.119 | | 0.346 | | 0.16 | |  |
| rs35300015 | 5.97E-09 | 0.513 | 0.103 | | 0.343 | | 0.178 | |  |
| rs2378603 | 2.64E-08 | 0.534 | 0.143 | | 0.344 | | 0.149 | |  |
| rs16986953 | 1.45E-08 | 0.308 | 0.803 | | 0.773 | | 0.0607 | |  |
| rs531819 | 5.61E-08 | 0.745 | 0.529 | | 0.98 | | 0.292 | |  |
| rs585967 | 6.53E-08 | 0.906 | 0.484 | | 0.937 | | 0.281 | |  |
| rs515135 | 3.09E-08 | 0.135 | 0.146 | | 0.467 | | 0.249 | |  |
| rs563290 | 5.08E-08 | 0.159 | 0.156 | | 0.443 | | 0.305 | |  |
| rs562338 | 7.81E-08 | 0.16 | 0.136 | | 0.454 | | 0.274 | |  |
| rs581411 | 3.83E-08 | 0.171 | 0.147 | | 0.442 | | 0.297 | |  |
| rs548145 | 3.73E-08 | 0.175 | 0.158 | | 0.355 | | 0.306 | |  |
| rs668948 | 5.74E-08 | 0.154 | 0.155 | | 0.348 | | 0.259 | |  |
| rs541041 | 5.07E-08 | 0.144 | 0.161 | | 0.34 | | 0.27 | |  |
| rs503105 | 6.59E-08 | 0.0478 | 0.0497 | | 0.333 | | 0.216 | |  |
| rs1712246 | 4.47E-08 | 0.0627 | 0.104 | | 0.248 | | 0.205 | |  |
| rs7591175 | 1.07E-08 | 0.0151 | 0.945 | | 0.413 | | 0.477 | |  |
| rs13411109 | 3.87E-08 | 0.0278 | 0.429 | | 0.61 | | 0.694 | |  |
| rs2166529 | 1.54E-08 | 0.0203 | 0.919 | | 0.27 | | 0.9 | |  |
| rs2886722 | 9.57E-08 | 0.0553 | 0.287 | | 0.756 | | 0.616 | |  |
| rs60012532 | 5.18E-08 | 0.0375 | 0.0416 | | 0.244 | | 0.421 | |  |
| rs11895399 | 7.21E-08 | 0.0185 | 0.0391 | | 0.296 | | 0.398 | |  |
| rs11895401 | 7.13E-08 | 0.0191 | 0.0384 | | 0.298 | | 0.406 | |  |
| rs13394343 | 6.32E-09 | 0.0234 | 0.225 | | 0.974 | | 0.409 | |  |
| rs6735152 | 9.54E-09 | 0.0242 | 0.223 | | 0.961 | | 0.414 | |  |
| rs6721924 | 1.00E-08 | 0.0228 | 0.221 | | 0.965 | | 0.413 | |  |
| rs6739015 | 8.54E-09 | 0.0229 | 0.217 | | 0.96 | | 0.406 | |  |
| rs6547620 | 9.51E-09 | 0.0227 | 0.225 | | 0.96 | | 0.42 | |  |
| rs6714709 | 8.80E-09 | 0.0243 | 0.222 | | 0.966 | | 0.423 | |  |
| rs6719046 | 8.84E-09 | 0.0237 | 0.205 | | 0.994 | | 0.395 | |  |
| rs6733913 | 8.84E-09 | 0.0239 | 0.205 | | 0.989 | | 0.396 | |  |
| rs6750847 | 6.91E-09 | 0.0113 | 0.252 | | 0.857 | | 0.763 | |  |
| rs6722691 | 6.12E-08 | 0.0176 | 0.0357 | | 0.306 | | 0.395 | |  |
| rs2044474 | 8.02E-09 | 0.0232 | 0.222 | | 0.964 | | 0.41 | |  |
| rs17026396 | 7.89E-09 | 0.024 | 0.215 | | 0.959 | | 0.407 | |  |
| rs59877521 | 7.66E-09 | 0.0242 | 0.223 | | 0.951 | | 0.413 | |  |
| rs6731005 | 1.80E-09 | 0.0317 | 0.85 | | 0.625 | | 0.312 | |  |
| rs6705839 | 1.88E-09 | 0.0666 | 0.932 | | 0.565 | | 0.723 | |  |
| rs6705971 | 4.52E-10 | 0.0297 | 0.904 | | 0.687 | | 0.294 | |  |
| rs10175792 | 6.59E-10 | 0.0298 | 0.906 | | 0.681 | | 0.294 | |  |
| rs10176176 | 4.99E-10 | 0.0705 | 0.756 | | 0.58 | | 0.505 | |  |
| rs1446669 | 4.66E-08 | 0.0195 | 0.0302 | | 0.349 | | 0.411 | |  |
| rs10179195 | 5.22E-10 | 0.0295 | 0.897 | | 0.656 | | 0.303 | |  |
| rs6714157 | 4.60E-09 | 0.0302 | 0.901 | | 0.656 | | 0.303 | |  |
| rs6743030 | 4.51E-10 | 0.0316 | 0.901 | | 0.669 | | 0.298 | |  |
| rs3755014 | 1.05E-09 | 0.0327 | 0.886 | | 0.646 | | 0.315 | |  |
| rs3755015 | 1.07E-09 | 0.0314 | 0.902 | | 0.643 | | 0.316 | |  |
| rs1446668 | 8.72E-10 | 0.0241 | 0.884 | | 0.623 | | 0.314 | |  |
| rs2028900 | 2.94E-09 | 0.162 | 0.793 | | 0.815 | | 0.396 | |  |
| rs1078004 | 6.16E-10 | 0.0525 | 0.921 | | 0.445 | | 0.776 | |  |
| rs7605975 | 5.14E-10 | 0.0582 | 0.78 | | 0.603 | | 0.274 | |  |
| rs12473819 | 1.00E-09 | 0.0481 | 0.822 | | 0.586 | | 0.308 | |  |
| rs6547621 | 5.37E-10 | 0.0578 | 0.775 | | 0.596 | | 0.286 | |  |
| rs699664 | 6.00E-08 | 0.195 | 0.269 | | 0.556 | | 0.298 | |  |
| rs6738645 | 5.60E-10 | 0.0518 | 0.762 | | 0.615 | | 0.285 | |  |
| rs12714145 | 8.72E-09 | 0.136 | 0.214 | | 0.846 | | 0.482 | |  |
| rs7568458 | 3.62E-10 | 0.0159 | 0.467 | | 0.732 | | 0.414 | |  |
| rs10172544 | 9.05E-09 | 0.131 | 0.214 | | 0.833 | | 0.478 | |  |
| rs35215812 | 2.90E-09 | 0.0387 | 0.221 | | 0.937 | | 0.353 | |  |
| rs10187424 | 1.73E-09 | 0.0448 | 0.187 | | 0.976 | | 0.389 | |  |
| rs10198569 | 2.98E-09 | 0.0429 | 0.202 | | 0.96 | | 0.399 | |  |
| rs11891260 | 1.32E-09 | 0.0674 | 0.662 | | 0.554 | | 0.367 | |  |
| rs6547624 | 5.24E-09 | 0.0481 | 0.239 | | 0.946 | | 0.406 | |  |
| rs6757263 | 1.60E-09 | 0.0498 | 0.228 | | 0.972 | | 0.366 | |  |
| rs3770098 | 1.83E-09 | 0.0594 | 0.227 | | 0.998 | | 0.407 | |  |
| rs3731827 | 1.16E-09 | 0.0598 | 0.221 | | 0.986 | | 0.415 | |  |
| rs1972297 | 9.20E-10 | 0.0642 | 0.221 | | 1 | | 0.384 | |  |
| rs1009 | 9.30E-10 | 0.0627 | 0.206 | | 0.987 | | 0.388 | |  |
| rs1058588 | 1.02E-09 | 0.0654 | 0.219 | | 0.998 | | 0.382 | |  |
| rs1562323 | 1.48E-09 | 0.0658 | 0.205 | | 0.978 | | 0.37 | |  |
| rs2366639 | 9.78E-10 | 0.0636 | 0.213 | | 0.995 | | 0.389 | |  |
| rs1561198 | 6.37E-10 | 0.0918 | 0.639 | | 0.746 | | 0.382 | |  |
| rs55971080 | 2.48E-09 | 0.0743 | 0.464 | | 0.789 | | 0.307 | |  |
| rs10206961 | 8.12E-08 | 0.246 | 0.463 | | 0.559 | | 0.858 | |  |
| rs17678683 | 3.00E-09 | 0.268 | 0.726 | | 0.262 | | 0.576 | |  |
| rs17740744 | 3.25E-09 | 0.557 | 0.976 | | 0.337 | | 0.53 | |  |
| rs146289328 | 4.14E-08 | 0.432 | 0.0196 | | 0.533 | | 0.765 | |  |
| rs72932707 | 7.17E-17 | 0.0147 | 0.00042 | | 0.0923 | | 0.435 | |  |
| rs72932709 | 1.11E-16 | 0.0161 | 0.000403 | | 0.0885 | | 0.439 | |  |
| rs4675290 | 1.17E-08 | 0.254 | 2.09E-06 | | 0.0197 | | 0.268 | |  |
| rs72932711 | 1.11E-16 | 0.0154 | 0.000399 | | 0.0884 | | 0.441 | |  |
| rs2036927 | 1.40E-08 | 0.24 | 2.13E-06 | | 0.0197 | | 0.245 | |  |
| rs72932716 | 7.00E-17 | 0.0152 | 0.000397 | | 0.09 | | 0.427 | |  |
| rs72932720 | 2.22E-16 | 0.014 | 0.000387 | | 0.103 | | 0.435 | |  |
| rs11675462 | 4.04E-09 | 0.192 | 7.69E-07 | | 0.0126 | | 0.224 | |  |
| rs72932722 | 1.11E-16 | 0.0147 | 0.000433 | | 0.0889 | | 0.406 | |  |
| rs72932723 | 1.11E-16 | 0.015 | 0.000445 | | 0.0896 | | 0.416 | |  |
| rs72932725 | 2.22E-16 | 0.016 | 0.000711 | | 0.0327 | | 0.381 | |  |
| rs72932727 | 1.11E-16 | 0.0265 | 0.000801 | | 0.0283 | | 0.463 | |  |
| rs72932731 | 1.11E-16 | 0.0156 | 0.000682 | | 0.0325 | | 0.392 | |  |
| rs143911965 | 1.11E-16 | 0.0153 | 0.000712 | | 0.0333 | | 0.383 | |  |
| rs72932737 | 7.51E-17 | 0.0146 | 0.000682 | | 0.0356 | | 0.386 | |  |
| rs6732078 | 6.75E-17 | 0.0151 | 0.000748 | | 0.0329 | | 0.394 | |  |
| rs6435168 | 7.19E-17 | 0.0151 | 0.000745 | | 0.033 | | 0.394 | |  |
| rs72932741 | 7.75E-17 | 0.0159 | 0.000786 | | 0.0334 | | 0.385 | |  |
| rs6705330 | 2.22E-16 | 0.0155 | 0.000768 | | 0.0332 | | 0.393 | |  |
| rs72932745 | 6.72E-17 | 0.015 | 0.000796 | | 0.0341 | | 0.384 | |  |
| rs72932746 | 7.34E-17 | 0.0149 | 0.000763 | | 0.0335 | | 0.399 | |  |
| rs78128841 | 6.53E-17 | 0.0151 | 0.000777 | | 0.0343 | | 0.403 | |  |
| rs76122535 | 1.55E-15 | 0.00985 | 0.00043 | | 0.0516 | | 0.295 | |  |
| rs72932752 | 1.50E-17 | 0.0159 | 0.000776 | | 0.039 | | 0.405 | |  |
| rs72932753 | 2.80E-17 | 0.00788 | 0.000779 | | 0.0412 | | 0.382 | |  |
| rs144505847 | 3.78E-17 | 0.00778 | 0.000783 | | 0.0417 | | 0.376 | |  |
| rs80087860 | 2.22E-16 | 0.00748 | 0.000765 | | 0.0412 | | 0.38 | |  |
| rs72932755 | 4.33E-17 | 0.00706 | 0.000788 | | 0.0406 | | 0.376 | |  |
| rs72932759 | 4.57E-17 | 0.00761 | 0.000843 | | 0.0395 | | 0.365 | |  |
| rs115400054 | 5.55E-16 | 0.0215 | 0.00137 | | 0.0495 | | 0.459 | |  |
| rs72932763 | 3.38E-17 | 0.0087 | 0.000953 | | 0.0391 | | 0.374 | |  |
| rs72932765 | 3.66E-17 | 0.00923 | 0.000779 | | 0.0388 | | 0.385 | |  |
| rs72932767 | 3.32E-17 | 0.00858 | 0.00106 | | 0.0361 | | 0.378 | |  |
| rs72932770 | 3.10E-17 | 0.00889 | 0.00104 | | 0.0366 | | 0.373 | |  |
| rs72932772 | 3.09E-17 | 0.0101 | 0.00132 | | 0.0379 | | 0.363 | |  |
| rs79633844 | 1.11E-16 | 0.00896 | 0.00118 | | 0.0373 | | 0.347 | |  |
| rs75141346 | 6.21E-17 | 0.0107 | 0.00129 | | 0.0375 | | 0.349 | |  |
| rs72932774 | 2.86E-17 | 0.0108 | 0.00136 | | 0.0389 | | 0.354 | |  |
| rs72932776 | 2.32E-17 | 0.0114 | 0.00145 | | 0.0382 | | 0.356 | |  |
| rs72932777 | 2.02E-17 | 0.0117 | 0.00151 | | 0.0376 | | 0.349 | |  |
| rs115628302 | 2.13E-17 | 0.0118 | 0.00151 | | 0.0377 | | 0.35 | |  |
| rs75166090 | 3.93E-17 | 0.0118 | 0.00164 | | 0.0384 | | 0.351 | |  |
| rs78488377 | 1.78E-09 | 0.0911 | 0.00981 | | 0.611 | | 0.745 | |  |
| rs143805266 | 1.68E-17 | 0.012 | 0.00165 | | 0.0387 | | 0.348 | |  |
| rs72932780 | 1.65E-17 | 0.0118 | 0.00162 | | 0.0397 | | 0.351 | |  |
| rs114520702 | 1.14E-17 | 0.0149 | 0.00218 | | 0.0479 | | 0.419 | |  |
| rs72932781 | 1.24E-17 | 0.0137 | 0.0019 | | 0.0444 | | 0.353 | |  |
| rs75324925 | 1.16E-17 | 0.0138 | 0.00189 | | 0.0443 | | 0.35 | |  |
| rs72932784 | 1.15E-17 | 0.0138 | 0.00188 | | 0.0443 | | 0.351 | |  |
| rs72932786 | 1.10E-17 | 0.0138 | 0.00188 | | 0.0442 | | 0.35 | |  |
| rs72932789 | 1.10E-17 | 0.0139 | 0.00189 | | 0.0444 | | 0.348 | |  |
| rs144630409 | 1.06E-17 | 0.0142 | 0.00185 | | 0.0404 | | 0.361 | |  |
| rs72932791 | 1.11E-17 | 0.0143 | 0.00185 | | 0.0417 | | 0.356 | |  |
| rs72932793 | 1.17E-17 | 0.014 | 0.00188 | | 0.0415 | | 0.347 | |  |
| rs1971819 | 4.96E-11 | 0.0223 | 8.81E-06 | | 0.00976 | | 0.0208 | |  |
| rs72934704 | 8.69E-18 | 0.0136 | 0.00172 | | 0.043 | | 0.35 | |  |
| rs72934706 | 1.26E-17 | 0.0147 | 0.0017 | | 0.0439 | | 0.342 | |  |
| rs72934707 | 7.95E-18 | 0.0251 | 0.00184 | | 0.0445 | | 0.345 | |  |
| rs934287 | 3.60E-12 | 0.0205 | 1.10E-05 | | 0.0123 | | 0.0197 | |  |
| rs72934710 | 8.11E-18 | 0.0132 | 0.00185 | | 0.0467 | | 0.337 | |  |
| rs72934711 | 7.91E-18 | 0.0138 | 0.00179 | | 0.044 | | 0.344 | |  |
| rs72934714 | 7.94E-18 | 0.0139 | 0.00181 | | 0.0439 | | 0.342 | |  |
| rs72934715 | 8.13E-18 | 0.0144 | 0.00174 | | 0.0431 | | 0.335 | |  |
| rs10207567 | 2.86E-11 | 0.0222 | 8.62E-06 | | 0.00858 | | 0.0215 | |  |
| rs140154196 | 7.75E-18 | 0.014 | 0.00161 | | 0.0462 | | 0.331 | |  |
| rs114372659 | 7.77E-18 | 0.014 | 0.00166 | | 0.0465 | | 0.33 | |  |
| rs146900385 | 7.74E-18 | 0.0141 | 0.00159 | | 0.0477 | | 0.33 | |  |
| rs114155121 | 7.32E-18 | 0.0137 | 0.0016 | | 0.0477 | | 0.328 | |  |
| rs143385727 | 7.36E-18 | 0.0135 | 0.00159 | | 0.0476 | | 0.327 | |  |
| rs12693975 | 1.83E-11 | 0.0274 | 8.13E-06 | | 0.00992 | | 0.0225 | |  |
| rs147100405 | 3.85E-17 | 0.026 | 0.00151 | | 0.0509 | | 0.332 | |  |
| rs115396314 | 5.11E-18 | 0.0139 | 0.0016 | | 0.0498 | | 0.326 | |  |
| rs114139737 | 9.00E-18 | 0.014 | 0.00159 | | 0.05 | | 0.326 | |  |
| rs7605484 | 7.41E-18 | 0.0371 | 0.00161 | | 0.0496 | | 0.343 | |  |
| rs115130739 | 3.38E-17 | 0.0138 | 0.00168 | | 0.0513 | | 0.315 | |  |
| rs115827549 | 2.84E-17 | 0.0138 | 0.0017 | | 0.0515 | | 0.314 | |  |
| rs145643482 | 7.56E-18 | 0.0141 | 0.00164 | | 0.0515 | | 0.325 | |  |
| rs72934725 | 6.68E-18 | 0.0141 | 0.00158 | | 0.0523 | | 0.325 | |  |
| rs140606214 | 4.80E-17 | 0.0136 | 0.00121 | | 0.0536 | | 0.316 | |  |
| rs145664840 | 2.61E-17 | 0.0141 | 0.00112 | | 0.0503 | | 0.335 | |  |
| rs150559122 | 6.73E-18 | 0.0237 | 0.00159 | | 0.0533 | | 0.328 | |  |
| rs147361301 | 6.68E-18 | 0.0147 | 0.00161 | | 0.0528 | | 0.325 | |  |
| rs144997119 | 9.33E-18 | 0.0149 | 0.00154 | | 0.056 | | 0.336 | |  |
| rs7608755 | 1.77E-17 | 0.0147 | 0.00157 | | 0.0553 | | 0.305 | |  |
| rs3845800 | 1.27E-17 | 0.0144 | 0.00174 | | 0.054 | | 0.305 | |  |
| rs4510208 | 7.55E-18 | 0.0148 | 0.00176 | | 0.0553 | | 0.297 | |  |
| rs149846585 | 2.08E-17 | 0.0247 | 0.00152 | | 0.0564 | | 0.303 | |  |
| rs72934729 | 7.92E-18 | 0.0156 | 0.00185 | | 0.0584 | | 0.31 | |  |
| rs6723704 | 7.92E-18 | 0.0159 | 0.00165 | | 0.0566 | | 0.335 | |  |
| rs72934732 | 8.00E-18 | 0.016 | 0.00187 | | 0.0619 | | 0.307 | |  |
| rs72934734 | 7.57E-18 | 0.016 | 0.000697 | | 0.0857 | | 0.522 | |  |
| rs72934735 | 8.25E-18 | 0.016 | 0.00187 | | 0.0621 | | 0.307 | |  |
| rs146973310 | 7.99E-18 | 0.0161 | 0.00188 | | 0.0618 | | 0.308 | |  |
| rs72934737 | 7.25E-18 | 0.0162 | 0.00186 | | 0.0625 | | 0.305 | |  |
| rs72934738 | 8.31E-18 | 0.0162 | 0.00186 | | 0.0626 | | 0.305 | |  |
| rs79539678 | 7.79E-18 | 0.0162 | 0.00187 | | 0.0627 | | 0.305 | |  |
| rs72934740 | 7.70E-18 | 0.0169 | 0.00184 | | 0.0647 | | 0.304 | |  |
| rs115953525 | 6.55E-18 | 0.0156 | 0.00189 | | 0.0633 | | 0.277 | |  |
| rs72934745 | 1.81E-17 | 0.0158 | 0.00184 | | 0.0631 | | 0.28 | |  |
| rs148513392 | 2.22E-16 | 0.0304 | 0.00248 | | 0.0709 | | 0.367 | |  |
| rs6722332 | 6.41E-18 | 0.0163 | 0.00174 | | 0.0679 | | 0.304 | |  |
| rs6725887 | 9.51E-18 | 0.0167 | 0.00146 | | 0.0616 | | 0.286 | |  |
| rs72934749 | 3.91E-17 | 0.0168 | 0.00145 | | 0.0646 | | 0.327 | |  |
| rs72934751 | 6.20E-18 | 0.0168 | 0.00144 | | 0.0667 | | 0.328 | |  |
| rs143035655 | 6.15E-18 | 0.0163 | 0.00148 | | 0.067 | | 0.322 | |  |
| rs6738618 | 6.99E-18 | 0.0168 | 0.00145 | | 0.0646 | | 0.326 | |  |
| rs72934753 | 7.54E-18 | 0.0173 | 0.00152 | | 0.0647 | | 0.316 | |  |
| rs116382857 | 7.55E-18 | 0.0168 | 0.00149 | | 0.0669 | | 0.32 | |  |
| rs77268589 | 8.73E-18 | 0.0171 | 0.00148 | | 0.0655 | | 0.327 | |  |
| rs6435169 | 7.54E-18 | 0.017 | 0.00146 | | 0.0646 | | 0.326 | |  |
| rs7582720 | 7.52E-18 | 0.0171 | 0.00142 | | 0.0654 | | 0.326 | |  |
| rs72934760 | 5.55E-16 | 0.0434 | 0.00495 | | 0.622 | | 0.229 | |  |
| rs72934762 | 9.85E-18 | 0.0173 | 0.00149 | | 0.0651 | | 0.322 | |  |
| rs7560547 | 7.17E-18 | 0.0169 | 0.00146 | | 0.0653 | | 0.325 | |  |
| rs72934763 | 8.57E-18 | 0.0169 | 0.00144 | | 0.0652 | | 0.324 | |  |
| rs72934764 | 6.77E-18 | 0.0177 | 0.00156 | | 0.0636 | | 0.318 | |  |
| rs72934765 | 6.51E-18 | 0.0179 | 0.00159 | | 0.0632 | | 0.317 | |  |
| rs150788469 | 8.16E-18 | 0.0171 | 0.00145 | | 0.0655 | | 0.324 | |  |
| rs77931721 | 1.44E-17 | 0.0165 | 0.00146 | | 0.0656 | | 0.322 | |  |
| rs35212307 | 1.41E-17 | 0.0175 | 0.00107 | | 0.0633 | | 0.323 | |  |
| rs72934767 | 1.41E-17 | 0.0165 | 0.00158 | | 0.0621 | | 0.331 | |  |
| rs142603618 | 1.48E-17 | 0.0158 | 0.00146 | | 0.0648 | | 0.316 | |  |
| rs114395475 | 1.48E-17 | 0.0163 | 0.00141 | | 0.0673 | | 0.316 | |  |
| rs72936830 | 1.48E-17 | 0.0159 | 0.00147 | | 0.0652 | | 0.318 | |  |
| rs72936834 | 1.38E-17 | 0.0159 | 0.00153 | | 0.066 | | 0.314 | |  |
| rs114899426 | 1.46E-17 | 0.0159 | 0.00148 | | 0.0653 | | 0.315 | |  |
| rs72936838 | 1.32E-17 | 0.0158 | 0.00151 | | 0.0646 | | 0.315 | |  |
| rs72936839 | 1.50E-17 | 0.0159 | 0.00148 | | 0.0641 | | 0.316 | |  |
| rs72936842 | 1.48E-17 | 0.0159 | 0.00149 | | 0.0649 | | 0.318 | |  |
| rs72936846 | 1.47E-17 | 0.0144 | 0.00146 | | 0.0702 | | 0.347 | |  |
| rs72936847 | 1.48E-17 | 0.0145 | 0.00149 | | 0.0721 | | 0.333 | |  |
| rs72936852 | 1.59E-17 | 0.0158 | 0.00146 | | 0.0659 | | 0.319 | |  |
| rs72936856 | 1.58E-17 | 0.0159 | 0.00143 | | 0.0662 | | 0.323 | |  |
| rs6719001 | 4.74E-11 | 0.0158 | 0.00142 | | 0.0661 | | 0.323 | |  |
| rs138805020 | 1.15E-17 | 0.0158 | 0.00141 | | 0.0657 | | 0.323 | |  |
| rs7591653 | 6.22E-18 | 0.0161 | 0.0014 | | 0.0656 | | 0.323 | |  |
| rs7603972 | 7.57E-18 | 0.0162 | 0.00143 | | 0.0659 | | 0.325 | |  |
| rs9749722 | 8.60E-18 | 0.0158 | 0.00135 | | 0.0652 | | 0.329 | |  |
| rs4673240 | 1.45E-12 | 0.0281 | 0.00138 | | 0.0673 | | 0.306 | |  |
| rs72936860 | 8.98E-18 | 0.0165 | 0.00139 | | 0.0697 | | 0.314 | |  |
| rs72936862 | 9.24E-18 | 0.0164 | 0.00138 | | 0.0704 | | 0.313 | |  |
| rs72936866 | 9.26E-18 | 0.0153 | 0.00117 | | 0.0661 | | 0.298 | |  |
| rs114527590 | 8.98E-18 | 0.0156 | 0.00146 | | 0.0718 | | 0.308 | |  |
| rs72936869 | 6.70E-18 | 0.017 | 0.00141 | | 0.0699 | | 0.325 | |  |
| rs72936870 | 9.30E-18 | 0.0164 | 0.00137 | | 0.0702 | | 0.312 | |  |
| rs72936872 | 8.32E-18 | 0.0279 | 0.00136 | | 0.0702 | | 0.306 | |  |
| rs72936873 | 9.37E-18 | 0.0164 | 0.00136 | | 0.0701 | | 0.311 | |  |
| rs72936875 | 9.65E-18 | 0.0144 | 0.00141 | | 0.071 | | 0.304 | |  |
| rs72936879 | 9.56E-18 | 0.0163 | 0.00139 | | 0.0688 | | 0.309 | |  |
| rs72936881 | 1.11E-17 | 0.0172 | 0.00158 | | 0.0719 | | 0.323 | |  |
| rs72936882 | 9.49E-18 | 0.0163 | 0.00143 | | 0.0691 | | 0.309 | |  |
| rs142250318 | 1.16E-17 | 0.0157 | 0.00143 | | 0.0682 | | 0.315 | |  |
| rs114393235 | 9.48E-18 | 0.0161 | 0.00143 | | 0.0692 | | 0.308 | |  |
| rs72926767 | 1.15E-17 | 0.0155 | 0.00149 | | 0.0701 | | 0.301 | |  |
| rs72926769 | 9.56E-18 | 0.0156 | 0.00144 | | 0.0694 | | 0.309 | |  |
| rs72926771 | 1.02E-17 | 0.0156 | 0.00152 | | 0.0661 | | 0.31 | |  |
| rs72926772 | 9.98E-18 | 0.0152 | 0.00146 | | 0.0682 | | 0.307 | |  |
| rs77230711 | 1.06E-17 | 0.015 | 0.00141 | | 0.0696 | | 0.302 | |  |
| rs72926779 | 1.03E-17 | 0.0151 | 0.00144 | | 0.0694 | | 0.305 | |  |
| rs75869289 | 1.03E-17 | 0.015 | 0.00141 | | 0.0673 | | 0.305 | |  |
| rs140244541 | 3.33E-16 | 0.0186 | 0.000219 | | 0.0637 | | 0.065 | |  |
| rs72926781 | 1.03E-17 | 0.0148 | 0.00141 | | 0.0674 | | 0.304 | |  |
| rs72926782 | 1.09E-17 | 0.0146 | 0.0014 | | 0.067 | | 0.301 | |  |
| rs116773016 | 1.57E-17 | 0.0148 | 0.00142 | | 0.0674 | | 0.301 | |  |
| rs114604411 | 1.57E-17 | 0.0148 | 0.00142 | | 0.0674 | | 0.301 | |  |
| rs76298043 | 1.09E-17 | 0.0147 | 0.00143 | | 0.0669 | | 0.303 | |  |
| rs72926783 | 1.09E-17 | 0.0146 | 0.0014 | | 0.0668 | | 0.302 | |  |
| rs72926786 | 1.07E-17 | 0.0145 | 0.00142 | | 0.0666 | | 0.301 | |  |
| rs72926787 | 1.11E-17 | 0.0145 | 0.0014 | | 0.0667 | | 0.302 | |  |
| rs116678869 | 1.12E-17 | 0.0144 | 0.00139 | | 0.0666 | | 0.3 | |  |
| rs114702158 | 1.11E-17 | 0.0155 | 0.00129 | | 0.0688 | | 0.304 | |  |
| rs72926791 | 1.13E-17 | 0.0145 | 0.00138 | | 0.0672 | | 0.3 | |  |
| rs114110842 | 1.13E-17 | 0.0147 | 0.0014 | | 0.0674 | | 0.299 | |  |
| rs114079739 | 1.09E-17 | 0.0146 | 0.00138 | | 0.0673 | | 0.298 | |  |
| rs72926793 | 1.26E-17 | 0.0147 | 0.00144 | | 0.0673 | | 0.304 | |  |
| rs72926794 | 1.09E-17 | 0.0146 | 0.00141 | | 0.067 | | 0.298 | |  |
| rs72926796 | 1.04E-17 | 0.0146 | 0.00141 | | 0.067 | | 0.299 | |  |
| rs72926798 | 8.69E-18 | 0.0149 | 0.00134 | | 0.0683 | | 0.307 | |  |
| rs72926799 | 1.02E-17 | 0.0147 | 0.00137 | | 0.0674 | | 0.299 | |  |
| rs72926800 | 1.09E-17 | 0.0159 | 0.00136 | | 0.0667 | | 0.295 | |  |
| rs115194657 | 1.03E-17 | 0.0148 | 0.00136 | | 0.0674 | | 0.298 | |  |
| rs72926802 | 1.09E-17 | 0.0149 | 0.00137 | | 0.0674 | | 0.298 | |  |
| rs79642273 | 1.10E-17 | 0.0149 | 0.00136 | | 0.0675 | | 0.298 | |  |
| rs114123510 | 4.83E-18 | 0.0154 | 0.00133 | | 0.0674 | | 0.3 | |  |
| rs74421437 | 1.09E-17 | 0.0148 | 0.00135 | | 0.0672 | | 0.3 | |  |
| rs72928605 | 4.44E-16 | 0.0246 | 0.00164 | | 0.0732 | | 0.352 | |  |
| rs149268645 | 1.10E-14 | 0.0264 | 0.00171 | | 0.0713 | | 0.36 | |  |
| rs141002954 | 1.12E-17 | 0.0151 | 0.00134 | | 0.0675 | | 0.298 | |  |
| rs1541853 | 1.04E-17 | 0.0148 | 0.00143 | | 0.0701 | | 0.308 | |  |
| rs72928608 | 1.03E-17 | 0.0139 | 0.00167 | | 0.057 | | 0.289 | |  |
| rs72928609 | 1.09E-17 | 0.0155 | 0.00133 | | 0.0681 | | 0.298 | |  |
| rs72928610 | 1.07E-17 | 0.0158 | 0.00132 | | 0.0679 | | 0.294 | |  |
| rs72928613 | 4.19E-17 | 0.0248 | 0.00112 | | 0.067 | | 0.323 | |  |
| rs143450835 | 6.90E-17 | 0.0167 | 0.0015 | | 0.0661 | | 0.303 | |  |
| rs72928620 | 8.93E-18 | 0.0157 | 0.00131 | | 0.0695 | | 0.302 | |  |
| rs72932553 | 1.37E-17 | 0.0168 | 0.00136 | | 0.0672 | | 0.295 | |  |
| rs7579667 | 1.10E-17 | 0.0165 | 0.00136 | | 0.0697 | | 0.3 | |  |
| rs72932554 | 1.04E-17 | 0.0165 | 0.00134 | | 0.0681 | | 0.3 | |  |
| rs72932556 | 1.22E-17 | 0.0166 | 0.00137 | | 0.0671 | | 0.295 | |  |
| rs72932557 | 1.10E-17 | 0.0163 | 0.00163 | | 0.0649 | | 0.267 | |  |
| rs72932558 | 1.11E-17 | 0.0168 | 0.00137 | | 0.0668 | | 0.298 | |  |
| rs72932559 | 1.23E-17 | 0.0163 | 0.00126 | | 0.0679 | | 0.307 | |  |
| rs72932560 | 1.09E-17 | 0.0168 | 0.00133 | | 0.0671 | | 0.299 | |  |
| rs72932561 | 1.09E-17 | 0.0169 | 0.00134 | | 0.0679 | | 0.298 | |  |
| rs76890136 | 1.10E-17 | 0.0168 | 0.00143 | | 0.0669 | | 0.302 | |  |
| rs72932566 | 8.58E-18 | 0.0159 | 0.00131 | | 0.0672 | | 0.305 | |  |
| rs72932572 | 1.06E-17 | 0.016 | 0.0015 | | 0.0665 | | 0.295 | |  |
| rs72932573 | 1.11E-17 | 0.0158 | 0.00143 | | 0.0684 | | 0.295 | |  |
| rs148707292 | 1.08E-17 | 0.0159 | 0.00143 | | 0.0684 | | 0.295 | |  |
| rs145299755 | 9.13E-18 | 0.0166 | 0.00141 | | 0.068 | | 0.293 | |  |
| rs140750546 | 8.52E-18 | 0.0297 | 0.00176 | | 0.0824 | | 0.418 | |  |
| rs76461893 | 7.19E-18 | 0.0147 | 0.0014 | | 0.0727 | | 0.29 | |  |
| rs72932574 | 6.84E-18 | 0.0157 | 0.00151 | | 0.0691 | | 0.298 | |  |
| rs72932575 | 6.44E-18 | 0.0161 | 0.00157 | | 0.0687 | | 0.294 | |  |
| rs72932583 | 5.25E-18 | 0.0162 | 0.00154 | | 0.0696 | | 0.299 | |  |
| rs6728861 | 4.97E-18 | 0.0164 | 0.00155 | | 0.0698 | | 0.301 | |  |
| rs74923585 | 1.45E-09 | 0.0546 | 0.0166 | | 0.51 | | 0.443 | |  |
| rs148812085 | 4.38E-18 | 0.0168 | 0.00169 | | 0.0759 | | 0.294 | |  |
| rs4675310 | 7.99E-15 | 0.0274 | 0.00166 | | 0.0746 | | 0.295 | |  |
| rs2351524 | 8.88E-15 | 0.0273 | 0.00175 | | 0.0755 | | 0.283 | |  |
| rs72932588 | 3.52E-18 | 0.0169 | 0.00224 | | 0.0703 | | 0.298 | |  |
| rs72932590 | 3.93E-18 | 0.0167 | 0.00222 | | 0.0708 | | 0.299 | |  |
| rs145538381 | 4.05E-18 | 0.0153 | 0.00216 | | 0.0736 | | 0.298 | |  |
| rs115654617 | 3.12E-18 | 0.015 | 0.00216 | | 0.0732 | | 0.297 | |  |
| rs140168762 | 3.83E-18 | 0.0148 | 0.00208 | | 0.075 | | 0.299 | |  |
| rs72934505 | 5.82E-18 | 0.0134 | 0.00212 | | 0.0882 | | 0.292 | |  |
| rs72934510 | 4.94E-18 | 0.0135 | 0.00216 | | 0.0835 | | 0.291 | |  |
| rs72934512 | 4.85E-18 | 0.0136 | 0.0023 | | 0.0792 | | 0.286 | |  |
| rs72934513 | 3.88E-18 | 0.0131 | 0.00235 | | 0.081 | | 0.274 | |  |
| rs151316549 | 6.55E-18 | 0.0245 | 0.00245 | | 0.0804 | | 0.261 | |  |
| rs78907692 | 6.57E-18 | 0.0143 | 0.00224 | | 0.0811 | | 0.283 | |  |
| rs72934514 | 1.11E-16 | 0.0258 | 0.00302 | | 0.0734 | | 0.357 | |  |
| rs72934518 | 7.21E-18 | 0.0137 | 0.00252 | | 0.0824 | | 0.287 | |  |
| rs72934519 | 8.02E-18 | 0.0136 | 0.00273 | | 0.0892 | | 0.295 | |  |
| rs115810193 | 8.06E-18 | 0.0129 | 0.0025 | | 0.086 | | 0.305 | |  |
| rs139333388 | 1.25E-17 | 0.0123 | 0.00249 | | 0.087 | | 0.303 | |  |
| rs142013255 | 1.15E-17 | 0.0123 | 0.00235 | | 0.0891 | | 0.297 | |  |
| rs139621023 | 3.06E-09 | 0.0501 | 0.0103 | | 0.692 | | 0.463 | |  |
| rs72934535 | 1.27E-17 | 0.00228 | 0.00321 | | 0.435 | | 0.321 | |  |
| rs72934537 | 1.18E-17 | 0.0117 | 0.00239 | | 0.0862 | | 0.294 | |  |
| rs72934545 | 1.21E-17 | 0.0129 | 0.00216 | | 0.088 | | 0.3 | |  |
| rs72934546 | 1.29E-17 | 0.0115 | 0.00228 | | 0.0862 | | 0.294 | |  |
| rs72934550 | 1.68E-17 | 0.0228 | 0.00216 | | 0.0882 | | 0.275 | |  |
| rs72934551 | 1.69E-17 | 0.0118 | 0.00234 | | 0.0885 | | 0.295 | |  |
| rs72934554 | 3.22E-17 | 0.0119 | 0.00221 | | 0.0882 | | 0.295 | |  |
| rs72934556 | 1.33E-15 | 0.0226 | 0.00287 | | 0.0824 | | 0.378 | |  |
| rs149215222 | 2.29E-17 | 0.0129 | 0.00228 | | 0.088 | | 0.308 | |  |
| rs72934563 | 2.18E-17 | 0.0126 | 0.00246 | | 0.0856 | | 0.302 | |  |
| rs72934573 | 1.96E-17 | 0.0157 | 0.00206 | | 0.0996 | | 0.293 | |  |
| rs72934583 | 2.27E-17 | 0.0147 | 0.00229 | | 0.0988 | | 0.294 | |  |
| rs140201293 | 1.11E-16 | 0.0266 | 0.00322 | | 0.081 | | 0.394 | |  |
| rs72934589 | 2.42E-17 | 0.0177 | 0.00294 | | 0.0786 | | 0.328 | |  |
| rs72934591 | 2.44E-17 | 0.0183 | 0.00281 | | 0.078 | | 0.327 | |  |
| rs146099848 | 2.22E-16 | 0.0336 | 0.00384 | | 0.0965 | | 0.386 | |  |
| rs72934601 | 2.22E-16 | 0.037 | 0.0038 | | 0.089 | | 0.403 | |  |
| rs72936304 | 2.22E-16 | 0.0382 | 0.00374 | | 0.0963 | | 0.389 | |  |
| rs72936309 | 4.44E-16 | 0.0369 | 0.00528 | | 0.0916 | | 0.401 | |  |
| rs10932008 | 2.91E-10 | 0.00412 | 0.00348 | | 0.0591 | | 0.0745 | |  |
| rs72936323 | 4.44E-16 | 0.0417 | 0.00337 | | 0.0921 | | 0.378 | |  |
| rs72936326 | 4.44E-16 | 0.0423 | 0.00388 | | 0.0857 | | 0.383 | |  |
| rs72936332 | 4.44E-16 | 0.0422 | 0.00419 | | 0.0846 | | 0.385 | |  |
| rs72936348 | 4.44E-16 | 0.0387 | 0.00403 | | 0.0856 | | 0.387 | |  |
| rs72936353 | 4.44E-16 | 0.03 | 0.00316 | | 0.0831 | | 0.383 | |  |
| rs116219813 | 1.22E-15 | 0.0306 | 0.00285 | | 0.0962 | | 0.379 | |  |
| rs72938315 | 5.36E-12 | 0.0527 | 0.00384 | | 0.128 | | 0.865 | |  |
| rs115600411 | 3.86E-13 | 0.0154 | 0.00681 | | 0.129 | | 0.483 | |  |
| rs72938351 | 1.85E-14 | 0.00305 | 0.00176 | | 0.615 | | 0.31 | |  |
| rs114407963 | 6.77E-14 | 0.0117 | 0.0104 | | 0.123 | | 0.656 | |  |
| rs114863726 | 5.64E-14 | 0.0119 | 0.00917 | | 0.125 | | 0.651 | |  |
| rs116443099 | 2.91E-08 | 0.0846 | 0.0152 | | 0.549 | | 0.706 | |  |
| rs4484022 | 6.31E-09 | 0.692 | 0.391 | | 0.524 | | 0.333 | |  |
| rs116826171 | 1.05E-08 | 0.527 | 0.0879 | | 0.949 | | 0.571 | |  |
| rs4675332 | 4.58E-09 | 0.698 | 0.424 | | 0.497 | | 0.344 | |  |
| rs116426890 | 2.95E-13 | 0.0124 | 0.0151 | | 0.16 | | 0.638 | |  |
| rs12474416 | 4.63E-10 | 0.759 | 0.249 | | 0.959 | | 0.173 | |  |
| rs12466364 | 5.32E-10 | 0.739 | 0.264 | | 0.946 | | 0.187 | |  |
| rs35071165 | 5.15E-10 | 0.742 | 0.234 | | 0.977 | | 0.16 | |  |
| rs10197623 | 4.41E-10 | 0.785 | 0.243 | | 0.976 | | 0.172 | |  |
| rs12693989 | 3.36E-10 | 0.798 | 0.277 | | 0.904 | | 0.219 | |  |
| rs113217304 | 6.06E-10 | 0.797 | 0.261 | | 0.887 | | 0.211 | |  |
| rs13416670 | 3.87E-10 | 0.782 | 0.278 | | 0.89 | | 0.221 | |  |
| rs78999781 | 1.88E-08 | 0.0935 | 0.162 | | 0.668 | | 0.746 | |  |
| rs77137125 | 8.40E-09 | 0.163 | 0.051 | | 0.945 | | 0.524 | |  |
| rs139644567 | 6.91E-08 | 0.0163 | 0.0129 | | 0.826 | | 0.575 | |  |
| rs140274075 | 4.78E-08 | 0.158 | 0.0489 | | 0.885 | | 0.495 | |  |
| rs10174652 | 4.66E-08 | 0.231 | 0.134 | | 0.718 | | 0.943 | |  |
| rs78617107 | 5.88E-08 | 0.294 | 0.132 | | 0.564 | | 0.987 | |  |
| rs13324341 | 4.27E-09 | 0.00936 | 0.568 | | 0.923 | | 0.0951 | |  |
| rs1199338 | 3.90E-09 | 0.0161 | 0.521 | | 0.986 | | 0.0683 | |  |
| rs1199337 | 4.14E-09 | 0.0147 | 0.515 | | 0.959 | | 0.0653 | |  |
| rs185244 | 4.09E-09 | 0.0202 | 0.443 | | 0.781 | | 0.0894 | |  |
| rs1092338 | 5.39E-08 | 0.0638 | 0.396 | | 0.898 | | 0.273 | |  |
| rs1678440 | 5.54E-08 | 0.064 | 0.397 | | 0.891 | | 0.272 | |  |
| rs1720819 | 5.06E-08 | 0.0632 | 0.396 | | 0.895 | | 0.275 | |  |
| rs1199330 | 6.13E-08 | 0.0624 | 0.396 | | 0.893 | | 0.273 | |  |
| rs17081933 | 8.63E-08 | 0.972 | 0.584 | | 0.901 | | 0.577 | |  |
| rs56155140 | 8.81E-08 | 0.857 | 0.62 | | 0.867 | | 0.563 | |  |
| rs7687767 | 8.43E-08 | 0.852 | 0.61 | | 0.895 | | 0.576 | |  |
| rs6554401 | 6.94E-08 | 0.729 | 0.671 | | 0.717 | | 0.77 | |  |
| rs72627508 | 9.85E-08 | 0.982 | 0.714 | | 0.865 | | 0.606 | |  |
| rs17087335 | 4.59E-08 | 0.971 | 0.707 | | 0.886 | | 0.584 | |  |
| rs72627509 | 7.04E-08 | 0.794 | 0.697 | | 0.791 | | 0.783 | |  |
| rs57265257 | 5.30E-08 | 0.907 | 0.765 | | 0.846 | | 0.705 | |  |
| rs7668383 | 3.13E-09 | 0.17 | 0.669 | | 0.0257 | | 0.556 | |  |
| rs2059904 | 2.83E-09 | 0.165 | 0.427 | | 0.0914 | | 0.706 | |  |
| rs4593108 | 8.82E-10 | 0.159 | 0.381 | | 0.0886 | | 0.735 | |  |
| rs1429137 | 5.80E-09 | 0.163 | 0.376 | | 0.09 | | 0.743 | |  |
| rs7667633 | 5.64E-09 | 0.157 | 0.363 | | 0.0998 | | 0.772 | |  |
| rs17023141 | 6.48E-09 | 0.172 | 0.356 | | 0.0914 | | 0.769 | |  |
| rs6836670 | 9.00E-09 | 0.177 | 0.348 | | 0.0946 | | 0.772 | |  |
| rs6812819 | 2.35E-08 | 0.178 | 0.363 | | 0.0951 | | 0.851 | |  |
| rs9654228 | 1.73E-08 | 0.185 | 0.364 | | 0.0914 | | 0.834 | |  |
| rs1429141 | 1.83E-08 | 0.193 | 0.353 | | 0.0897 | | 0.837 | |  |
| rs28645698 | 1.86E-08 | 0.182 | 0.344 | | 0.0881 | | 0.851 | |  |
| rs1429142 | 3.60E-08 | 0.197 | 0.359 | | 0.0815 | | 0.81 | |  |
| rs6810798 | 2.51E-08 | 0.206 | 0.337 | | 0.0733 | | 0.929 | |  |
| rs28720373 | 5.31E-08 | 0.168 | 0.366 | | 0.0954 | | 0.824 | |  |
| rs28623525 | 5.23E-08 | 0.166 | 0.37 | | 0.0942 | | 0.832 | |  |
| rs7688323 | 7.68E-09 | 0.278 | 0.321 | | 0.173 | | 0.508 | |  |
| rs2306557 | 8.85E-08 | 0.602 | 0.434 | | 0.712 | | 0.786 | |  |
| rs13143871 | 7.77E-08 | 0.595 | 0.438 | | 0.739 | | 0.724 | |  |
| rs72685791 | 8.02E-09 | 0.38 | 0.416 | | 0.159 | | 0.571 | |  |
| rs34914832 | 9.91E-09 | 0.275 | 0.401 | | 0.22 | | 0.542 | |  |
| rs72687508 | 9.69E-09 | 0.264 | 0.461 | | 0.233 | | 0.554 | |  |
| rs3796592 | 7.80E-09 | 0.265 | 0.49 | | 0.269 | | 0.546 | |  |
| rs56329057 | 1.26E-08 | 0.511 | 0.538 | | 0.364 | | 0.703 | |  |
| rs2242296 | 5.78E-08 | 0.586 | 0.214 | | 0.486 | | 0.739 | |  |
| rs7692387 | 7.35E-09 | 0.423 | 0.504 | | 0.422 | | 0.705 | |  |
| rs3796587 | 6.16E-09 | 0.471 | 0.497 | | 0.439 | | 0.722 | |  |
| rs3796586 | 4.59E-08 | 0.665 | 0.228 | | 0.51 | | 0.744 | |  |
| rs2306556 | 9.74E-09 | 0.474 | 0.481 | | 0.444 | | 0.728 | |  |
| rs6827908 | 5.24E-08 | 0.66 | 0.234 | | 0.498 | | 0.725 | |  |
| rs3796585 | 4.98E-08 | 0.663 | 0.237 | | 0.503 | | 0.74 | |  |
| rs12643599 | 7.05E-08 | 0.684 | 0.233 | | 0.592 | | 0.695 | |  |
| rs72689147 | 6.07E-09 | 0.55 | 0.49 | | 0.584 | | 0.69 | |  |
| rs3796584 | 6.31E-08 | 0.726 | 0.238 | | 0.611 | | 0.749 | |  |
| rs6812618 | 7.74E-08 | 0.794 | 0.259 | | 0.556 | | 0.703 | |  |
| rs6838927 | 8.19E-08 | 0.809 | 0.258 | | 0.556 | | 0.735 | |  |
| rs6838956 | 8.31E-08 | 0.815 | 0.259 | | 0.568 | | 0.734 | |  |
| rs3796582 | 9.62E-08 | 0.794 | 0.259 | | 0.564 | | 0.729 | |  |
| rs3796581 | 1.36E-08 | 0.59 | 0.496 | | 0.648 | | 0.648 | |  |
| rs2306555 | 9.22E-08 | 0.811 | 0.257 | | 0.565 | | 0.769 | |  |
| rs67168236 | 9.66E-08 | 0.8 | 0.239 | | 0.561 | | 0.809 | |  |
| rs55673113 | 8.90E-08 | 0.807 | 0.236 | | 0.554 | | 0.833 | |  |
| rs11724647 | 5.40E-08 | 0.687 | 0.459 | | 0.538 | | 0.599 | |  |
| rs11721947 | 9.07E-08 | 0.582 | 0.536 | | 0.424 | | 0.551 | |  |
| rs2327616 | 7.04E-08 | 0.178 | 0.0409 | | 0.116 | | 0.669 | |  |
| rs13217579 | 5.59E-08 | 0.19 | 0.0508 | | 0.148 | | 0.592 | |  |
| rs6916397 | 3.10E-09 | 0.233 | 0.12 | | 0.0113 | | 0.492 | |  |
| rs6916418 | 3.26E-09 | 0.247 | 0.137 | | 0.0139 | | 0.405 | |  |
| rs6916421 | 2.94E-09 | 0.26 | 0.146 | | 0.0118 | | 0.431 | |  |
| rs12202891 | 9.65E-09 | 0.00504 | 0.961 | | 0.357 | | 0.909 | |  |
| rs1572000 | 3.22E-09 | 0.229 | 0.139 | | 0.0236 | | 0.437 | |  |
| rs1412749 | 2.76E-09 | 0.209 | 0.147 | | 0.0181 | | 0.462 | |  |
| rs6941422 | 4.95E-09 | 0.214 | 0.136 | | 0.0178 | | 0.444 | |  |
| rs7757205 | 6.48E-09 | 0.229 | 0.152 | | 0.0151 | | 0.51 | |  |
| rs9463106 | 4.64E-09 | 0.204 | 0.155 | | 0.0159 | | 0.511 | |  |
| rs6922969 | 4.20E-09 | 0.2 | 0.163 | | 0.0159 | | 0.514 | |  |
| rs6938417 | 3.99E-09 | 0.212 | 0.153 | | 0.015 | | 0.517 | |  |
| rs1572002 | 5.20E-09 | 0.211 | 0.156 | | 0.0154 | | 0.511 | |  |
| rs9463110 | 4.54E-09 | 0.209 | 0.16 | | 0.0161 | | 0.528 | |  |
| rs9472551 | 4.89E-09 | 0.21 | 0.157 | | 0.0149 | | 0.506 | |  |
| rs1412751 | 4.23E-09 | 0.213 | 0.169 | | 0.0167 | | 0.523 | |  |
| rs944766 | 4.02E-09 | 0.126 | 0.149 | | 0.0421 | | 0.522 | |  |
| rs2015764 | 4.67E-09 | 0.208 | 0.155 | | 0.0163 | | 0.529 | |  |
| rs6458460 | 4.29E-09 | 0.206 | 0.153 | | 0.0167 | | 0.531 | |  |
| rs6458461 | 4.31E-09 | 0.207 | 0.154 | | 0.0152 | | 0.523 | |  |
| rs4714866 | 4.30E-09 | 0.205 | 0.151 | | 0.014 | | 0.494 | |  |
| rs4714868 | 4.31E-09 | 0.205 | 0.151 | | 0.0138 | | 0.494 | |  |
| rs10807323 | 1.01E-14 | 0.111 | 0.294 | | 0.00244 | | 0.608 | |  |
| rs6924150 | 4.38E-09 | 0.192 | 0.141 | | 0.0148 | | 0.553 | |  |
| rs7757858 | 5.42E-09 | 0.217 | 0.153 | | 0.0138 | | 0.522 | |  |
| rs17617207 | 3.84E-09 | 0.218 | 0.152 | | 0.0145 | | 0.531 | |  |
| rs6917492 | 6.16E-08 | 0.161 | 0.0495 | | 0.125 | | 0.752 | |  |
| rs6917493 | 5.71E-08 | 0.16 | 0.0493 | | 0.123 | | 0.758 | |  |
| rs9381401 | 1.55E-15 | 0.143 | 0.222 | | 0.00314 | | 0.51 | |  |
| rs6919327 | 3.88E-09 | 0.206 | 0.146 | | 0.0142 | | 0.521 | |  |
| rs7762547 | 3.47E-09 | 0.22 | 0.14 | | 0.0152 | | 0.489 | |  |
| rs7764490 | 8.11E-09 | 0.21 | 0.181 | | 0.0149 | | 0.573 | |  |
| rs1412740 | 5.09E-09 | 0.199 | 0.218 | | 0.0146 | | 0.564 | |  |
| rs1412741 | 1.48E-08 | 0.5 | 0.114 | | 0.0476 | | 0.725 | |  |
| rs4711841 | 1.52E-08 | 0.513 | 0.119 | | 0.046 | | 0.698 | |  |
| rs1332840 | 8.48E-09 | 0.211 | 0.182 | | 0.0136 | | 0.582 | |  |
| rs7776079 | 4.97E-09 | 0.195 | 0.171 | | 0.0118 | | 0.607 | |  |
| rs2026458 | 1.26E-12 | 0.223 | 0.491 | | 0.0115 | | 0.521 | |  |
| rs12215208 | 5.68E-14 | 0.252 | 0.387 | | 0.0229 | | 0.463 | |  |
| rs4714930 | 2.61E-08 | 0.0727 | 0.296 | | 0.102 | | 0.712 | |  |
| rs1360579 | 8.75E-08 | 0.116 | 0.0337 | | 0.766 | | 0.877 | |  |
| rs9472777 | 4.75E-08 | 0.149 | 0.031 | | 0.831 | | 0.992 | |  |
| rs4714946 | 4.19E-08 | 0.148 | 0.0367 | | 0.775 | | 0.959 | |  |
| rs9381462 | 2.47E-11 | 0.041 | 0.0112 | | 0.336 | | 0.78 | |  |
| rs1571997 | 2.59E-10 | 0.0349 | 0.00982 | | 0.29 | | 0.832 | |  |
| rs66867947 | 9.58E-08 | 0.164 | 0.0482 | | 0.876 | | 0.963 | |  |
| rs1332841 | 9.22E-08 | 0.134 | 0.0383 | | 0.819 | | 0.948 | |  |
| rs9472786 | 4.31E-08 | 0.129 | 0.0354 | | 0.745 | | 0.912 | |  |
| rs9472790 | 1.68E-10 | 0.0355 | 0.00935 | | 0.307 | | 0.793 | |  |
| rs17679501 | 2.19E-08 | 0.134 | 0.0357 | | 0.787 | | 0.94 | |  |
| rs4711857 | 1.45E-08 | 0.128 | 0.0376 | | 0.719 | | 0.942 | |  |
| rs4711858 | 1.23E-08 | 0.131 | 0.0425 | | 0.667 | | 0.884 | |  |
| rs8180628 | 2.97E-09 | 0.0849 | 0.129 | | 0.768 | | 0.942 | |  |
| rs1537340 | 5.60E-17 | 0.0315 | 0.0572 | | 0.702 | | 0.664 | |  |
| rs13208248 | 1.33E-17 | 0.0493 | 0.307 | | 0.652 | | 0.793 | |  |
| rs71562459 | 1.11E-16 | 0.0606 | 0.0823 | | 0.783 | | 0.938 | |  |
| rs1332844 | 1.11E-15 | 0.0626 | 0.734 | | 0.579 | | 0.949 | |  |
| rs34944538 | 1.11E-16 | 0.0655 | 0.0841 | | 0.79 | | 0.938 | |  |
| rs35355695 | 1.11E-16 | 0.0723 | 0.103 | | 0.751 | | 0.946 | |  |
| rs7750679 | 1.55E-15 | 0.0637 | 0.787 | | 0.609 | | 0.904 | |  |
| rs1332845 | 2.22E-16 | 0.0293 | 0.0772 | | 0.742 | | 0.82 | |  |
| rs6925904 | 1.11E-15 | 0.0574 | 0.754 | | 0.64 | | 0.937 | |  |
| rs1332846 | 2.22E-16 | 0.0305 | 0.071 | | 0.73 | | 0.834 | |  |
| rs1332847 | 2.22E-16 | 0.0325 | 0.0707 | | 0.747 | | 0.826 | |  |
| rs2876300 | 3.33E-16 | 0.0316 | 0.074 | | 0.751 | | 0.831 | |  |
| rs9296512 | 2.66E-15 | 0.0514 | 0.787 | | 0.614 | | 0.925 | |  |
| rs62389954 | 3.33E-16 | 0.0337 | 0.0712 | | 0.692 | | 0.835 | |  |
| rs4714951 | 3.33E-16 | 0.0336 | 0.0722 | | 0.687 | | 0.829 | |  |
| rs13219256 | 2.22E-16 | 0.0318 | 0.0728 | | 0.716 | | 0.835 | |  |
| rs9395214 | 4.44E-16 | 0.0534 | 0.822 | | 0.517 | | 0.953 | |  |
| rs7751826 | 2.17E-18 | 0.0498 | 0.868 | | 0.527 | | 0.947 | |  |
| rs9369640 | 7.78E-19 | 0.0567 | 0.861 | | 0.585 | | 0.951 | |  |
| rs62389955 | 8.39E-21 | 0.0836 | 0.402 | | 0.891 | | 0.737 | |  |
| rs6911226 | 3.10E-18 | 0.0358 | 0.841 | | 0.539 | | 0.979 | |  |
| rs6915983 | 3.27E-18 | 0.0356 | 0.861 | | 0.543 | | 0.978 | |  |
| rs4714955 | 2.51E-21 | 0.0785 | 0.393 | | 0.885 | | 0.793 | |  |
| rs9349379 | 1.81E-42 | 0.158 | 0.0543 | | 0.32 | | 0.827 | |  |
| rs2876301 | 1.47E-17 | 0.0271 | 0.73 | | 0.466 | | 0.892 | |  |
| rs7760016 | 6.57E-17 | 0.0484 | 0.834 | | 0.565 | | 0.985 | |  |
| rs7760527 | 5.21E-17 | 0.0484 | 0.845 | | 0.577 | | 0.988 | |  |
| rs13197912 | 1.80E-20 | 0.0795 | 0.376 | | 0.858 | | 0.791 | |  |
| rs7454157 | 3.31E-20 | 0.0454 | 0.842 | | 0.631 | | 0.961 | |  |
| rs12530250 | 1.52E-20 | 0.0802 | 0.366 | | 0.853 | | 0.77 | |  |
| rs9381494 | 8.26E-17 | 0.0466 | 0.825 | | 0.576 | | 0.999 | |  |
| rs4711863 | 1.62E-21 | 0.0849 | 0.428 | | 0.631 | | 0.632 | |  |
| rs2876303 | 7.41E-18 | 0.0532 | 0.846 | | 0.793 | | 0.851 | |  |
| rs8180558 | 6.12E-18 | 0.0869 | 0.399 | | 0.621 | | 0.651 | |  |
| rs111395058 | 6.42E-21 | 0.0127 | 0.726 | | 0.895 | | 0.659 | |  |
| rs9395224 | 1.22E-12 | 0.00564 | 0.378 | | 0.833 | | 0.614 | |  |
| rs9381500 | 1.14E-17 | 0.0838 | 0.437 | | 0.627 | | 0.658 | |  |
| rs9369650 | 1.05E-17 | 0.0505 | 0.874 | | 0.802 | | 0.891 | |  |
| rs2327621 | 3.04E-17 | 0.0835 | 0.439 | | 0.64 | | 0.666 | |  |
| rs34343839 | 1.32E-20 | 0.0795 | 0.447 | | 0.665 | | 0.666 | |  |
| rs1014342 | 1.17E-17 | 0.0509 | 0.867 | | 0.788 | | 0.886 | |  |
| rs62386818 | 8.38E-21 | 0.0791 | 0.441 | | 0.68 | | 0.672 | |  |
| rs9369652 | 9.36E-18 | 0.0504 | 0.864 | | 0.805 | | 0.881 | |  |
| rs1953088 | 8.67E-18 | 0.0534 | 0.831 | | 0.825 | | 0.865 | |  |
| rs12526453 | 2.14E-20 | 0.0537 | 0.353 | | 0.684 | | 0.555 | |  |
| rs4714990 | 2.67E-17 | 0.0512 | 0.88 | | 0.698 | | 0.756 | |  |
| rs6458545 | 3.50E-17 | 0.0418 | 0.736 | | 0.724 | | 0.854 | |  |
| rs7739181 | 2.64E-20 | 0.0599 | 0.363 | | 0.548 | | 0.659 | |  |
| rs13209002 | 7.06E-11 | 0.679 | 0.00252 | | 0.399 | | 0.925 | |  |
| rs13201878 | 1.11E-16 | 0.0866 | 0.0674 | | 0.283 | | 0.99 | |  |
| rs4715000 | 1.11E-15 | 0.103 | 0.104 | | 0.357 | | 0.971 | |  |
| rs6905419 | 3.15E-14 | 0.114 | 0.11 | | 0.347 | | 0.979 | |  |
| rs7748390 | 1.78E-15 | 0.112 | 0.112 | | 0.348 | | 0.992 | |  |
| rs6922782 | 2.69E-14 | 0.121 | 0.101 | | 0.323 | | 0.983 | |  |
| rs7775710 | 6.96E-08 | 0.732 | 0.405 | | 0.278 | | 0.623 | |  |
| rs6906017 | 5.29E-08 | 0.746 | 0.408 | | 0.325 | | 0.603 | |  |
| rs34175679 | 4.62E-09 | 0.791 | 0.521 | | 0.149 | | 0.592 | |  |
| rs36049381 | 2.87E-09 | 0.789 | 0.522 | | 0.147 | | 0.592 | |  |
| rs62386820 | 5.48E-09 | 0.786 | 0.536 | | 0.144 | | 0.594 | |  |
| rs4588689 | 4.23E-09 | 0.776 | 0.569 | | 0.151 | | 0.591 | |  |
| rs62386821 | 8.54E-09 | 0.792 | 0.51 | | 0.147 | | 0.587 | |  |
| rs9473086 | 1.26E-08 | 0.7 | 0.486 | | 0.129 | | 0.543 | |  |
| rs66820937 | 1.07E-08 | 0.728 | 0.484 | | 0.132 | | 0.56 | |  |
| rs11756003 | 1.70E-08 | 0.765 | 0.483 | | 0.141 | | 0.572 | |  |
| rs1544935 | 8.86E-08 | 0.0438 | 0.689 | | 0.948 | | 0.616 | |  |
| rs2894421 | 7.75E-08 | 0.0633 | 0.689 | | 0.922 | | 0.634 | |  |
| rs56336142 | 1.85E-08 | 0.125 | 0.506 | | 0.904 | | 0.919 | |  |
| rs12214118 | 7.13E-08 | 0.0971 | 0.547 | | 0.846 | | 0.865 | |  |
| rs755852 | 6.45E-08 | 0.0858 | 0.634 | | 0.803 | | 0.857 | |  |
| rs1155347 | 5.16E-08 | 0.00974 | 0.49 | | 0.744 | | 0.76 | |  |
| rs12211281 | 5.81E-08 | 0.033 | 0.327 | | 0.956 | | 0.646 | |  |
| rs55856036 | 4.11E-08 | 0.0379 | 0.318 | | 0.917 | | 0.687 | |  |
| rs9394577 | 5.25E-08 | 0.0311 | 0.251 | | 0.95 | | 0.749 | |  |
| rs55902013 | 5.86E-08 | 0.0305 | 0.254 | | 0.899 | | 0.753 | |  |
| rs10456468 | 4.17E-08 | 0.0272 | 0.251 | | 0.892 | | 0.731 | |  |
| rs56015508 | 5.45E-08 | 0.028 | 0.29 | | 0.825 | | 0.733 | |  |
| rs10947786 | 5.61E-08 | 0.0598 | 0.398 | | 0.884 | | 0.794 | |  |
| rs4895388 | 3.80E-10 | 0.2 | 0.376 | | 0.627 | | 0.142 | |  |
| rs12193973 | 8.65E-11 | 0.244 | 0.418 | | 0.712 | | 0.149 | |  |
| rs6906375 | 1.04E-09 | 0.186 | 0.378 | | 0.643 | | 0.143 | |  |
| rs2876224 | 8.79E-10 | 0.195 | 0.375 | | 0.645 | | 0.147 | |  |
| rs11154750 | 1.68E-10 | 0.246 | 0.383 | | 0.789 | | 0.147 | |  |
| rs4895389 | 6.05E-10 | 0.265 | 0.445 | | 0.752 | | 0.11 | |  |
| rs1969783 | 1.10E-10 | 0.23 | 0.416 | | 0.739 | | 0.149 | |  |
| rs1966248 | 3.47E-10 | 0.217 | 0.419 | | 0.696 | | 0.143 | |  |
| rs9285476 | 4.22E-11 | 0.217 | 0.395 | | 0.675 | | 0.165 | |  |
| rs1535616 | 3.65E-11 | 0.109 | 0.532 | | 0.541 | | 0.204 | |  |
| rs7752775 | 3.51E-11 | 0.111 | 0.533 | | 0.642 | | 0.233 | |  |
| rs12202017 | 1.98E-11 | 0.108 | 0.533 | | 0.567 | | 0.191 | |  |
| rs34523729 | 5.32E-11 | 0.143 | 0.623 | | 0.56 | | 0.207 | |  |
| rs2105092 | 5.16E-11 | 0.171 | 0.679 | | 0.547 | | 0.201 | |  |
| rs1832181 | 1.11E-10 | 0.17 | 0.624 | | 0.581 | | 0.206 | |  |
| rs10457618 | 4.81E-11 | 0.145 | 0.6 | | 0.566 | | 0.211 | |  |
| rs12207772 | 4.91E-11 | 0.156 | 0.599 | | 0.565 | | 0.209 | |  |
| rs13218281 | 8.87E-11 | 0.182 | 0.633 | | 0.579 | | 0.213 | |  |
| rs17062991 | 8.86E-11 | 0.184 | 0.621 | | 0.579 | | 0.206 | |  |
| rs12192720 | 4.03E-11 | 0.168 | 0.673 | | 0.57 | | 0.199 | |  |
| rs7769943 | 9.08E-11 | 0.157 | 0.687 | | 0.669 | | 0.252 | |  |
| rs7769954 | 4.93E-11 | 0.161 | 0.713 | | 0.662 | | 0.247 | |  |
| rs12524865 | 5.11E-11 | 0.157 | 0.723 | | 0.68 | | 0.215 | |  |
| rs1967917 | 8.72E-11 | 0.163 | 0.672 | | 0.793 | | 0.266 | |  |
| rs2327426 | 2.16E-11 | 0.233 | 0.68 | | 0.501 | | 0.244 | |  |
| rs12194592 | 2.13E-11 | 0.224 | 0.748 | | 0.57 | | 0.215 | |  |
| rs6569912 | 1.01E-08 | 0.142 | 0.498 | | 0.422 | | 0.301 | |  |
| rs6569913 | 6.26E-09 | 0.149 | 0.441 | | 0.366 | | 0.275 | |  |
| rs2327429 | 6.78E-10 | 0.368 | 0.481 | | 0.699 | | 0.339 | |  |
| rs2327433 | 6.52E-08 | 0.744 | 0.666 | | 0.686 | | 0.629 | |  |
| rs162185 | 1.92E-08 | 0.657 | 0.65 | | 0.736 | | 0.639 | |  |
| rs9457761 | 1.07E-10 | 0.301 | 0.592 | | 0.688 | | 0.742 | |  |
| rs144078421 | 4.97E-10 | 0.23 | 0.73 | | 0.391 | | 0.992 | |  |
| rs4709408 | 9.69E-08 | 0.834 | 0.694 | | 0.516 | | 0.333 | |  |
| rs9457861 | 4.82E-08 | 0.715 | 0.815 | | 0.528 | | 0.464 | |  |
| rs624249 | 6.82E-09 | 0.572 | 0.164 | | 0.182 | | 0.0536 | |  |
| rs10080815 | 1.33E-15 | 0.487 | 0.962 | | 0.278 | | 0.893 | |  |
| rs3127587 | 8.02E-09 | 0.0931 | 0.234 | | 0.471 | | 0.906 | |  |
| rs3127586 | 8.25E-09 | 0.0918 | 0.234 | | 0.487 | | 0.918 | |  |
| rs3125060 | 3.34E-09 | 0.104 | 0.254 | | 0.5 | | 0.815 | |  |
| rs12193418 | 1.13E-08 | 0.0992 | 0.265 | | 0.415 | | 0.895 | |  |
| rs12206585 | 7.63E-09 | 0.0994 | 0.267 | | 0.459 | | 0.855 | |  |
| rs12203303 | 8.14E-09 | 0.0926 | 0.27 | | 0.458 | | 0.802 | |  |
| rs12194962 | 8.14E-09 | 0.0979 | 0.261 | | 0.474 | | 0.804 | |  |
| rs11755806 | 8.42E-09 | 0.0802 | 0.278 | | 0.456 | | 0.802 | |  |
| rs78801493 | 8.14E-09 | 0.105 | 0.251 | | 0.495 | | 0.837 | |  |
| rs77410834 | 8.73E-09 | 0.103 | 0.251 | | 0.501 | | 0.846 | |  |
| rs2183470 | 7.80E-09 | 0.103 | 0.25 | | 0.501 | | 0.835 | |  |
| rs3103351 | 2.24E-08 | 0.0817 | 0.185 | | 0.635 | | 0.774 | |  |
| rs3127581 | 6.83E-09 | 0.105 | 0.253 | | 0.488 | | 0.84 | |  |
| rs3106163 | 3.17E-09 | 0.113 | 0.245 | | 0.486 | | 0.831 | |  |
| rs3120151 | 2.94E-09 | 0.114 | 0.244 | | 0.49 | | 0.829 | |  |
| rs3127582 | 3.18E-09 | 0.121 | 0.255 | | 0.5 | | 0.81 | |  |
| rs3106165 | 3.14E-09 | 0.126 | 0.23 | | 0.536 | | 0.728 | |  |
| rs79390162 | 1.45E-09 | 0.12 | 0.234 | | 0.535 | | 0.824 | |  |
| rs932925 | 5.82E-09 | 0.116 | 0.244 | | 0.506 | | 0.839 | |  |
| rs3103350 | 2.64E-09 | 0.129 | 0.233 | | 0.551 | | 0.785 | |  |
| rs11755965 | 2.37E-08 | 0.132 | 0.231 | | 0.554 | | 0.787 | |  |
| rs3120149 | 2.26E-08 | 0.117 | 0.219 | | 0.532 | | 0.846 | |  |
| rs3120148 | 1.03E-08 | 0.139 | 0.229 | | 0.566 | | 0.774 | |  |
| rs3120147 | 1.07E-08 | 0.15 | 0.231 | | 0.575 | | 0.779 | |  |
| rs3127585 | 1.03E-08 | 0.137 | 0.23 | | 0.571 | | 0.787 | |  |
| rs3106175 | 8.70E-09 | 0.144 | 0.223 | | 0.577 | | 0.782 | |  |
| rs3127583 | 9.13E-09 | 0.14 | 0.215 | | 0.593 | | 0.763 | |  |
| rs3120146 | 1.86E-08 | 0.122 | 0.216 | | 0.534 | | 0.827 | |  |
| rs3106174 | 8.92E-09 | 0.142 | 0.216 | | 0.587 | | 0.77 | |  |
| rs3120145 | 8.62E-09 | 0.141 | 0.218 | | 0.589 | | 0.767 | |  |
| rs3106173 | 8.60E-09 | 0.151 | 0.213 | | 0.585 | | 0.764 | |  |
| rs3106172 | 8.80E-09 | 0.152 | 0.213 | | 0.586 | | 0.763 | |  |
| rs3106171 | 6.32E-09 | 0.137 | 0.198 | | 0.565 | | 0.775 | |  |
| rs76725059 | 8.52E-09 | 0.138 | 0.211 | | 0.597 | | 0.78 | |  |
| rs3106170 | 1.82E-08 | 0.124 | 0.215 | | 0.559 | | 0.83 | |  |
| rs3106169 | 8.48E-09 | 0.143 | 0.213 | | 0.612 | | 0.768 | |  |
| rs3127598 | 8.39E-09 | 0.14 | 0.215 | | 0.601 | | 0.776 | |  |
| rs3127597 | 1.82E-08 | 0.123 | 0.213 | | 0.54 | | 0.824 | |  |
| rs3106168 | 8.39E-09 | 0.149 | 0.195 | | 0.645 | | 0.738 | |  |
| rs3125057 | 8.38E-09 | 0.14 | 0.214 | | 0.597 | | 0.766 | |  |
| rs12192401 | 1.85E-08 | 0.125 | 0.217 | | 0.559 | | 0.822 | |  |
| rs1848830 | 9.11E-09 | 0.139 | 0.215 | | 0.597 | | 0.766 | |  |
| rs3125055 | 8.19E-09 | 0.137 | 0.242 | | 0.605 | | 0.803 | |  |
| rs3125053 | 8.65E-09 | 0.139 | 0.219 | | 0.602 | | 0.757 | |  |
| rs3106167 | 8.60E-09 | 0.133 | 0.224 | | 0.588 | | 0.782 | |  |
| rs3127591 | 8.66E-09 | 0.307 | 0.218 | | 0.614 | | 0.807 | |  |
| rs3125052 | 7.77E-09 | 0.141 | 0.266 | | 0.644 | | 0.765 | |  |
| rs3125051 | 6.64E-09 | 0.144 | 0.207 | | 0.602 | | 0.776 | |  |
| rs3120140 | 2.42E-08 | 0.136 | 0.215 | | 0.593 | | 0.828 | |  |
| rs3103349 | 8.52E-09 | 0.138 | 0.184 | | 0.552 | | 0.797 | |  |
| rs3125050 | 1.45E-08 | 0.125 | 0.198 | | 0.485 | | 0.879 | |  |
| rs3120139 | 7.75E-09 | 0.14 | 0.195 | | 0.569 | | 0.781 | |  |
| rs3103348 | 2.66E-08 | 0.165 | 0.257 | | 0.55 | | 0.726 | |  |
| rs3103347 | 3.09E-08 | 0.159 | 0.275 | | 0.547 | | 0.715 | |  |
| rs6933264 | 3.58E-08 | 0.164 | 0.259 | | 0.525 | | 0.76 | |  |
| rs6911381 | 3.54E-08 | 0.161 | 0.259 | | 0.524 | | 0.743 | |  |
| rs9295127 | 1.33E-15 | 0.849 | 0.945 | | 0.249 | | 0.672 | |  |
| rs9295128 | 2.64E-24 | 0.564 | 0.929 | | 0.226 | | 0.836 | |  |
| rs3125049 | 3.96E-08 | 0.164 | 0.17 | | 0.36 | | 0.794 | |  |
| rs9457906 | 1.81E-12 | 0.877 | 0.982 | | 0.26 | | 0.648 | |  |
| rs1510226 | 6.71E-13 | 0.582 | 0.848 | | 0.266 | | 0.387 | |  |
| rs9457925 | 1.13E-08 | 0.75 | 0.772 | | 0.0684 | | 0.328 | |  |
| rs4709431 | 5.31E-09 | 0.875 | 0.879 | | 0.0589 | | 0.396 | |  |
| rs2292334 | 2.26E-09 | 0.994 | 0.351 | | 0.343 | | 0.31 | |  |
| rs2048327 | 2.46E-09 | 1 | 0.381 | | 0.302 | | 0.283 | |  |
| rs7769879 | 2.97E-09 | 0.972 | 0.344 | | 0.287 | | 0.31 | |  |
| rs9355288 | 4.28E-09 | 0.96 | 0.3 | | 0.26 | | 0.306 | |  |
| rs3918285 | 7.25E-09 | 0.951 | 0.318 | | 0.286 | | 0.273 | |  |
| rs3918286 | 7.36E-09 | 0.946 | 0.312 | | 0.282 | | 0.279 | |  |
| rs1810126 | 3.95E-09 | 0.869 | 0.367 | | 0.328 | | 0.313 | |  |
| rs3088442 | 5.31E-09 | 0.906 | 0.366 | | 0.318 | | 0.288 | |  |
| rs2063347 | 5.85E-09 | 0.986 | 0.316 | | 0.212 | | 0.327 | |  |
| rs3106162 | 5.87E-09 | 0.939 | 0.318 | | 0.195 | | 0.364 | |  |
| rs2063346 | 4.78E-09 | 0.881 | 0.318 | | 0.181 | | 0.368 | |  |
| rs1510224 | 8.22E-09 | 0.979 | 0.912 | | 0.12 | | 0.529 | |  |
| rs9457927 | 1.11E-21 | 0.867 | 0.965 | | 0.106 | | 0.545 | |  |
| rs117791490 | 9.30E-09 | 0.937 | 0.944 | | 0.0994 | | 0.519 | |  |
| rs117733303 | 2.14E-08 | 0.927 | 0.986 | | 0.122 | | 0.594 | |  |
| rs7452960 | 1.02E-09 | 0.892 | 0.968 | | 0.105 | | 0.54 | |  |
| rs12194138 | 1.28E-08 | 0.736 | 0.0998 | | 0.322 | | 0.479 | |  |
| rs3798220 | 4.66E-09 | 0.924 | 0.971 | | 0.103 | | 0.485 | |  |
| rs11751605 | 1.61E-08 | 0.491 | 0.13 | | 0.3 | | 0.64 | |  |
| rs118039278 | 4.38E-37 | 0.0907 | 0.676 | | 0.103 | | 0.931 | |  |
| rs74617384 | 1.72E-38 | 0.0877 | 0.76 | | 0.0832 | | 0.959 | |  |
| rs55730499 | 5.39E-39 | 0.0686 | 0.732 | | 0.11 | | 0.89 | |  |
| rs10455872 | 5.73E-39 | 0.0564 | 0.698 | | 0.0879 | | 0.882 | |  |
| rs140570886 | 2.80E-21 | 0.73 | 0.675 | | 0.103 | | 0.688 | |  |
| rs7770628 | 7.52E-09 | 0.857 | 0.522 | | 0.964 | | 0.876 | |  |
| rs12175867 | 5.60E-09 | 0.575 | 0.409 | | 0.241 | | 0.718 | |  |
| rs6926458 | 5.36E-09 | 0.658 | 0.382 | | 0.225 | | 0.684 | |  |
| rs9355297 | 6.61E-09 | 0.592 | 0.395 | | 0.238 | | 0.696 | |  |
| rs6940254 | 7.11E-09 | 0.596 | 0.388 | | 0.238 | | 0.689 | |  |
| rs35600881 | 8.32E-09 | 0.598 | 0.395 | | 0.233 | | 0.686 | |  |
| rs6926896 | 2.82E-09 | 0.628 | 0.37 | | 0.245 | | 0.683 | |  |
| rs13202636 | 2.43E-09 | 0.637 | 0.369 | | 0.23 | | 0.669 | |  |
| rs6905073 | 1.91E-09 | 0.788 | 0.135 | | 0.512 | | 0.908 | |  |
| rs62442784 | 3.34E-09 | 0.678 | 0.331 | | 0.246 | | 0.6 | |  |
| rs4708876 | 2.11E-09 | 0.867 | 0.505 | | 0.994 | | 0.937 | |  |
| rs7770685 | 3.54E-09 | 0.604 | 0.35 | | 0.239 | | 0.652 | |  |
| rs56393506 | 2.37E-25 | 0.392 | 0.486 | | 0.134 | | 0.567 | |  |
| rs2315065 | 2.88E-34 | 0.0692 | 0.702 | | 0.274 | | 0.859 | |  |
| rs6935921 | 7.35E-10 | 0.139 | 0.193 | | 0.277 | | 0.581 | |  |
| rs6455698 | 5.21E-10 | 0.131 | 0.155 | | 0.382 | | 0.969 | |  |
| rs186696265 | 3.35E-30 | 0.934 | 0.743 | | 0.0813 | | 0.521 | |  |
| rs7772437 | 6.12E-08 | 0.364 | 0.956 | | 0.873 | | 0.988 | |  |
| rs1009124 | 7.07E-08 | 0.368 | 0.957 | | 0.861 | | 0.975 | |  |
| rs783144 | 1.77E-09 | 0.0779 | 0.142 | | 0.483 | | 0.603 | |  |
| rs7452732 | 6.53E-08 | 0.348 | 0.952 | | 0.845 | | 0.988 | |  |
| rs1965091 | 5.21E-08 | 0.365 | 0.955 | | 0.875 | | 0.994 | |  |
| rs2144723 | 9.40E-08 | 0.385 | 0.984 | | 0.823 | | 0.986 | |  |
| rs4252185 | 1.64E-32 | 0.0763 | 0.949 | | 0.289 | | 0.828 | |  |
| rs117340856 | 5.93E-08 | 0.587 | 0.999 | | 0.0264 | | 0.159 | |  |
| rs74985118 | 1.15E-08 | 0.109 | 0.687 | | 0.503 | | 0.745 | |  |
| rs74625856 | 5.87E-09 | 0.166 | 0.666 | | 0.557 | | 0.897 | |  |
| rs143843429 | 1.91E-10 | 0.798 | 0.833 | | 0.152 | | 0.476 | |  |
| rs141766382 | 1.63E-08 | 0.961 | 0.667 | | 0.468 | | 0.776 | |  |
| rs7788833 | 1.24E-09 | 0.423 | 0.76 | | 0.456 | | 0.624 | |  |
| rs7788972 | 1.10E-09 | 0.41 | 0.76 | | 0.481 | | 0.623 | |  |
| rs2074633 | 6.80E-10 | 0.853 | 0.604 | | 0.528 | | 0.591 | |  |
| rs7798197 | 7.32E-10 | 0.431 | 0.86 | | 0.537 | | 0.764 | |  |
| rs2023936 | 3.77E-10 | 0.415 | 0.793 | | 0.466 | | 0.589 | |  |
| rs28688791 | 6.54E-10 | 0.444 | 0.812 | | 0.542 | | 0.571 | |  |
| rs2526620 | 1.23E-08 | 0.127 | 0.46 | | 0.868 | | 0.614 | |  |
| rs2107595 | 8.05E-11 | 0.391 | 0.333 | | 0.908 | | 0.84 | |  |
| rs57301765 | 6.09E-10 | 0.623 | 0.265 | | 0.955 | | 0.999 | |  |
| rs11768743 | 1.22E-08 | 0.572 | 0.145 | | 0.35 | | 0.362 | |  |
| rs11556924 | 5.34E-11 | 0.848 | 0.000219 | | 0.202 | | 0.641 | |  |
| rs56179563 | 7.46E-10 | 0.713 | 0.000409 | | 0.147 | | 0.552 | |  |
| rs3918226 | 1.69E-09 | 0.0155 | 0.925 | | 0.839 | | 0.205 | |  |
| rs7041637 | 6.33E-22 | 0.453 | 0.0362 | | 0.419 | | 0.408 | |  |
| rs67226702 | 9.92E-10 | 0.48 | 0.0781 | | 0.829 | | 0.709 | |  |
| rs3731257 | 2.37E-17 | 0.646 | 0.0415 | | 0.857 | | 0.44 | |  |
| rs3731249 | 1.44E-08 | 0.471 | 0.419 | | 0.561 | | 0.241 | |  |
| rs3731239 | 7.55E-14 | 0.231 | 0.144 | | 0.236 | | 0.755 | |  |
| rs36228834 | 1.67E-08 | 0.45 | 0.376 | | 0.486 | | 0.209 | |  |
| rs113650570 | 1.63E-08 | 0.379 | 0.294 | | 0.511 | | 0.213 | |  |
| rs2518723 | 1.18E-23 | 0.997 | 0.0195 | | 0.537 | | 0.582 | |  |
| rs61271866 | 2.43E-25 | 0.316 | 0.0754 | | 0.508 | | 0.7 | |  |
| rs3218020 | 6.92E-40 | 0.902 | 0.0039 | | 0.39 | | 0.334 | |  |
| rs3218012 | 1.62E-25 | 0.862 | 0.00861 | | 0.693 | | 0.211 | |  |
| rs3218009 | 1.27E-14 | 0.276 | 0.45 | | 0.18 | | 0.341 | |  |
| rs3217992 | 1.03E-42 | 0.906 | 0.00336 | | 0.341 | | 0.408 | |  |
| rs1063192 | 1.64E-10 | 0.916 | 0.044 | | 0.465 | | 0.222 | |  |
| rs2069418 | 9.16E-22 | 0.906 | 0.0454 | | 0.985 | | 0.318 | |  |
| rs573687 | 2.41E-25 | 0.955 | 0.0315 | | 0.911 | | 0.734 | |  |
| rs13298881 | 3.75E-10 | 0.867 | 0.465 | | 0.485 | | 0.152 | |  |
| rs545226 | 1.06E-22 | 0.777 | 0.00124 | | 0.907 | | 0.871 | |  |
| rs10811640 | 2.01E-25 | 0.752 | 0.0126 | | 0.515 | | 0.254 | |  |
| rs10757263 | 3.55E-25 | 0.749 | 0.0134 | | 0.534 | | 0.257 | |  |
| rs10811641 | 2.28E-36 | 0.981 | 0.00229 | | 0.756 | | 0.444 | |  |
| rs1101330 | 3.57E-26 | 0.967 | 0.0387 | | 0.902 | | 0.753 | |  |
| rs2106120 | 1.54E-25 | 0.618 | 0.0132 | | 0.553 | | 0.28 | |  |
| rs2106119 | 1.06E-25 | 0.716 | 0.0126 | | 0.525 | | 0.24 | |  |
| rs643319 | 2.37E-23 | 0.882 | 0.0149 | | 0.516 | | 0.641 | |  |
| rs7044859 | 9.30E-26 | 0.692 | 0.0134 | | 0.51 | | 0.285 | |  |
| rs523096 | 4.18E-27 | 0.733 | 0.0557 | | 0.698 | | 0.303 | |  |
| rs62556547 | 2.79E-14 | 0.228 | 0.498 | | 0.265 | | 0.259 | |  |
| rs518394 | 2.55E-27 | 0.782 | 0.0548 | | 0.686 | | 0.316 | |  |
| rs10757264 | 7.10E-20 | 0.941 | 0.00525 | | 0.262 | | 0.572 | |  |
| rs490005 | 1.61E-19 | 0.861 | 0.00665 | | 0.335 | | 0.517 | |  |
| rs597816 | 8.15E-22 | 0.957 | 0.0243 | | 0.883 | | 0.653 | |  |
| rs568447 | 5.27E-28 | 0.749 | 0.00115 | | 0.839 | | 0.986 | |  |
| rs567453 | 7.52E-25 | 0.959 | 0.00617 | | 0.597 | | 0.682 | |  |
| rs581876 | 1.92E-29 | 0.882 | 0.0294 | | 0.858 | | 0.793 | |  |
| rs10965212 | 7.76E-32 | 0.766 | 0.0131 | | 0.905 | | 0.362 | |  |
| rs504318 | 4.61E-25 | 0.918 | 0.00482 | | 0.615 | | 0.676 | |  |
| rs496892 | 5.69E-25 | 0.932 | 0.00637 | | 0.559 | | 0.593 | |  |
| rs10811643 | 2.37E-26 | 0.759 | 0.00779 | | 0.606 | | 0.251 | |  |
| rs10811644 | 1.89E-26 | 0.741 | 0.00913 | | 0.628 | | 0.246 | |  |
| rs7035484 | 2.13E-26 | 0.765 | 0.00835 | | 0.591 | | 0.236 | |  |
| rs10738604 | 4.84E-40 | 0.951 | 0.00211 | | 0.626 | | 0.564 | |  |
| rs615552 | 4.94E-28 | 0.87 | 0.0354 | | 0.73 | | 0.301 | |  |
| rs613312 | 7.60E-35 | 0.853 | 0.0348 | | 0.509 | | 0.364 | |  |
| rs543830 | 6.18E-35 | 0.867 | 0.0331 | | 0.499 | | 0.37 | |  |
| rs1591136 | 1.04E-32 | 0.738 | 0.00857 | | 0.852 | | 0.335 | |  |
| rs599452 | 6.86E-35 | 0.874 | 0.0337 | | 0.536 | | 0.365 | |  |
| rs62560774 | 2.07E-33 | 0.295 | 0.102 | | 0.261 | | 0.858 | |  |
| rs7049105 | 7.46E-32 | 0.722 | 0.00844 | | 0.826 | | 0.33 | |  |
| rs679038 | 6.21E-35 | 0.88 | 0.0349 | | 0.548 | | 0.372 | |  |
| rs10965215 | 1.26E-32 | 0.706 | 0.0102 | | 0.851 | | 0.443 | |  |
| rs564398 | 9.42E-35 | 0.873 | 0.0499 | | 0.448 | | 0.398 | |  |
| rs4977753 | 3.30E-32 | 0.72 | 0.0108 | | 0.887 | | 0.37 | |  |
| rs7865618 | 3.14E-11 | 0.804 | 0.0431 | | 0.614 | | 0.465 | |  |
| rs10115049 | 1.20E-32 | 0.71 | 0.00862 | | 0.92 | | 0.466 | |  |
| rs634537 | 6.11E-35 | 0.85 | 0.0431 | | 0.548 | | 0.375 | |  |
| rs2157719 | 2.49E-11 | 0.804 | 0.0536 | | 0.673 | | 0.444 | |  |
| rs2151280 | 1.60E-32 | 0.608 | 0.0127 | | 0.818 | | 0.497 | |  |
| rs78572337 | 8.81E-08 | 0.0456 | 0.298 | | 0.256 | | 0.445 | |  |
| rs1008878 | 3.88E-11 | 0.78 | 0.0384 | | 0.67 | | 0.474 | |  |
| rs1556515 | 3.95E-11 | 0.827 | 0.0477 | | 0.64 | | 0.486 | |  |
| rs1333037 | 2.84E-11 | 0.755 | 0.043 | | 0.735 | | 0.51 | |  |
| rs1360590 | 4.00E-28 | 0.627 | 0.0124 | | 0.689 | | 0.41 | |  |
| rs1412830 | 2.60E-30 | 0.906 | 0.0599 | | 0.461 | | 0.574 | |  |
| rs1333036 | 8.28E-33 | 0.614 | 0.0141 | | 0.673 | | 0.433 | |  |
| rs1412829 | 2.19E-35 | 0.689 | 0.0456 | | 0.789 | | 0.46 | |  |
| rs1360589 | 7.71E-11 | 0.547 | 0.0334 | | 0.689 | | 0.44 | |  |
| rs10120806 | 1.63E-26 | 0.413 | 0.0122 | | 0.723 | | 0.474 | |  |
| rs7027950 | 2.28E-28 | 0.423 | 0.0166 | | 0.731 | | 0.487 | |  |
| rs7028268 | 4.98E-48 | 0.616 | 0.00552 | | 0.457 | | 0.687 | |  |
| rs7028570 | 3.59E-28 | 0.442 | 0.0129 | | 0.716 | | 0.523 | |  |
| rs10757265 | 1.07E-28 | 0.453 | 0.0112 | | 0.725 | | 0.501 | |  |
| rs10757266 | 3.17E-28 | 0.416 | 0.00875 | | 0.693 | | 0.492 | |  |
| rs10811645 | 1.77E-26 | 0.393 | 0.00756 | | 0.674 | | 0.401 | |  |
| rs944799 | 7.86E-11 | 0.6 | 0.0117 | | 0.584 | | 0.414 | |  |
| rs944800 | 4.55E-27 | 0.282 | 0.0917 | | 0.333 | | 0.822 | |  |
| rs944801 | 1.49E-10 | 0.682 | 0.0311 | | 0.695 | | 0.433 | |  |
| rs6475604 | 1.41E-10 | 0.6 | 0.032 | | 0.702 | | 0.46 | |  |
| rs10757267 | 5.74E-11 | 0.687 | 0.014 | | 0.589 | | 0.364 | |  |
| rs11790231 | 4.00E-13 | 0.381 | 0.151 | | 0.638 | | 0.08 | |  |
| rs10965219 | 5.87E-11 | 0.673 | 0.0128 | | 0.615 | | 0.357 | |  |
| rs7027048 | 5.72E-11 | 0.686 | 0.0123 | | 0.594 | | 0.354 | |  |
| rs7030641 | 1.38E-10 | 0.652 | 0.0345 | | 0.694 | | 0.458 | |  |
| rs7874604 | 1.55E-28 | 0.727 | 0.0121 | | 0.619 | | 0.368 | |  |
| rs2383204 | 5.78E-11 | 0.698 | 0.0113 | | 0.64 | | 0.322 | |  |
| rs7853090 | 2.31E-10 | 0.469 | 0.0241 | | 0.501 | | 0.633 | |  |
| rs7866783 | 9.98E-11 | 0.523 | 0.0233 | | 0.485 | | 0.648 | |  |
| rs10120688 | 1.83E-10 | 0.364 | 0.00714 | | 0.759 | | 0.788 | |  |
| rs10757268 | 3.48E-10 | 0.387 | 0.143 | | 0.378 | | 0.748 | |  |
| rs2095144 | 3.44E-10 | 0.366 | 0.14 | | 0.386 | | 0.756 | |  |
| rs2383205 | 4.93E-11 | 0.418 | 0.0176 | | 0.393 | | 0.699 | |  |
| rs2184061 | 4.09E-11 | 0.416 | 0.025 | | 0.361 | | 0.797 | |  |
| rs1537378 | 4.96E-11 | 0.401 | 0.0198 | | 0.378 | | 0.716 | |  |
| rs4977754 | 5.92E-08 | 0.928 | 0.497 | | 0.0191 | | 0.524 | |  |
| rs8181050 | 3.71E-11 | 0.42 | 0.0183 | | 0.429 | | 0.723 | |  |
| rs8181047 | 6.39E-10 | 0.431 | 0.144 | | 0.315 | | 0.878 | |  |
| rs10811647 | 4.22E-62 | 0.32 | 0.00858 | | 0.185 | | 0.908 | |  |
| rs1333039 | 3.28E-11 | 0.382 | 0.0194 | | 0.397 | | 0.788 | |  |
| rs10965223 | 1.72E-11 | 0.376 | 0.0175 | | 0.389 | | 0.769 | |  |
| rs10965224 | 2.85E-11 | 0.372 | 0.0188 | | 0.393 | | 0.789 | |  |
| rs10811648 | 3.91E-11 | 0.327 | 0.0226 | | 0.408 | | 0.76 | |  |
| rs10811649 | 4.24E-11 | 0.319 | 0.0217 | | 0.41 | | 0.739 | |  |
| rs10811650 | 2.12E-62 | 0.287 | 0.0089 | | 0.188 | | 0.907 | |  |
| rs10811651 | 3.45E-11 | 0.371 | 0.0183 | | 0.401 | | 0.792 | |  |
| rs4977756 | 4.21E-11 | 0.364 | 0.0202 | | 0.402 | | 0.782 | |  |
| rs4451405 | 1.64E-10 | 0.342 | 0.0289 | | 0.295 | | 0.623 | |  |
| rs10757269 | 9.88E-32 | 0.626 | 0.0436 | | 0.115 | | 0.903 | |  |
| rs9632884 | 4.82E-33 | 0.596 | 0.044 | | 0.115 | | 0.88 | |  |
| rs9632885 | 1.24E-31 | 0.775 | 0.0539 | | 0.0924 | | 0.706 | |  |
| rs10757270 | 2.85E-70 | 0.465 | 0.0136 | | 0.153 | | 0.666 | |  |
| rs1855185 | 2.71E-08 | 0.89 | 0.762 | | 0.339 | | 0.213 | |  |
| rs1831733 | 4.67E-88 | 0.569 | 0.0428 | | 0.0379 | | 0.853 | |  |
| rs10757271 | 2.44E-81 | 0.597 | 0.0594 | | 0.0746 | | 0.816 | |  |
| rs10811652 | 1.68E-34 | 0.571 | 0.055 | | 0.0701 | | 0.787 | |  |
| rs1412832 | 5.14E-11 | 0.305 | 0.108 | | 0.219 | | 0.81 | |  |
| rs10116277 | 1.56E-32 | 0.45 | 0.119 | | 0.0579 | | 0.669 | |  |
| rs10965227 | 2.36E-13 | 0.529 | 0.983 | | 0.304 | | 0.972 | |  |
| rs6475606 | 1.52E-32 | 0.404 | 0.137 | | 0.0468 | | 0.624 | |  |
| rs1547705 | 3.02E-09 | 0.498 | 0.279 | | 0.841 | | 0.425 | |  |
| rs1333040 | 6.94E-13 | 0.555 | 0.168 | | 0.0925 | | 0.636 | |  |
| rs72652409 | 6.09E-08 | 0.439 | 0.152 | | 0.105 | | 0.912 | |  |
| rs1537370 | 4.18E-34 | 0.358 | 0.171 | | 0.0467 | | 0.623 | |  |
| rs1970112 | 1.22E-89 | 0.426 | 0.156 | | 0.0194 | | 0.607 | |  |
| rs66478960 | 5.54E-22 | 0.629 | 0.899 | | 0.239 | | 0.994 | |  |
| rs7857345 | 4.73E-13 | 0.0976 | 0.126 | | 0.155 | | 0.973 | |  |
| rs10738606 | 4.89E-38 | 0.255 | 0.115 | | 0.00712 | | 0.936 | |  |
| rs10738607 | 4.59E-38 | 0.25 | 0.115 | | 0.00746 | | 0.936 | |  |
| rs10757272 | 3.58E-37 | 0.26 | 0.171 | | 0.00676 | | 0.857 | |  |
| rs10757274 | 2.49E-38 | 0.243 | 0.179 | | 0.00468 | | 0.873 | |  |
| rs4977574 | 6.35E-98 | 0.299 | 0.177 | | 0.0046 | | 0.94 | |  |
| rs2891168 | 2.29E-98 | 0.277 | 0.172 | | 0.00453 | | 0.934 | |  |
| rs1537371 | 4.85E-39 | 0.299 | 0.139 | | 0.01 | | 0.933 | |  |
| rs1556516 | 2.87E-39 | 0.285 | 0.14 | | 0.00984 | | 0.897 | |  |
| rs72652478 | 1.06E-08 | 0.213 | 0.856 | | 0.583 | | 0.366 | |  |
| rs7859727 | 3.01E-38 | 0.244 | 0.173 | | 0.00975 | | 0.818 | |  |
| rs62555370 | 2.05E-22 | 0.347 | 0.136 | | 0.12 | | 0.299 | |  |
| rs1537372 | 2.50E-83 | 0.125 | 0.0672 | | 0.0376 | | 0.801 | |  |
| rs1537373 | 1.77E-39 | 0.271 | 0.166 | | 0.0116 | | 0.903 | |  |
| rs1333042 | 2.60E-39 | 0.282 | 0.143 | | 0.0115 | | 0.872 | |  |
| rs7859362 | 2.63E-39 | 0.182 | 0.145 | | 0.0228 | | 0.936 | |  |
| rs10757275 | 2.54E-96 | 0.161 | 0.166 | | 0.0122 | | 0.962 | |  |
| rs6475609 | 6.96E-18 | 0.191 | 0.115 | | 0.024 | | 0.922 | |  |
| rs1333043 | 2.86E-39 | 0.185 | 0.11 | | 0.0226 | | 0.921 | |  |
| rs62555371 | 2.45E-22 | 0.338 | 0.117 | | 0.129 | | 0.294 | |  |
| rs1412834 | 1.05E-17 | 0.202 | 0.14 | | 0.0249 | | 0.914 | |  |
| rs7341786 | 2.70E-17 | 0.218 | 0.122 | | 0.0285 | | 0.921 | |  |
| rs7341791 | 2.53E-17 | 0.212 | 0.124 | | 0.0286 | | 0.919 | |  |
| rs10511701 | 2.90E-35 | 0.203 | 0.102 | | 0.0174 | | 0.803 | |  |
| rs10733376 | 1.34E-17 | 0.2 | 0.139 | | 0.0218 | | 0.963 | |  |
| rs2383206 | 1.53E-17 | 0.18 | 0.155 | | 0.018 | | 0.893 | |  |
| rs944797 | 2.27E-17 | 0.106 | 0.136 | | 0.0152 | | 0.48 | |  |
| rs1004638 | 9.56E-18 | 0.207 | 0.114 | | 0.0203 | | 0.944 | |  |
| rs2383207 | 8.02E-18 | 0.204 | 0.102 | | 0.018 | | 0.953 | |  |
| rs1537374 | 1.01E-17 | 0.221 | 0.115 | | 0.0208 | | 0.912 | |  |
| rs1537375 | 1.12E-36 | 0.213 | 0.129 | | 0.0125 | | 0.995 | |  |
| rs1537376 | 1.39E-17 | 0.188 | 0.135 | | 0.0183 | | 0.898 | |  |
| rs1333045 | 7.33E-15 | 0.285 | 0.232 | | 0.00173 | | 0.555 | |  |
| rs10217586 | 4.44E-16 | 0.2 | 0.163 | | 0.00179 | | 0.636 | |  |
| rs10738610 | 1.78E-96 | 0.222 | 0.103 | | 0.00832 | | 0.853 | |  |
| rs1333046 | 3.47E-96 | 0.241 | 0.101 | | 0.00999 | | 0.898 | |  |
| rs7857118 | 7.64E-38 | 0.267 | 0.0742 | | 0.0198 | | 0.949 | |  |
| rs10757277 | 5.69E-94 | 0.41 | 0.156 | | 0.00961 | | 0.795 | |  |
| rs10811656 | 9.67E-33 | 0.431 | 0.168 | | 0.00869 | | 0.76 | |  |
| rs10757278 | 1.09E-93 | 0.438 | 0.154 | | 0.0114 | | 0.783 | |  |
| rs1333047 | 1.97E-36 | 0.454 | 0.138 | | 0.0203 | | 0.87 | |  |
| rs10757279 | 4.73E-94 | 0.38 | 0.16 | | 0.00886 | | 0.814 | |  |
| rs4977575 | 1.24E-36 | 0.422 | 0.141 | | 0.0159 | | 0.88 | |  |
| rs1333048 | 5.97E-94 | 0.26 | 0.169 | | 0.0118 | | 0.932 | |  |
| rs1333049 | 3.86E-93 | 0.443 | 0.178 | | 0.0106 | | 0.831 | |  |
| rs1333050 | 1.88E-41 | 0.204 | 0.326 | | 0.231 | | 0.933 | |  |
| rs2519093 | 1.19E-11 | 0.335 | 0.478 | | 0.388 | | 0.847 | |  |
| rs9411378 | 1.95E-08 | 0.364 | 0.557 | | 0.0919 | | 0.303 | |  |
| rs550057 | 4.19E-09 | 0.536 | 0.428 | | 0.164 | | 0.473 | |  |
| rs507666 | 1.64E-11 | 0.327 | 0.544 | | 0.35 | | 0.875 | |  |
| rs532436 | 1.61E-11 | 0.309 | 0.461 | | 0.346 | | 0.863 | |  |
| rs600038 | 7.15E-11 | 0.556 | 0.754 | | 0.6 | | 0.391 | |  |
| rs651007 | 1.19E-10 | 0.55 | 0.796 | | 0.579 | | 0.451 | |  |
| rs579459 | 1.14E-10 | 0.548 | 0.809 | | 0.534 | | 0.427 | |  |
| rs649129 | 1.21E-10 | 0.399 | 0.677 | | 0.261 | | 0.887 | |  |
| rs495828 | 1.29E-10 | 0.686 | 0.822 | | 0.545 | | 0.403 | |  |
| rs635634 | 4.47E-11 | 0.432 | 0.531 | | 0.447 | | 0.442 | |  |
| rs1774240 | 3.35E-08 | 0.618 | 3.94E-05 | | 0.238 | | 0.204 | |  |
| rs12779954 | 2.67E-08 | 0.468 | 0.000805 | | 0.169 | | 0.439 | |  |
| rs11007851 | 2.55E-08 | 0.444 | 0.00109 | | 0.176 | | 0.438 | |  |
| rs1774241 | 1.78E-08 | 0.653 | 3.70E-05 | | 0.222 | | 0.186 | |  |
| rs1832864 | 5.18E-08 | 0.861 | 0.00021 | | 0.211 | | 0.424 | |  |
| rs4749520 | 3.71E-08 | 0.575 | 0.000905 | | 0.367 | | 0.357 | |  |
| rs1418276 | 4.77E-08 | 0.539 | 0.000812 | | 0.316 | | 0.361 | |  |
| rs10826749 | 4.60E-08 | 0.512 | 0.00113 | | 0.312 | | 0.343 | |  |
| rs12259037 | 3.37E-08 | 0.98 | 0.000526 | | 0.271 | | 0.51 | |  |
| rs2487926 | 1.57E-09 | 0.987 | 4.78E-05 | | 0.232 | | 0.408 | |  |
| rs2505132 | 4.32E-08 | 0.752 | 4.16E-05 | | 0.164 | | 0.385 | |  |
| rs2478839 | 5.87E-10 | 0.886 | 6.29E-05 | | 0.131 | | 0.75 | |  |
| rs7071535 | 5.03E-08 | 0.841 | 0.00254 | | 0.47 | | 0.839 | |  |
| rs2014144 | 6.14E-09 | 0.648 | 0.00532 | | 0.484 | | 0.87 | |  |
| rs2487927 | 4.29E-10 | 0.827 | 6.94E-05 | | 0.135 | | 0.746 | |  |
| rs11007864 | 4.08E-08 | 0.471 | 0.00428 | | 0.396 | | 0.981 | |  |
| rs55885169 | 5.58E-08 | 0.885 | 0.00316 | | 0.494 | | 0.843 | |  |
| rs12784436 | 6.99E-08 | 0.542 | 0.00532 | | 0.46 | | 0.999 | |  |
| rs3739998 | 2.44E-09 | 0.663 | 5.10E-05 | | 0.0887 | | 0.924 | |  |
| rs9337951 | 2.30E-08 | 0.472 | 0.00534 | | 0.387 | | 0.536 | |  |
| rs7920682 | 1.06E-08 | 0.547 | 0.00636 | | 0.281 | | 0.729 | |  |
| rs7920686 | 1.17E-08 | 0.55 | 0.00554 | | 0.282 | | 0.73 | |  |
| rs2478835 | 1.04E-09 | 0.699 | 7.49E-05 | | 0.0578 | | 0.858 | |  |
| rs10826751 | 8.10E-09 | 0.573 | 0.00504 | | 0.277 | | 0.742 | |  |
| rs1887318 | 5.05E-11 | 0.903 | 8.35E-05 | | 0.0794 | | 0.993 | |  |
| rs2066333 | 9.81E-10 | 0.778 | 0.00553 | | 0.315 | | 0.907 | |  |
| rs2066334 | 8.93E-09 | 0.842 | 0.00317 | | 0.283 | | 0.882 | |  |
| rs11007869 | 1.78E-08 | 0.807 | 0.00332 | | 0.293 | | 0.89 | |  |
| rs2487928 | 4.41E-11 | 0.875 | 8.42E-05 | | 0.0941 | | 0.972 | |  |
| rs10826753 | 5.62E-09 | 0.393 | 0.00426 | | 0.259 | | 0.93 | |  |
| rs943440 | 1.32E-08 | 0.688 | 0.00511 | | 0.281 | | 0.726 | |  |
| rs943439 | 5.25E-08 | 0.78 | 0.00389 | | 0.223 | | 0.941 | |  |
| rs943438 | 6.40E-10 | 0.958 | 0.0046 | | 0.406 | | 0.933 | |  |
| rs1342150 | 9.69E-10 | 0.893 | 0.000788 | | 0.167 | | 0.858 | |  |
| rs4749523 | 2.61E-08 | 0.852 | 0.00922 | | 0.319 | | 0.81 | |  |
| rs4749524 | 3.02E-08 | 0.886 | 0.00581 | | 0.274 | | 0.785 | |  |
| rs61841115 | 1.75E-08 | 0.814 | 0.00922 | | 0.322 | | 0.828 | |  |
| rs7071112 | 1.97E-08 | 0.851 | 0.00556 | | 0.249 | | 0.797 | |  |
| rs2505083 | 1.57E-10 | 0.811 | 0.000228 | | 0.184 | | 0.868 | |  |
| rs7071848 | 1.91E-08 | 0.816 | 0.0058 | | 0.28 | | 0.823 | |  |
| rs7089816 | 1.95E-08 | 0.798 | 0.00566 | | 0.28 | | 0.827 | |  |
| rs2505084 | 4.38E-10 | 0.87 | 0.000717 | | 0.208 | | 0.986 | |  |
| rs4948582 | 1.50E-12 | 0.138 | 0.834 | | 0.829 | | 0.0391 | |  |
| rs4948583 | 1.77E-12 | 0.141 | 0.822 | | 0.847 | | 0.0387 | |  |
| rs10899953 | 2.94E-12 | 0.17 | 0.785 | | 0.815 | | 0.0425 | |  |
| rs4948792 | 4.80E-12 | 0.168 | 0.762 | | 0.833 | | 0.0302 | |  |
| rs11238782 | 1.23E-08 | 0.0166 | 0.9 | | 0.941 | | 0.0841 | |  |
| rs11528455 | 9.31E-10 | 0.0172 | 0.895 | | 0.983 | | 0.0916 | |  |
| rs898551 | 8.21E-10 | 0.0151 | 0.914 | | 0.964 | | 0.104 | |  |
| rs7088951 | 6.84E-10 | 0.0143 | 0.905 | | 0.973 | | 0.125 | |  |
| rs10899954 | 3.55E-10 | 0.0146 | 0.93 | | 0.988 | | 0.109 | |  |
| rs7924201 | 8.22E-10 | 0.0138 | 0.86 | | 0.963 | | 0.109 | |  |
| rs1977341 | 3.62E-08 | 0.46 | 0.655 | | 0.464 | | 0.0788 | |  |
| rs898549 | 1.45E-09 | 0.0128 | 0.947 | | 0.94 | | 0.119 | |  |
| rs66887775 | 6.47E-10 | 0.0153 | 0.939 | | 0.835 | | 0.137 | |  |
| rs10899955 | 2.26E-10 | 0.0132 | 0.943 | | 0.905 | | 0.118 | |  |
| rs10899956 | 6.63E-10 | 0.0128 | 0.917 | | 0.88 | | 0.127 | |  |
| rs1460542 | 1.87E-08 | 0.514 | 0.701 | | 0.608 | | 0.113 | |  |
| rs4948800 | 3.71E-10 | 0.00976 | 0.987 | | 0.99 | | 0.126 | |  |
| rs12359058 | 2.68E-10 | 0.0129 | 0.962 | | 0.967 | | 0.124 | |  |
| rs2085797 | 4.47E-10 | 0.0107 | 0.949 | | 0.966 | | 0.132 | |  |
| rs10899957 | 6.59E-10 | 0.0155 | 0.881 | | 0.836 | | 0.113 | |  |
| rs2279555 | 1.67E-08 | 0.477 | 0.562 | | 0.538 | | 0.058 | |  |
| rs11238794 | 2.16E-13 | 0.0841 | 0.42 | | 0.968 | | 0.0524 | |  |
| rs10793510 | 1.24E-09 | 0.489 | 0.69 | | 0.352 | | 0.0565 | |  |
| rs4948803 | 5.60E-11 | 0.372 | 0.747 | | 0.715 | | 0.159 | |  |
| rs10899960 | 5.29E-11 | 0.378 | 0.748 | | 0.594 | | 0.143 | |  |
| rs1870635 | 8.33E-15 | 0.128 | 0.36 | | 0.692 | | 0.0424 | |  |
| rs1870634 | 5.55E-15 | 0.105 | 0.405 | | 0.749 | | 0.0454 | |  |
| rs6593377 | 1.24E-10 | 0.342 | 0.722 | | 0.748 | | 0.14 | |  |
| rs988738 | 6.10E-11 | 0.416 | 0.801 | | 0.694 | | 0.139 | |  |
| rs988739 | 8.88E-15 | 0.134 | 0.357 | | 0.706 | | 0.0413 | |  |
| rs2862856 | 5.93E-08 | 0.878 | 0.556 | | 0.172 | | 0.0263 | |  |
| rs4145117 | 6.63E-12 | 0.0159 | 0.703 | | 0.554 | | 0.252 | |  |
| rs4145118 | 5.30E-11 | 0.437 | 0.806 | | 0.707 | | 0.138 | |  |
| rs7918198 | 1.22E-10 | 0.424 | 0.801 | | 0.705 | | 0.14 | |  |
| rs4322346 | 1.91E-08 | 0.394 | 0.779 | | 0.6 | | 0.147 | |  |
| rs9633746 | 1.60E-09 | 0.39 | 0.779 | | 0.591 | | 0.145 | |  |
| rs4307670 | 1.33E-08 | 0.543 | 0.706 | | 0.327 | | 0.0573 | |  |
| rs4948805 | 6.84E-11 | 0.393 | 0.781 | | 0.601 | | 0.147 | |  |
| rs9633745 | 9.68E-12 | 0.0162 | 0.739 | | 0.566 | | 0.25 | |  |
| rs11818958 | 8.93E-12 | 0.0161 | 0.744 | | 0.557 | | 0.236 | |  |
| rs11238800 | 6.85E-12 | 0.016 | 0.73 | | 0.567 | | 0.25 | |  |
| rs1032408 | 9.48E-11 | 0.387 | 0.761 | | 0.6 | | 0.147 | |  |
| rs10793511 | 1.10E-11 | 0.017 | 0.739 | | 0.558 | | 0.253 | |  |
| rs10736824 | 1.58E-10 | 0.383 | 0.769 | | 0.606 | | 0.147 | |  |
| rs4948806 | 8.75E-09 | 0.538 | 0.711 | | 0.291 | | 0.0572 | |  |
| rs4948589 | 1.43E-10 | 0.423 | 0.791 | | 0.731 | | 0.124 | |  |
| rs4948590 | 1.43E-11 | 0.0155 | 0.722 | | 0.558 | | 0.253 | |  |
| rs7090343 | 1.22E-11 | 0.0163 | 0.716 | | 0.559 | | 0.252 | |  |
| rs4948591 | 1.88E-11 | 0.018 | 0.745 | | 0.598 | | 0.243 | |  |
| rs4948592 | 6.36E-13 | 0.154 | 0.356 | | 0.702 | | 0.0438 | |  |
| rs4948593 | 1.71E-10 | 0.401 | 0.802 | | 0.74 | | 0.138 | |  |
| rs4948594 | 1.72E-10 | 0.4 | 0.813 | | 0.762 | | 0.137 | |  |
| rs4948808 | 8.10E-11 | 0.442 | 0.887 | | 0.804 | | 0.116 | |  |
| rs4948809 | 2.81E-10 | 0.412 | 0.803 | | 0.784 | | 0.138 | |  |
| rs10793512 | 3.12E-10 | 0.398 | 0.807 | | 0.773 | | 0.137 | |  |
| rs10793513 | 3.93E-14 | 0.143 | 0.316 | | 0.721 | | 0.052 | |  |
| rs7091447 | 1.55E-11 | 0.017 | 0.708 | | 0.571 | | 0.285 | |  |
| rs4997087 | 2.19E-10 | 0.374 | 0.864 | | 0.764 | | 0.124 | |  |
| rs7908336 | 1.48E-10 | 0.366 | 0.867 | | 0.775 | | 0.121 | |  |
| rs10793514 | 5.75E-14 | 0.139 | 0.32 | | 0.706 | | 0.0511 | |  |
| rs10793515 | 8.30E-12 | 0.0151 | 0.697 | | 0.575 | | 0.29 | |  |
| rs10899962 | 1.62E-10 | 0.373 | 0.919 | | 0.776 | | 0.124 | |  |
| rs6593384 | 8.44E-10 | 0.311 | 0.915 | | 0.722 | | 0.135 | |  |
| rs6593385 | 5.57E-11 | 0.31 | 0.909 | | 0.709 | | 0.138 | |  |
| rs4948811 | 3.63E-11 | 0.304 | 0.919 | | 0.722 | | 0.151 | |  |
| rs1873756 | 1.72E-11 | 0.0167 | 0.609 | | 0.517 | | 0.376 | |  |
| rs7478408 | 1.09E-11 | 0.0127 | 0.636 | | 0.541 | | 0.317 | |  |
| rs10899963 | 4.68E-12 | 0.0144 | 0.663 | | 0.659 | | 0.251 | |  |
| rs7922924 | 6.46E-11 | 0.688 | 0.905 | | 0.783 | | 0.104 | |  |
| rs7923335 | 3.90E-11 | 0.802 | 0.94 | | 0.572 | | 0.109 | |  |
| rs10899965 | 7.39E-12 | 0.0343 | 0.672 | | 0.614 | | 0.236 | |  |
| rs4948595 | 2.28E-11 | 0.687 | 0.896 | | 0.707 | | 0.0944 | |  |
| rs4948812 | 1.70E-11 | 0.655 | 0.869 | | 0.693 | | 0.0949 | |  |
| rs12573239 | 2.28E-11 | 0.663 | 0.879 | | 0.685 | | 0.0956 | |  |
| rs11238808 | 9.09E-12 | 0.0329 | 0.698 | | 0.578 | | 0.244 | |  |
| rs7084564 | 2.25E-11 | 0.663 | 0.875 | | 0.685 | | 0.0965 | |  |
| rs10793516 | 1.92E-11 | 0.0391 | 0.683 | | 0.545 | | 0.239 | |  |
| rs7074248 | 5.01E-11 | 0.643 | 0.863 | | 0.704 | | 0.103 | |  |
| rs10899967 | 5.84E-11 | 0.617 | 0.915 | | 0.64 | | 0.107 | |  |
| rs10899968 | 6.95E-11 | 0.616 | 0.881 | | 0.653 | | 0.106 | |  |
| rs10793517 | 2.56E-11 | 0.0315 | 0.663 | | 0.554 | | 0.246 | |  |
| rs10793518 | 5.07E-09 | 0.773 | 0.683 | | 0.241 | | 0.045 | |  |
| rs4948816 | 2.15E-11 | 0.345 | 0.465 | | 0.351 | | 0.0327 | |  |
| rs4948596 | 2.15E-11 | 0.348 | 0.468 | | 0.35 | | 0.0336 | |  |
| rs4948818 | 2.27E-11 | 0.0398 | 0.575 | | 0.557 | | 0.28 | |  |
| rs4948819 | 3.58E-11 | 0.337 | 0.46 | | 0.345 | | 0.0325 | |  |
| rs1472168 | 2.58E-11 | 0.0616 | 0.557 | | 0.596 | | 0.228 | |  |
| rs6593388 | 1.53E-09 | 0.664 | 0.352 | | 0.0743 | | 0.00889 | |  |
| rs10736825 | 2.46E-11 | 0.522 | 0.523 | | 0.311 | | 0.0304 | |  |
| rs4948820 | 2.46E-11 | 0.522 | 0.521 | | 0.314 | | 0.0301 | |  |
| rs1352999 | 2.70E-11 | 0.0913 | 0.549 | | 0.992 | | 0.149 | |  |
| rs2902339 | 2.44E-11 | 0.526 | 0.521 | | 0.311 | | 0.0301 | |  |
| rs2185946 | 2.42E-11 | 0.526 | 0.521 | | 0.312 | | 0.0305 | |  |
| rs2185947 | 2.40E-11 | 0.521 | 0.524 | | 0.313 | | 0.0305 | |  |
| rs1602716 | 2.42E-11 | 0.527 | 0.524 | | 0.31 | | 0.0304 | |  |
| rs10899970 | 3.22E-11 | 0.0913 | 0.583 | | 0.956 | | 0.136 | |  |
| rs7919657 | 2.26E-11 | 0.561 | 0.516 | | 0.295 | | 0.0305 | |  |
| rs9332446 | 1.52E-11 | 0.0654 | 0.565 | | 0.609 | | 0.241 | |  |
| rs10899971 | 2.78E-11 | 0.0912 | 0.584 | | 0.971 | | 0.149 | |  |
| rs7903881 | 2.34E-11 | 0.529 | 0.515 | | 0.291 | | 0.0326 | |  |
| rs1388975 | 2.93E-11 | 0.534 | 0.52 | | 0.289 | | 0.0314 | |  |
| rs955582 | 2.94E-11 | 0.533 | 0.52 | | 0.292 | | 0.0306 | |  |
| rs955583 | 2.36E-11 | 0.545 | 0.526 | | 0.294 | | 0.0311 | |  |
| rs955584 | 3.22E-11 | 0.0903 | 0.597 | | 0.978 | | 0.136 | |  |
| rs6593393 | 1.98E-09 | 0.69 | 0.338 | | 0.0663 | | 0.00901 | |  |
| rs10899973 | 1.59E-11 | 0.0687 | 0.584 | | 0.613 | | 0.246 | |  |
| rs1492711 | 5.76E-11 | 0.545 | 0.485 | | 0.288 | | 0.0296 | |  |
| rs11238817 | 2.60E-11 | 0.0614 | 0.588 | | 0.603 | | 0.239 | |  |
| rs12570314 | 3.25E-11 | 0.0924 | 0.657 | | 0.973 | | 0.14 | |  |
| rs1833024 | 3.20E-08 | 0.953 | 0.761 | | 0.127 | | 0.0182 | |  |
| rs11238818 | 3.31E-11 | 0.0601 | 0.571 | | 0.592 | | 0.237 | |  |
| rs7475017 | 2.33E-09 | 0.527 | 0.478 | | 0.231 | | 0.0287 | |  |
| rs11238822 | 3.65E-11 | 0.0835 | 0.667 | | 0.954 | | 0.141 | |  |
| rs1891883 | 5.25E-11 | 0.476 | 0.409 | | 0.21 | | 0.025 | |  |
| rs7917534 | 3.62E-11 | 0.484 | 0.424 | | 0.223 | | 0.0287 | |  |
| rs4615977 | 3.14E-11 | 0.486 | 0.418 | | 0.23 | | 0.0282 | |  |
| rs2051120 | 2.36E-09 | 0.623 | 0.319 | | 0.0402 | | 0.00774 | |  |
| rs1565660 | 3.79E-11 | 0.487 | 0.438 | | 0.217 | | 0.0284 | |  |
| rs7903121 | 2.22E-11 | 0.0475 | 0.583 | | 0.579 | | 0.224 | |  |
| rs1873757 | 3.00E-11 | 0.0726 | 0.669 | | 0.963 | | 0.136 | |  |
| rs2047009 | 2.75E-11 | 0.0437 | 0.594 | | 0.587 | | 0.239 | |  |
| rs10508881 | 5.36E-10 | 0.0481 | 0.59 | | 0.709 | | 0.198 | |  |
| rs9804352 | 3.91E-10 | 0.0493 | 0.61 | | 0.715 | | 0.211 | |  |
| rs2639469 | 8.12E-09 | 0.438 | 0.278 | | 0.603 | | 0.129 | |  |
| rs2639468 | 1.69E-08 | 0.507 | 0.269 | | 0.624 | | 0.126 | |  |
| rs2818916 | 5.21E-09 | 0.272 | 0.246 | | 0.844 | | 0.0895 | |  |
| rs2624688 | 9.85E-08 | 0.86 | 0.131 | | 0.0488 | | 0.00897 | |  |
| rs2624695 | 1.39E-10 | 0.131 | 0.801 | | 0.468 | | 0.353 | |  |
| rs268328 | 2.12E-10 | 0.783 | 0.512 | | 0.0603 | | 0.0278 | |  |
| rs2128363 | 2.06E-10 | 0.132 | 0.79 | | 0.461 | | 0.386 | |  |
| rs2624694 | 1.26E-10 | 0.136 | 0.817 | | 0.524 | | 0.405 | |  |
| rs2804029 | 1.34E-10 | 0.108 | 0.858 | | 0.392 | | 0.363 | |  |
| rs2818904 | 8.55E-11 | 0.108 | 0.891 | | 0.417 | | 0.366 | |  |
| rs2639463 | 1.23E-10 | 0.148 | 0.984 | | 0.37 | | 0.388 | |  |
| rs2804027 | 1.17E-10 | 0.157 | 0.994 | | 0.39 | | 0.362 | |  |
| rs2639465 | 9.16E-11 | 0.159 | 0.999 | | 0.376 | | 0.365 | |  |
| rs2818912 | 3.06E-09 | 0.806 | 0.957 | | 0.829 | | 0.261 | |  |
| rs2624691 | 6.21E-11 | 0.134 | 0.965 | | 0.281 | | 0.326 | |  |
| rs2624692 | 1.21E-10 | 0.129 | 0.959 | | 0.269 | | 0.321 | |  |
| rs1482478 | 1.00E-10 | 0.0932 | 0.989 | | 0.396 | | 0.381 | |  |
| rs2802490 | 4.00E-10 | 0.191 | 0.606 | | 0.257 | | 0.415 | |  |
| rs2818914 | 2.25E-09 | 0.198 | 0.604 | | 0.268 | | 0.422 | |  |
| rs2804021 | 3.21E-10 | 0.197 | 0.584 | | 0.28 | | 0.425 | |  |
| rs142539486 | 8.29E-08 | 0.98 | 0.795 | | 0.0432 | | 0.148 | |  |
| rs2804042 | 4.53E-08 | 0.289 | 0.885 | | 0.22 | | 0.575 | |  |
| rs11238889 | 6.21E-10 | 0.816 | 0.3 | | 0.61 | | 0.592 | |  |
| rs11238890 | 5.61E-10 | 0.856 | 0.279 | | 0.62 | | 0.552 | |  |
| rs11238891 | 2.40E-09 | 0.816 | 0.277 | | 0.584 | | 0.592 | |  |
| rs11238892 | 2.24E-09 | 0.822 | 0.286 | | 0.588 | | 0.593 | |  |
| rs11238894 | 2.11E-09 | 0.813 | 0.296 | | 0.6 | | 0.588 | |  |
| rs1704228 | 6.06E-09 | 0.65 | 0.317 | | 0.615 | | 0.644 | |  |
| rs1680636 | 2.69E-09 | 0.674 | 0.306 | | 0.573 | | 0.629 | |  |
| rs11594667 | 3.91E-09 | 0.683 | 0.294 | | 0.585 | | 0.618 | |  |
| rs1704223 | 3.20E-09 | 0.681 | 0.311 | | 0.604 | | 0.594 | |  |
| rs2664860 | 5.54E-08 | 0.656 | 0.505 | | 0.859 | | 0.777 | |  |
| rs1615053 | 4.98E-09 | 0.697 | 0.292 | | 0.553 | | 0.627 | |  |
| rs1704226 | 3.32E-09 | 0.694 | 0.3 | | 0.554 | | 0.63 | |  |
| rs11238898 | 3.74E-09 | 0.697 | 0.299 | | 0.546 | | 0.621 | |  |
| rs61855739 | 3.21E-09 | 0.697 | 0.297 | | 0.538 | | 0.614 | |  |
| rs1545845 | 9.82E-08 | 0.675 | 0.526 | | 0.833 | | 0.759 | |  |
| rs61855740 | 3.18E-09 | 0.723 | 0.295 | | 0.527 | | 0.599 | |  |
| rs11595159 | 3.17E-09 | 0.708 | 0.304 | | 0.531 | | 0.621 | |  |
| rs11238901 | 3.18E-09 | 0.728 | 0.285 | | 0.548 | | 0.601 | |  |
| rs11238902 | 3.42E-09 | 0.71 | 0.292 | | 0.541 | | 0.608 | |  |
| rs35339178 | 3.43E-08 | 0.83 | 0.591 | | 0.419 | | 0.27 | |  |
| rs61855743 | 4.70E-09 | 0.723 | 0.289 | | 0.53 | | 0.614 | |  |
| rs1509931 | 2.81E-08 | 0.693 | 0.547 | | 0.79 | | 0.832 | |  |
| rs1704214 | 2.92E-08 | 0.693 | 0.537 | | 0.795 | | 0.831 | |  |
| rs1509929 | 2.26E-08 | 0.827 | 0.616 | | 0.432 | | 0.252 | |  |
| rs4948857 | 2.80E-08 | 0.69 | 0.536 | | 0.778 | | 0.835 | |  |
| rs10793525 | 2.39E-08 | 0.842 | 0.627 | | 0.422 | | 0.24 | |  |
| rs10899997 | 2.08E-08 | 0.828 | 0.637 | | 0.421 | | 0.239 | |  |
| rs11238904 | 2.57E-08 | 0.689 | 0.527 | | 0.788 | | 0.834 | |  |
| rs61855758 | 2.57E-08 | 0.687 | 0.526 | | 0.781 | | 0.84 | |  |
| rs11817838 | 2.57E-08 | 0.706 | 0.532 | | 0.781 | | 0.815 | |  |
| rs11238905 | 2.56E-08 | 0.707 | 0.529 | | 0.781 | | 0.818 | |  |
| rs11595946 | 2.18E-09 | 0.732 | 0.278 | | 0.522 | | 0.619 | |  |
| rs11598296 | 2.29E-08 | 0.714 | 0.536 | | 0.797 | | 0.832 | |  |
| rs11598314 | 2.52E-08 | 0.703 | 0.536 | | 0.796 | | 0.843 | |  |
| rs11238906 | 8.31E-08 | 0.711 | 0.537 | | 0.784 | | 0.828 | |  |
| rs12415866 | 2.42E-08 | 0.716 | 0.535 | | 0.791 | | 0.824 | |  |
| rs11238907 | 2.49E-08 | 0.701 | 0.544 | | 0.816 | | 0.858 | |  |
| rs11238908 | 4.11E-08 | 0.837 | 0.262 | | 0.614 | | 0.57 | |  |
| rs11238909 | 3.70E-08 | 0.846 | 0.258 | | 0.605 | | 0.547 | |  |
| rs10899998 | 4.08E-08 | 0.844 | 0.258 | | 0.617 | | 0.572 | |  |
| rs7917089 | 2.11E-08 | 0.712 | 0.526 | | 0.807 | | 0.835 | |  |
| rs61857460 | 2.58E-09 | 0.724 | 0.286 | | 0.493 | | 0.617 | |  |
| rs7909598 | 1.73E-08 | 0.805 | 0.613 | | 0.433 | | 0.251 | |  |
| rs1704229 | 5.24E-08 | 0.838 | 0.495 | | 0.781 | | 0.774 | |  |
| rs10899999 | 1.72E-08 | 0.691 | 0.526 | | 0.765 | | 0.9 | |  |
| rs11591406 | 3.82E-09 | 0.731 | 0.296 | | 0.523 | | 0.628 | |  |
| rs1623851 | 1.68E-08 | 0.824 | 0.624 | | 0.447 | | 0.273 | |  |
| rs1704231 | 1.19E-09 | 0.476 | 0.499 | | 0.904 | | 0.802 | |  |
| rs1626459 | 1.90E-08 | 0.694 | 0.526 | | 0.823 | | 0.893 | |  |
| rs1627329 | 1.63E-08 | 0.829 | 0.622 | | 0.457 | | 0.277 | |  |
| rs10160170 | 9.27E-09 | 0.47 | 0.586 | | 0.761 | | 0.98 | |  |
| rs10900000 | 7.94E-11 | 0.75 | 0.238 | | 0.615 | | 0.623 | |  |
| rs7902040 | 2.40E-10 | 0.82 | 0.3 | | 0.672 | | 0.68 | |  |
| rs10900001 | 2.52E-10 | 0.809 | 0.309 | | 0.674 | | 0.658 | |  |
| rs11238911 | 2.57E-10 | 0.799 | 0.309 | | 0.666 | | 0.659 | |  |
| rs11238912 | 2.41E-10 | 0.805 | 0.289 | | 0.669 | | 0.689 | |  |
| rs11238915 | 2.34E-10 | 0.797 | 0.275 | | 0.64 | | 0.684 | |  |
| rs11238916 | 2.35E-10 | 0.797 | 0.273 | | 0.64 | | 0.684 | |  |
| rs10900002 | 2.31E-10 | 0.811 | 0.26 | | 0.62 | | 0.672 | |  |
| rs10900003 | 2.05E-10 | 0.8 | 0.265 | | 0.637 | | 0.687 | |  |
| rs7100370 | 2.23E-10 | 0.801 | 0.266 | | 0.636 | | 0.685 | |  |
| rs7082177 | 2.21E-10 | 0.8 | 0.268 | | 0.638 | | 0.69 | |  |
| rs7086639 | 2.18E-10 | 0.801 | 0.269 | | 0.648 | | 0.698 | |  |
| rs7900072 | 2.27E-10 | 0.795 | 0.28 | | 0.646 | | 0.704 | |  |
| rs11238923 | 2.05E-10 | 0.783 | 0.281 | | 0.653 | | 0.743 | |  |
| rs7097076 | 1.96E-10 | 0.79 | 0.278 | | 0.658 | | 0.734 | |  |
| rs1680639 | 2.22E-10 | 0.783 | 0.285 | | 0.655 | | 0.743 | |  |
| rs2804031 | 2.55E-10 | 0.758 | 0.29 | | 0.664 | | 0.737 | |  |
| rs2664856 | 2.57E-10 | 0.762 | 0.299 | | 0.656 | | 0.726 | |  |
| rs11238925 | 2.55E-10 | 0.768 | 0.301 | | 0.658 | | 0.748 | |  |
| rs11238927 | 2.53E-10 | 0.784 | 0.302 | | 0.662 | | 0.764 | |  |
| rs12355852 | 2.50E-10 | 0.78 | 0.302 | | 0.654 | | 0.745 | |  |
| rs58540467 | 3.87E-10 | 0.907 | 0.318 | | 0.689 | | 0.706 | |  |
| rs906944 | 3.08E-10 | 0.899 | 0.317 | | 0.697 | | 0.727 | |  |
| rs58129751 | 2.23E-10 | 0.866 | 0.337 | | 0.742 | | 0.718 | |  |
| rs1482473 | 6.90E-09 | 0.515 | 0.869 | | 0.308 | | 0.18 | |  |
| rs17155641 | 4.90E-09 | 0.574 | 0.866 | | 0.285 | | 0.16 | |  |
| rs11238929 | 1.93E-10 | 0.918 | 0.311 | | 0.714 | | 0.718 | |  |
| rs1680641 | 7.18E-09 | 0.535 | 0.867 | | 0.324 | | 0.174 | |  |
| rs1704222 | 1.92E-10 | 0.89 | 0.316 | | 0.698 | | 0.734 | |  |
| rs1704221 | 2.99E-10 | 0.885 | 0.319 | | 0.687 | | 0.694 | |  |
| rs966161 | 1.89E-10 | 0.872 | 0.322 | | 0.686 | | 0.729 | |  |
| rs1905150 | 8.08E-09 | 0.545 | 0.851 | | 0.314 | | 0.174 | |  |
| rs11238933 | 2.18E-10 | 0.895 | 0.328 | | 0.71 | | 0.749 | |  |
| rs11238935 | 3.12E-10 | 0.894 | 0.33 | | 0.707 | | 0.749 | |  |
| rs1746034 | 2.20E-10 | 0.884 | 0.33 | | 0.703 | | 0.738 | |  |
| rs11511598 | 2.27E-10 | 0.893 | 0.331 | | 0.702 | | 0.752 | |  |
| rs61857482 | 3.15E-10 | 0.895 | 0.33 | | 0.703 | | 0.747 | |  |
| rs61857484 | 2.77E-10 | 0.879 | 0.332 | | 0.693 | | 0.758 | |  |
| rs61857485 | 2.29E-10 | 0.887 | 0.327 | | 0.703 | | 0.759 | |  |
| rs61857486 | 2.63E-10 | 0.889 | 0.326 | | 0.714 | | 0.733 | |  |
| rs11238937 | 2.43E-10 | 0.88 | 0.327 | | 0.702 | | 0.745 | |  |
| rs11238940 | 2.57E-10 | 0.894 | 0.331 | | 0.707 | | 0.755 | |  |
| rs11238943 | 2.68E-10 | 0.897 | 0.331 | | 0.706 | | 0.759 | |  |
| rs11238944 | 2.70E-10 | 0.897 | 0.331 | | 0.706 | | 0.759 | |  |
| rs11517251 | 2.73E-10 | 0.891 | 0.333 | | 0.7 | | 0.749 | |  |
| rs11492882 | 2.78E-10 | 0.891 | 0.333 | | 0.7 | | 0.75 | |  |
| rs11238946 | 2.67E-10 | 0.898 | 0.333 | | 0.705 | | 0.76 | |  |
| rs11238947 | 1.18E-10 | 0.878 | 0.328 | | 0.708 | | 0.724 | |  |
| rs11517223 | 1.10E-10 | 0.81 | 0.35 | | 0.733 | | 0.717 | |  |
| rs11511571 | 2.99E-10 | 0.885 | 0.334 | | 0.725 | | 0.758 | |  |
| rs1704217 | 1.52E-10 | 0.868 | 0.343 | | 0.715 | | 0.749 | |  |
| rs1704218 | 1.27E-10 | 0.809 | 0.376 | | 0.737 | | 0.71 | |  |
| rs7079375 | 1.43E-10 | 0.851 | 0.356 | | 0.706 | | 0.756 | |  |
| rs596152 | 1.31E-10 | 0.868 | 0.339 | | 0.679 | | 0.767 | |  |
| rs1746042 | 1.27E-10 | 0.874 | 0.347 | | 0.691 | | 0.764 | |  |
| rs556434 | 6.88E-11 | 0.869 | 0.334 | | 0.695 | | 0.734 | |  |
| rs677465 | 1.39E-10 | 0.856 | 0.323 | | 0.66 | | 0.77 | |  |
| rs11592445 | 1.28E-10 | 0.878 | 0.339 | | 0.673 | | 0.743 | |  |
| rs1619661 | 1.14E-10 | 0.889 | 0.346 | | 0.618 | | 0.821 | |  |
| rs674746 | 1.64E-10 | 0.875 | 0.361 | | 0.643 | | 0.774 | |  |
| rs673778 | 1.12E-10 | 0.877 | 0.364 | | 0.644 | | 0.775 | |  |
| rs673354 | 1.27E-10 | 0.877 | 0.363 | | 0.643 | | 0.774 | |  |
| rs1746044 | 6.61E-11 | 0.962 | 0.336 | | 0.642 | | 0.776 | |  |
| rs545025 | 9.29E-12 | 0.991 | 0.458 | | 0.819 | | 0.737 | |  |
| rs629213 | 8.21E-12 | 0.986 | 0.464 | | 0.819 | | 0.737 | |  |
| rs617019 | 9.27E-12 | 0.986 | 0.464 | | 0.816 | | 0.736 | |  |
| rs583489 | 1.80E-11 | 0.79 | 0.474 | | 0.919 | | 0.6 | |  |
| rs680091 | 3.75E-12 | 0.935 | 0.462 | | 0.784 | | 0.724 | |  |
| rs676966 | 4.96E-12 | 0.929 | 0.46 | | 0.799 | | 0.72 | |  |
| rs661697 | 6.98E-12 | 0.973 | 0.477 | | 0.818 | | 0.734 | |  |
| rs498810 | 3.01E-11 | 0.641 | 0.65 | | 0.989 | | 0.671 | |  |
| rs649192 | 8.32E-12 | 0.863 | 0.506 | | 0.934 | | 0.566 | |  |
| rs494207 | 8.71E-12 | 0.672 | 0.551 | | 0.842 | | 0.598 | |  |
| rs646890 | 8.60E-12 | 0.82 | 0.516 | | 0.913 | | 0.577 | |  |
| rs2760670 | 8.51E-12 | 0.876 | 0.508 | | 0.925 | | 0.551 | |  |
| rs2476352 | 3.93E-08 | 0.877 | 0.0299 | | 0.643 | | 0.823 | |  |
| rs617542 | 8.19E-12 | 0.872 | 0.494 | | 0.939 | | 0.543 | |  |
| rs617035 | 1.20E-11 | 0.881 | 0.492 | | 0.902 | | 0.571 | |  |
| rs605414 | 1.02E-11 | 0.859 | 0.498 | | 0.938 | | 0.56 | |  |
| rs2781543 | 1.04E-11 | 0.869 | 0.493 | | 0.936 | | 0.558 | |  |
| rs486234 | 1.36E-08 | 0.423 | 0.0272 | | 0.289 | | 0.941 | |  |
| rs589655 | 8.92E-13 | 0.818 | 0.509 | | 0.887 | | 0.588 | |  |
| rs587375 | 1.03E-11 | 0.765 | 0.639 | | 0.922 | | 0.583 | |  |
| rs684666 | 1.70E-11 | 0.761 | 0.656 | | 0.924 | | 0.6 | |  |
| rs684196 | 1.57E-11 | 0.761 | 0.659 | | 0.92 | | 0.6 | |  |
| rs683297 | 1.15E-11 | 0.776 | 0.648 | | 0.925 | | 0.59 | |  |
| rs541483 | 2.44E-10 | 0.779 | 0.684 | | 0.908 | | 0.667 | |  |
| rs535176 | 1.36E-11 | 0.775 | 0.647 | | 0.919 | | 0.575 | |  |
| rs635612 | 1.68E-11 | 0.657 | 0.614 | | 0.928 | | 0.55 | |  |
| rs474281 | 3.08E-11 | 0.792 | 0.67 | | 0.92 | | 0.588 | |  |
| rs473501 | 1.77E-11 | 0.754 | 0.657 | | 0.938 | | 0.571 | |  |
| rs622472 | 1.38E-11 | 0.757 | 0.668 | | 0.911 | | 0.545 | |  |
| rs513391 | 1.96E-11 | 0.751 | 0.657 | | 0.914 | | 0.586 | |  |
| rs510785 | 2.34E-11 | 0.761 | 0.658 | | 0.928 | | 0.564 | |  |
| rs607609 | 1.52E-11 | 0.778 | 0.654 | | 0.922 | | 0.569 | |  |
| rs607592 | 1.38E-11 | 0.78 | 0.648 | | 0.933 | | 0.569 | |  |
| rs2576355 | 1.44E-11 | 0.758 | 0.651 | | 0.968 | | 0.563 | |  |
| rs2576354 | 4.47E-10 | 0.757 | 0.602 | | 0.919 | | 0.607 | |  |
| rs606700 | 8.27E-11 | 0.793 | 0.599 | | 0.878 | | 0.564 | |  |
| rs485838 | 4.23E-10 | 0.811 | 0.611 | | 0.888 | | 0.534 | |  |
| rs606314 | 1.23E-12 | 0.848 | 0.503 | | 0.868 | | 0.625 | |  |
| rs605425 | 4.33E-11 | 0.771 | 0.659 | | 0.926 | | 0.569 | |  |
| rs687175 | 8.80E-12 | 0.81 | 0.736 | | 0.991 | | 0.61 | |  |
| rs559580 | 6.15E-11 | 0.814 | 0.755 | | 0.997 | | 0.68 | |  |
| rs559469 | 2.21E-11 | 0.805 | 0.741 | | 0.988 | | 0.614 | |  |
| rs2437935 | 6.98E-09 | 0.903 | 0.0496 | | 0.408 | | 0.776 | |  |
| rs535949 | 1.54E-11 | 0.809 | 0.736 | | 0.998 | | 0.609 | |  |
| rs684521 | 2.85E-11 | 0.823 | 0.76 | | 0.972 | | 0.619 | |  |
| rs534079 | 1.64E-11 | 0.789 | 0.748 | | 0.994 | | 0.618 | |  |
| rs671765 | 1.99E-11 | 0.801 | 0.754 | | 0.869 | | 0.619 | |  |
| rs670056 | 1.94E-11 | 0.796 | 0.734 | | 0.999 | | 0.623 | |  |
| rs504799 | 1.64E-11 | 0.797 | 0.736 | | 0.998 | | 0.618 | |  |
| rs503859 | 1.66E-11 | 0.794 | 0.746 | | 0.992 | | 0.584 | |  |
| rs501120 | 1.39E-11 | 0.787 | 0.729 | | 0.982 | | 0.578 | |  |
| rs471451 | 1.86E-12 | 0.825 | 0.935 | | 0.549 | | 0.617 | |  |
| rs579058 | 1.77E-11 | 0.78 | 0.738 | | 0.97 | | 0.579 | |  |
| rs622956 | 1.80E-11 | 0.788 | 0.749 | | 0.965 | | 0.569 | |  |
| rs915083 | 1.32E-11 | 0.799 | 0.742 | | 0.978 | | 0.574 | |  |
| rs554568 | 1.72E-11 | 0.81 | 0.72 | | 0.963 | | 0.556 | |  |
| rs554565 | 1.73E-11 | 0.811 | 0.721 | | 0.959 | | 0.56 | |  |
| rs607760 | 2.09E-11 | 0.783 | 0.744 | | 0.967 | | 0.57 | |  |
| rs523297 | 1.73E-12 | 0.822 | 0.63 | | 0.737 | | 0.626 | |  |
| rs605956 | 2.89E-09 | 0.434 | 0.048 | | 0.153 | | 0.963 | |  |
| rs522293 | 2.07E-11 | 0.751 | 0.753 | | 0.963 | | 0.565 | |  |
| rs605445 | 2.11E-11 | 0.746 | 0.789 | | 0.925 | | 0.575 | |  |
| rs604674 | 2.01E-11 | 0.756 | 0.754 | | 0.954 | | 0.572 | |  |
| rs518594 | 1.77E-11 | 0.767 | 0.742 | | 0.963 | | 0.553 | |  |
| rs492152 | 1.98E-11 | 0.75 | 0.754 | | 0.96 | | 0.55 | |  |
| rs665855 | 2.16E-11 | 0.729 | 0.747 | | 0.953 | | 0.566 | |  |
| rs487465 | 2.27E-11 | 0.759 | 0.774 | | 0.96 | | 0.552 | |  |
| rs573141 | 2.16E-11 | 0.742 | 0.756 | | 0.955 | | 0.552 | |  |
| rs634963 | 2.54E-11 | 0.771 | 0.716 | | 0.972 | | 0.484 | |  |
| rs479596 | 7.80E-13 | 0.877 | 0.673 | | 0.943 | | 0.452 | |  |
| rs622725 | 6.14E-12 | 0.898 | 0.56 | | 0.754 | | 0.537 | |  |
| rs475926 | 4.07E-09 | 0.433 | 0.0444 | | 0.152 | | 0.937 | |  |
| rs620828 | 2.29E-11 | 0.761 | 0.705 | | 0.967 | | 0.474 | |  |
| rs620356 | 2.88E-11 | 0.762 | 0.711 | | 0.947 | | 0.483 | |  |
| rs607363 | 2.63E-11 | 0.753 | 0.706 | | 0.949 | | 0.478 | |  |
| rs694425 | 7.29E-11 | 0.723 | 0.697 | | 0.928 | | 0.545 | |  |
| rs528668 | 2.57E-11 | 0.736 | 0.695 | | 0.977 | | 0.484 | |  |
| rs527785 | 2.58E-11 | 0.755 | 0.705 | | 0.949 | | 0.484 | |  |
| rs620205 | 2.61E-11 | 0.75 | 0.706 | | 0.948 | | 0.477 | |  |
| rs493874 | 2.46E-11 | 0.781 | 0.686 | | 0.933 | | 0.468 | |  |
| rs642222 | 3.38E-11 | 0.731 | 0.699 | | 0.943 | | 0.47 | |  |
| rs552794 | 4.04E-11 | 0.73 | 0.69 | | 0.925 | | 0.477 | |  |
| rs2437936 | 1.28E-12 | 0.819 | 0.683 | | 0.969 | | 0.492 | |  |
| rs3123689 | 4.39E-11 | 0.727 | 0.7 | | 0.956 | | 0.479 | |  |
| rs494192 | 1.93E-11 | 0.725 | 0.695 | | 0.948 | | 0.476 | |  |
| rs494045 | 2.18E-12 | 0.822 | 0.679 | | 0.957 | | 0.486 | |  |
| rs1746046 | 1.96E-08 | 0.428 | 0.0429 | | 0.157 | | 0.983 | |  |
| rs1632484 | 1.41E-12 | 0.822 | 0.695 | | 0.959 | | 0.472 | |  |
| rs1746047 | 4.41E-11 | 0.722 | 0.704 | | 0.959 | | 0.48 | |  |
| rs1746048 | 6.49E-11 | 0.695 | 0.748 | | 0.909 | | 0.41 | |  |
| rs1746049 | 7.09E-13 | 0.819 | 0.72 | | 0.883 | | 0.395 | |  |
| rs1746050 | 6.28E-13 | 0.82 | 0.725 | | 0.889 | | 0.388 | |  |
| rs1657346 | 2.98E-12 | 0.932 | 0.599 | | 0.706 | | 0.459 | |  |
| rs1746052 | 2.95E-10 | 0.811 | 0.751 | | 0.877 | | 0.557 | |  |
| rs1657345 | 4.23E-12 | 0.918 | 0.813 | | 0.886 | | 0.536 | |  |
| rs1657344 | 4.12E-11 | 0.814 | 0.728 | | 0.981 | | 0.468 | |  |
| rs2505734 | 6.90E-08 | 0.718 | 0.384 | | 0.215 | | 0.9 | |  |
| rs2437934 | 5.37E-08 | 0.655 | 0.323 | | 0.281 | | 0.859 | |  |
| rs1412445 | 6.90E-12 | 0.22 | 0.804 | | 0.189 | | 0.308 | |  |
| rs1320496 | 2.92E-11 | 0.833 | 0.204 | | 0.402 | | 0.596 | |  |
| rs1412444 | 5.15E-12 | 0.197 | 0.815 | | 0.196 | | 0.289 | |  |
| rs1332329 | 8.46E-12 | 0.217 | 0.992 | | 0.137 | | 0.285 | |  |
| rs2246949 | 2.47E-10 | 0.862 | 0.204 | | 0.205 | | 0.486 | |  |
| rs2246942 | 7.59E-12 | 0.185 | 0.928 | | 0.115 | | 0.308 | |  |
| rs2246941 | 8.25E-12 | 0.176 | 0.963 | | 0.125 | | 0.306 | |  |
| rs2246833 | 1.33E-11 | 0.195 | 0.772 | | 0.145 | | 0.28 | |  |
| rs2246828 | 2.73E-11 | 0.263 | 0.74 | | 0.355 | | 0.144 | |  |
| rs1051338 | 9.64E-12 | 0.284 | 0.855 | | 0.265 | | 0.0836 | |  |
| rs2250781 | 2.92E-10 | 0.778 | 0.253 | | 0.213 | | 0.457 | |  |
| rs2250645 | 4.46E-11 | 0.272 | 0.717 | | 0.285 | | 0.138 | |  |
| rs2250644 | 2.84E-11 | 0.183 | 0.768 | | 0.141 | | 0.28 | |  |
| rs2250398 | 4.27E-10 | 0.771 | 0.273 | | 0.226 | | 0.434 | |  |
| rs2243548 | 4.45E-10 | 0.791 | 0.27 | | 0.22 | | 0.413 | |  |
| rs2243547 | 2.14E-11 | 0.329 | 0.817 | | 0.202 | | 0.0711 | |  |
| rs1332328 | 5.24E-12 | 0.201 | 0.951 | | 0.114 | | 0.229 | |  |
| rs1332327 | 1.06E-11 | 0.329 | 0.816 | | 0.248 | | 0.117 | |  |
| rs1332326 | 1.17E-09 | 0.845 | 0.26 | | 0.201 | | 0.395 | |  |
| rs2266008 | 3.10E-09 | 0.934 | 0.954 | | 0.354 | | 0.0949 | |  |
| rs17115100 | 7.29E-09 | 0.954 | 0.351 | | 0.363 | | 0.0975 | |  |
| rs1004467 | 5.66E-09 | 0.864 | 0.446 | | 0.331 | | 0.101 | |  |
| rs3824755 | 1.58E-08 | 0.897 | 0.416 | | 0.359 | | 0.0881 | |  |
| rs138009835 | 1.52E-08 | 0.967 | 0.361 | | 0.383 | | 0.0931 | |  |
| rs11191416 | 4.65E-09 | 0.992 | 0.396 | | 0.378 | | 0.0913 | |  |
| rs150786824 | 1.20E-08 | 0.959 | 0.427 | | 0.383 | | 0.0817 | |  |
| rs112468006 | 1.40E-08 | 0.988 | 0.413 | | 0.355 | | 0.0777 | |  |
| rs3824754 | 2.97E-08 | 0.992 | 0.423 | | 0.362 | | 0.0809 | |  |
| rs4409766 | 1.12E-08 | 0.897 | 0.492 | | 0.371 | | 0.0795 | |  |
| rs11191425 | 1.80E-08 | 0.987 | 0.399 | | 0.4 | | 0.0972 | |  |
| rs11191426 | 2.19E-08 | 0.971 | 0.441 | | 0.368 | | 0.0916 | |  |
| rs7098825 | 6.54E-09 | 0.885 | 0.429 | | 0.284 | | 0.101 | |  |
| rs17878846 | 1.42E-08 | 0.954 | 0.415 | | 0.279 | | 0.0829 | |  |
| rs77335224 | 1.44E-08 | 0.897 | 0.52 | | 0.251 | | 0.105 | |  |
| rs3740390 | 4.46E-08 | 0.489 | 0.178 | | 0.716 | | 0.32 | |  |
| rs113282265 | 9.07E-08 | 0.483 | 0.151 | | 0.747 | | 0.296 | |  |
| rs11191454 | 5.90E-08 | 0.418 | 0.237 | | 0.783 | | 0.181 | |  |
| rs7475853 | 7.50E-08 | 0.395 | 0.232 | | 0.802 | | 0.177 | |  |
| rs17115213 | 7.56E-08 | 0.395 | 0.221 | | 0.79 | | 0.166 | |  |
| rs12411886 | 9.58E-08 | 0.518 | 0.193 | | 0.612 | | 0.136 | |  |
| rs10509759 | 8.63E-08 | 0.484 | 0.204 | | 0.644 | | 0.128 | |  |
| rs12414777 | 6.47E-08 | 0.465 | 0.0225 | | 0.573 | | 0.755 | |  |
| rs11191472 | 9.11E-08 | 0.525 | 0.239 | | 0.599 | | 0.138 | |  |
| rs10883806 | 6.80E-08 | 0.514 | 0.234 | | 0.619 | | 0.138 | |  |
| rs11191474 | 8.31E-08 | 0.516 | 0.233 | | 0.625 | | 0.146 | |  |
| rs11191475 | 6.67E-08 | 0.517 | 0.234 | | 0.619 | | 0.137 | |  |
| rs78260931 | 8.16E-08 | 0.507 | 0.233 | | 0.613 | | 0.142 | |  |
| rs11191479 | 9.83E-08 | 0.461 | 0.213 | | 0.566 | | 0.171 | |  |
| rs11191484 | 6.20E-08 | 0.449 | 0.258 | | 0.542 | | 0.156 | |  |
| rs10883815 | 8.79E-08 | 0.438 | 0.232 | | 0.578 | | 0.169 | |  |
| rs112390216 | 9.31E-08 | 0.425 | 0.228 | | 0.574 | | 0.174 | |  |
| rs11191499 | 7.10E-08 | 0.411 | 0.24 | | 0.637 | | 0.173 | |  |
| rs11191502 | 6.81E-08 | 0.42 | 0.236 | | 0.621 | | 0.182 | |  |
| rs113554404 | 6.50E-08 | 0.42 | 0.237 | | 0.614 | | 0.189 | |  |
| rs11191505 | 5.54E-08 | 0.431 | 0.242 | | 0.583 | | 0.168 | |  |
| rs74444347 | 7.05E-08 | 0.426 | 0.223 | | 0.621 | | 0.186 | |  |
| rs12221335 | 5.60E-08 | 0.431 | 0.213 | | 0.61 | | 0.187 | |  |
| rs11191514 | 7.96E-08 | 0.412 | 0.231 | | 0.572 | | 0.175 | |  |
| rs77787671 | 8.45E-08 | 0.422 | 0.182 | | 0.598 | | 0.171 | |  |
| rs11191515 | 6.39E-08 | 0.474 | 0.198 | | 0.565 | | 0.146 | |  |
| rs113970872 | 8.81E-08 | 0.441 | 0.184 | | 0.593 | | 0.168 | |  |
| rs11191517 | 9.07E-08 | 0.441 | 0.188 | | 0.598 | | 0.167 | |  |
| rs11191521 | 8.56E-08 | 0.392 | 0.24 | | 0.595 | | 0.109 | |  |
| rs11191522 | 9.80E-08 | 0.384 | 0.223 | | 0.591 | | 0.108 | |  |
| rs11191531 | 9.81E-08 | 0.44 | 0.223 | | 0.604 | | 0.116 | |  |
| rs11191548 | 5.66E-08 | 0.346 | 0.142 | | 0.566 | | 0.0777 | |  |
| rs79237883 | 9.70E-08 | 0.347 | 0.24 | | 0.623 | | 0.106 | |  |
| rs34747231 | 9.38E-08 | 0.353 | 0.237 | | 0.63 | | 0.1 | |  |
| rs79780963 | 7.54E-08 | 0.485 | 0.208 | | 0.766 | | 0.396 | |  |
| rs12360647 | 3.22E-08 | 0.9 | 0.312 | | 0.583 | | 0.0944 | |  |
| rs10840293 | 1.28E-08 | 0.727 | 0.407 | | 0.505 | | 0.226 | |  |
| rs10840294 | 3.06E-08 | 0.799 | 0.31 | | 0.504 | | 0.149 | |  |
| rs10743117 | 3.22E-08 | 0.832 | 0.312 | | 0.534 | | 0.146 | |  |
| rs10743118 | 2.12E-08 | 0.825 | 0.298 | | 0.551 | | 0.145 | |  |
| rs10840295 | 5.32E-08 | 0.806 | 0.274 | | 0.536 | | 0.175 | |  |
| rs10840296 | 2.59E-08 | 0.832 | 0.31 | | 0.579 | | 0.139 | |  |
| rs9943599 | 2.11E-08 | 0.766 | 0.277 | | 0.551 | | 0.123 | |  |
| rs360158 | 7.01E-08 | 0.884 | 0.375 | | 0.648 | | 0.103 | |  |
| rs360157 | 1.47E-08 | 0.851 | 0.31 | | 0.543 | | 0.143 | |  |
| rs360156 | 7.82E-08 | 0.848 | 0.274 | | 0.538 | | 0.169 | |  |
| rs93139 | 6.65E-08 | 0.916 | 0.308 | | 0.446 | | 0.147 | |  |
| rs93138 | 5.54E-08 | 0.92 | 0.302 | | 0.474 | | 0.147 | |  |
| rs173396 | 3.52E-08 | 0.862 | 0.272 | | 0.486 | | 0.134 | |  |
| rs360153 | 5.16E-08 | 0.824 | 0.237 | | 0.498 | | 0.149 | |  |
| rs491205 | 4.32E-08 | 0.926 | 0.322 | | 0.538 | | 0.119 | |  |
| rs472109 | 7.27E-08 | 0.855 | 0.268 | | 0.539 | | 0.145 | |  |
| rs360136 | 8.05E-08 | 0.883 | 0.276 | | 0.519 | | 0.158 | |  |
| rs974819 | 2.44E-10 | 0.334 | 0.0858 | | 0.441 | | 0.564 | |  |
| rs2019090 | 1.16E-10 | 0.327 | 0.0724 | | 0.527 | | 0.601 | |  |
| rs2128739 | 7.05E-11 | 0.254 | 0.12 | | 0.486 | | 0.366 | |  |
| rs2839812 | 7.10E-11 | 0.246 | 0.115 | | 0.493 | | 0.359 | |  |
| rs11226029 | 1.14E-09 | 0.184 | 0.185 | | 0.649 | | 0.317 | |  |
| rs1384705 | 8.56E-10 | 0.2 | 0.198 | | 0.639 | | 0.313 | |  |
| rs11105328 | 7.78E-08 | 0.835 | 0.658 | | 0.431 | | 0.0693 | |  |
| rs2681472 | 6.17E-11 | 0.413 | 0.694 | | 0.663 | | 0.112 | |  |
| rs2681492 | 8.53E-11 | 0.413 | 0.663 | | 0.639 | | 0.121 | |  |
| rs57481061 | 1.84E-08 | 0.474 | 0.678 | | 0.634 | | 0.0757 | |  |
| rs11105352 | 3.55E-10 | 0.458 | 0.698 | | 0.641 | | 0.125 | |  |
| rs11105353 | 3.10E-10 | 0.457 | 0.696 | | 0.643 | | 0.125 | |  |
| rs11105354 | 1.27E-10 | 0.461 | 0.723 | | 0.632 | | 0.123 | |  |
| rs12579302 | 2.71E-10 | 0.53 | 0.688 | | 0.68 | | 0.129 | |  |
| rs73437338 | 2.50E-08 | 0.578 | 0.629 | | 0.631 | | 0.0713 | |  |
| rs111478946 | 1.80E-10 | 0.528 | 0.655 | | 0.663 | | 0.129 | |  |
| rs17249754 | 2.06E-10 | 0.538 | 0.666 | | 0.657 | | 0.131 | |  |
| rs6538195 | 2.59E-10 | 0.508 | 0.674 | | 0.645 | | 0.147 | |  |
| rs11105364 | 2.58E-10 | 0.487 | 0.669 | | 0.667 | | 0.142 | |  |
| rs73437358 | 2.50E-10 | 0.481 | 0.657 | | 0.642 | | 0.15 | |  |
| rs11105368 | 2.33E-10 | 0.484 | 0.657 | | 0.649 | | 0.141 | |  |
| rs4842675 | 1.94E-10 | 0.488 | 0.657 | | 0.653 | | 0.137 | |  |
| rs11105375 | 3.16E-10 | 0.532 | 0.582 | | 0.581 | | 0.139 | |  |
| rs11105376 | 3.24E-10 | 0.564 | 0.603 | | 0.605 | | 0.11 | |  |
| rs11105378 | 2.78E-10 | 0.542 | 0.585 | | 0.594 | | 0.136 | |  |
| rs12230074 | 3.07E-10 | 0.544 | 0.569 | | 0.59 | | 0.131 | |  |
| rs4842676 | 8.80E-08 | 0.659 | 0.492 | | 0.921 | | 0.154 | |  |
| rs10774624 | 3.23E-09 | 0.343 | 1.94E-05 | | 0.0972 | | 0.692 | |  |
| rs7310615 | 1.67E-09 | 0.143 | 1.53E-05 | | 0.151 | | 0.796 | |  |
| rs3184504 | 1.03E-09 | 0.148 | 1.62E-05 | | 0.165 | | 0.991 | |  |
| rs4766578 | 2.83E-10 | 0.15 | 1.18E-06 | | 0.137 | | 0.885 | |  |
| rs10774625 | 2.69E-10 | 0.149 | 1.24E-06 | | 0.143 | | 0.832 | |  |
| rs7137828 | 1.00E-09 | 0.146 | 1.24E-05 | | 0.208 | | 0.975 | |  |
| rs597808 | 8.75E-09 | 0.145 | 1.12E-05 | | 0.173 | | 0.985 | |  |
| rs653178 | 5.15E-10 | 0.178 | 7.86E-06 | | 0.211 | | 0.951 | |  |
| rs11065979 | 1.93E-10 | 0.0294 | 0.000523 | | 0.0965 | | 0.636 | |  |
| rs11065987 | 2.50E-08 | 0.0455 | 0.000756 | | 0.0946 | | 0.971 | |  |
| rs11065991 | 1.49E-08 | 0.0545 | 0.000967 | | 0.0993 | | 0.985 | |  |
| rs11513729 | 6.97E-09 | 0.0569 | 0.00116 | | 0.14 | | 0.928 | |  |
| rs17696736 | 7.06E-09 | 0.0351 | 0.000306 | | 0.0898 | | 0.752 | |  |
| rs17630235 | 1.01E-08 | 0.037 | 0.00126 | | 0.135 | | 0.897 | |  |
| rs11066188 | 6.72E-09 | 0.0372 | 0.00113 | | 0.168 | | 0.882 | |  |
| rs7953257 | 6.62E-08 | 0.0433 | 0.00116 | | 0.154 | | 0.972 | |  |
| rs11617955 | 3.55E-08 | 0.17 | 0.515 | | 0.306 | | 0.874 | |  |
| rs12866570 | 7.86E-08 | 0.8 | 0.546 | | 0.0379 | | 0.78 | |  |
| rs4773141 | 2.14E-09 | 0.362 | 0.451 | | 0.366 | | 0.777 | |  |
| rs11838776 | 1.83E-10 | 0.428 | 0.68 | | 0.0579 | | 0.445 | |  |
| rs9515201 | 5.46E-10 | 0.275 | 0.854 | | 0.0426 | | 0.511 | |  |
| rs55940034 | 3.49E-10 | 0.265 | 0.915 | | 0.0333 | | 0.511 | |  |
| rs9515203 | 9.33E-10 | 0.198 | 0.102 | | 0.709 | | 0.896 | |  |
| rs10139550 | 1.38E-08 | 0.918 | 0.583 | | 0.421 | | 0.106 | |  |
| rs72743461 | 5.43E-09 | 0.328 | 0.163 | | 0.239 | | 0.596 | |  |
| rs17293632 | 5.72E-09 | 0.338 | 0.167 | | 0.227 | | 0.629 | |  |
| rs56375023 | 1.26E-08 | 0.43 | 0.137 | | 0.293 | | 0.609 | |  |
| rs17228058 | 8.56E-09 | 0.429 | 0.136 | | 0.281 | | 0.583 | |  |
| rs56062135 | 4.52E-09 | 0.463 | 0.132 | | 0.326 | | 0.612 | |  |
| rs59671175 | 8.41E-08 | 5.01E-22 | 0.391 | | 0.000358 | | 0.271 | |  |
| rs1814880 | 1.14E-08 | 2.28E-25 | 0.924 | | 0.000484 | | 0.107 | |  |
| rs1809412 | 1.95E-08 | 2.03E-25 | 0.932 | | 0.000489 | | 0.129 | |  |
| rs11072799 | 1.57E-08 | 2.55E-25 | 0.943 | | 0.00071 | | 0.134 | |  |
| rs1809414 | 1.24E-08 | 2.61E-25 | 0.944 | | 0.000705 | | 0.14 | |  |
| rs1809415 | 1.94E-08 | 5.45E-22 | 0.398 | | 0.00038 | | 0.276 | |  |
| rs1383634 | 2.56E-08 | 7.13E-25 | 0.903 | | 0.000445 | | 0.154 | |  |
| rs1825086 | 2.49E-08 | 5.65E-25 | 0.911 | | 0.000472 | | 0.153 | |  |
| rs1383635 | 2.64E-08 | 5.84E-25 | 0.893 | | 0.000476 | | 0.156 | |  |
| rs2219939 | 3.13E-08 | 4.93E-25 | 0.901 | | 0.00043 | | 0.151 | |  |
| rs12901228 | 2.42E-08 | 4.39E-25 | 0.907 | | 0.000488 | | 0.134 | |  |
| rs58717592 | 7.69E-08 | 4.70E-22 | 0.375 | | 0.000394 | | 0.274 | |  |
| rs11856441 | 3.50E-08 | 3.74E-25 | 0.937 | | 0.000744 | | 0.135 | |  |
| rs56354501 | 7.26E-08 | 1.69E-75 | 0.37 | | 0.0158 | | 0.72 | |  |
| rs8031513 | 7.29E-08 | 5.91E-75 | 0.378 | | 0.0173 | | 0.713 | |  |
| rs56195905 | 6.29E-08 | 1.49E-74 | 0.387 | | 0.018 | | 0.773 | |  |
| rs55834964 | 1.32E-08 | 6.60E-75 | 0.339 | | 0.015 | | 0.89 | |  |
| rs12286 | 4.50E-09 | 3.41E-74 | 0.322 | | 0.00912 | | 0.938 | |  |
| rs7182642 | 3.73E-08 | 6.98E-77 | 0.455 | | 0.016 | | 0.965 | |  |
| rs12906653 | 7.97E-09 | 2.25E-57 | 0.269 | | 0.0406 | | 0.492 | |  |
| rs4887096 | 2.07E-09 | 4.46E-75 | 0.364 | | 0.016 | | 0.713 | |  |
| rs1809419 | 9.06E-09 | 1.04E-74 | 0.39 | | 0.0112 | | 0.655 | |  |
| rs12916326 | 3.06E-09 | 7.71E-75 | 0.36 | | 0.016 | | 0.739 | |  |
| rs12916648 | 5.56E-09 | 7.82E-75 | 0.42 | | 0.0157 | | 0.721 | |  |
| rs1809420 | 1.27E-09 | 6.72E-73 | 0.387 | | 0.0122 | | 0.564 | |  |
| rs11633351 | 3.20E-09 | 1.51E-73 | 0.389 | | 0.0122 | | 0.585 | |  |
| rs6495267 | 3.01E-09 | 3.04E-73 | 0.355 | | 0.0118 | | 0.589 | |  |
| rs4243085 | 2.26E-08 | 1.16E-23 | 0.945 | | 8.87E-06 | | 0.34 | |  |
| rs2904223 | 2.49E-08 | 6.88E-72 | 0.486 | | 0.00862 | | 0.707 | |  |
| rs8043119 | 1.03E-09 | 8.54E-74 | 0.382 | | 0.00975 | | 0.682 | |  |
| rs1807007 | 2.32E-09 | 5.23E-74 | 0.379 | | 0.00731 | | 0.625 | |  |
| rs1807006 | 1.09E-09 | 3.39E-74 | 0.384 | | 0.00736 | | 0.631 | |  |
| rs1809409 | 2.56E-09 | 2.78E-74 | 0.405 | | 0.00687 | | 0.64 | |  |
| rs11635931 | 4.12E-09 | 3.01E-74 | 0.399 | | 0.00647 | | 0.65 | |  |
| rs11635870 | 4.58E-10 | 2.83E-74 | 0.4 | | 0.00644 | | 0.637 | |  |
| rs7174367 | 4.57E-10 | 7.59E-75 | 0.392 | | 0.00587 | | 0.655 | |  |
| rs57708073 | 3.60E-09 | 9.36E-16 | 0.689 | | 7.07E-05 | | 0.455 | |  |
| rs35934157 | 5.66E-11 | 5.90E-63 | 0.611 | | 0.0149 | | 0.582 | |  |
| rs4887102 | 1.74E-09 | 5.39E-63 | 0.615 | | 0.016 | | 0.519 | |  |
| rs7171578 | 1.80E-10 | 8.34E-63 | 0.6 | | 0.0121 | | 0.493 | |  |
| rs12438645 | 2.37E-10 | 3.97E-15 | 0.696 | | 6.44E-05 | | 0.454 | |  |
| rs12906691 | 1.45E-10 | 1.88E-62 | 0.615 | | 0.0147 | | 0.525 | |  |
| rs11854507 | 2.10E-09 | 2.41E-62 | 0.592 | | 0.0137 | | 0.552 | |  |
| rs7161774 | 7.41E-08 | 3.47E-62 | 0.52 | | 0.0188 | | 0.712 | |  |
| rs62012628 | 4.83E-08 | 5.56E-17 | 0.863 | | 0.000128 | | 0.329 | |  |
| rs62012629 | 6.53E-11 | 9.38E-13 | 0.612 | | 0.000218 | | 0.502 | |  |
| rs12907764 | 2.30E-11 | 1.45E-56 | 0.713 | | 0.0287 | | 0.885 | |  |
| rs12913260 | 3.08E-13 | 1.28E-62 | 0.774 | | 0.0375 | | 0.753 | |  |
| rs2004038 | 5.89E-12 | 3.48E-61 | 0.775 | | 0.0301 | | 0.729 | |  |
| rs59697303 | 6.05E-11 | 1.56E-16 | 0.651 | | 9.17E-05 | | 0.744 | |  |
| rs112321636 | 4.44E-12 | 1.74E-61 | 0.75 | | 0.0262 | | 0.705 | |  |
| rs35474770 | 2.54E-12 | 2.31E-61 | 0.765 | | 0.0266 | | 0.667 | |  |
| rs36061084 | 1.69E-10 | 1.25E-60 | 0.721 | | 0.023 | | 0.634 | |  |
| rs4887109 | 2.22E-15 | 3.24E-61 | 0.846 | | 0.0396 | | 0.728 | |  |
| rs4886590 | 5.36E-13 | 3.86E-62 | 0.753 | | 0.0276 | | 0.683 | |  |
| rs4886591 | 9.03E-11 | 1.41E-72 | 0.505 | | 0.0113 | | 0.696 | |  |
| rs12050525 | 2.13E-12 | 5.58E-59 | 0.873 | | 0.0586 | | 0.9 | |  |
| rs12440925 | 5.83E-09 | 5.70E-10 | 0.365 | | 0.000539 | | 0.709 | |  |
| rs2904220 | 1.12E-12 | 2.12E-58 | 0.867 | | 0.0616 | | 0.939 | |  |
| rs2869862 | 8.91E-12 | 4.15E-16 | 0.69 | | 4.58E-05 | | 0.871 | |  |
| rs1825087 | 7.73E-13 | 7.50E-59 | 0.88 | | 0.0568 | | 0.894 | |  |
| rs11072806 | 4.89E-13 | 7.78E-59 | 0.868 | | 0.0535 | | 0.922 | |  |
| rs12899147 | 2.16E-13 | 2.82E-59 | 0.867 | | 0.0512 | | 0.926 | |  |
| rs1994016 | 2.13E-13 | 6.61E-52 | 0.724 | | 0.045 | | 0.894 | |  |
| rs3743058 | 8.20E-12 | 6.15E-16 | 0.729 | | 4.53E-05 | | 0.903 | |  |
| rs2277547 | 1.71E-11 | 5.03E-16 | 0.723 | | 4.60E-05 | | 0.887 | |  |
| rs4886592 | 4.90E-13 | 1.24E-59 | 0.833 | | 0.0402 | | 0.986 | |  |
| rs2277546 | 2.45E-13 | 9.76E-60 | 0.837 | | 0.0422 | | 0.97 | |  |
| rs2277545 | 2.66E-13 | 1.68E-59 | 0.831 | | 0.0392 | | 0.991 | |  |
| rs11639044 | 2.66E-14 | 8.62E-60 | 0.835 | | 0.044 | | 0.933 | |  |
| rs12438008 | 3.24E-09 | 3.70E-18 | 0.852 | | 0.0013 | | 0.887 | |  |
| rs12903203 | 1.98E-13 | 1.26E-59 | 0.861 | | 0.0473 | | 0.957 | |  |
| rs11631955 | 2.20E-14 | 4.13E-59 | 0.879 | | 0.0454 | | 0.933 | |  |
| rs11632102 | 2.04E-14 | 1.17E-59 | 0.902 | | 0.0479 | | 0.905 | |  |
| rs12437868 | 2.56E-09 | 5.22E-17 | 0.6 | | 0.00303 | | 0.871 | |  |
| rs3825807 | 3.67E-13 | 4.33E-58 | 0.875 | | 0.0846 | | 0.937 | |  |
| rs7177699 | 5.79E-13 | 6.35E-58 | 0.875 | | 0.0793 | | 0.961 | |  |
| rs28610385 | 2.84E-13 | 6.86E-58 | 0.881 | | 0.0844 | | 0.96 | |  |
| rs11638321 | 3.08E-13 | 2.32E-58 | 0.915 | | 0.0803 | | 0.985 | |  |
| rs7173267 | 3.80E-13 | 7.39E-58 | 0.885 | | 0.0905 | | 0.979 | |  |
| rs11634450 | 3.76E-13 | 6.76E-58 | 0.872 | | 0.0866 | | 0.979 | |  |
| rs7169068 | 2.10E-09 | 5.98E-17 | 0.611 | | 0.0033 | | 0.892 | |  |
| rs28691842 | 1.71E-09 | 6.55E-17 | 0.687 | | 0.0061 | | 0.905 | |  |
| rs11856536 | 3.98E-13 | 5.29E-58 | 0.894 | | 0.0894 | | 0.989 | |  |
| rs4887113 | 1.35E-14 | 1.78E-58 | 0.872 | | 0.0835 | | 0.979 | |  |
| rs113458918 | 2.86E-09 | 8.39E-17 | 0.598 | | 0.0034 | | 0.887 | |  |
| rs12592721 | 4.43E-13 | 3.76E-58 | 0.871 | | 0.0954 | | 0.98 | |  |
| rs6495330 | 2.71E-09 | 5.62E-17 | 0.623 | | 0.00367 | | 0.885 | |  |
| rs11634042 | 2.17E-13 | 3.39E-47 | 0.614 | | 0.113 | | 0.857 | |  |
| rs7183257 | 1.23E-09 | 2.28E-09 | 0.756 | | 0.00329 | | 0.903 | |  |
| rs12907065 | 3.45E-13 | 9.77E-35 | 0.84 | | 0.0363 | | 0.837 | |  |
| rs11638020 | 1.88E-13 | 7.35E-35 | 0.991 | | 0.0656 | | 0.804 | |  |
| rs3890715 | 1.00E-12 | 8.35E-35 | 0.993 | | 0.0668 | | 0.743 | |  |
| rs3971829 | 3.68E-12 | 2.12E-34 | 0.98 | | 0.0698 | | 0.75 | |  |
| rs4387568 | 1.40E-14 | 4.81E-34 | 0.997 | | 0.0707 | | 0.834 | |  |
| rs28513584 | 7.70E-13 | 3.16E-34 | 0.99 | | 0.0685 | | 0.815 | |  |
| rs9806363 | 1.69E-14 | 2.87E-34 | 0.978 | | 0.0622 | | 0.83 | |  |
| rs34372090 | 2.64E-14 | 3.05E-34 | 0.987 | | 0.0546 | | 0.822 | |  |
| rs4380028 | 2.53E-14 | 1.48E-33 | 0.954 | | 0.0499 | | 0.886 | |  |
| rs11072807 | 1.20E-11 | 5.27E-34 | 0.991 | | 0.0592 | | 0.843 | |  |
| rs7403593 | 2.42E-14 | 4.83E-34 | 0.996 | | 0.0603 | | 0.842 | |  |
| rs7403728 | 1.74E-14 | 5.42E-34 | 0.995 | | 0.0606 | | 0.84 | |  |
| rs12898392 | 6.73E-12 | 3.56E-34 | 0.994 | | 0.06 | | 0.839 | |  |
| rs56683682 | 3.22E-14 | 3.21E-34 | 0.972 | | 0.0604 | | 0.912 | |  |
| rs12899452 | 1.82E-12 | 3.43E-34 | 0.954 | | 0.0723 | | 0.894 | |  |
| rs12898292 | 2.76E-12 | 9.03E-34 | 0.952 | | 0.063 | | 0.815 | |  |
| rs12898712 | 4.63E-12 | 5.48E-34 | 0.962 | | 0.0687 | | 0.833 | |  |
| rs12898452 | 4.68E-12 | 6.97E-34 | 0.97 | | 0.0646 | | 0.829 | |  |
| rs8027833 | 2.05E-12 | 2.89E-34 | 0.978 | | 0.0695 | | 0.82 | |  |
| rs8026598 | 2.28E-12 | 4.19E-34 | 0.982 | | 0.0668 | | 0.83 | |  |
| rs6495333 | 2.21E-12 | 3.24E-34 | 0.972 | | 0.0647 | | 0.819 | |  |
| rs7166340 | 7.48E-12 | 7.15E-35 | 0.987 | | 0.0693 | | 0.851 | |  |
| rs7167853 | 4.93E-12 | 2.24E-24 | 0.996 | | 0.0711 | | 0.873 | |  |
| rs7167056 | 9.69E-12 | 1.79E-34 | 0.961 | | 0.0691 | | 0.848 | |  |
| rs7167242 | 1.30E-11 | 2.62E-34 | 0.938 | | 0.0723 | | 0.915 | |  |
| rs7166770 | 1.12E-11 | 4.63E-34 | 0.963 | | 0.0589 | | 0.869 | |  |
| rs7402376 | 2.10E-14 | 3.14E-34 | 0.933 | | 0.0651 | | 0.973 | |  |
| rs9920493 | 2.22E-12 | 2.47E-34 | 0.991 | | 0.0657 | | 0.785 | |  |
| rs12909317 | 1.53E-14 | 1.78E-34 | 0.986 | | 0.0547 | | 0.852 | |  |
| rs28455815 | 3.63E-14 | 1.49E-36 | 0.987 | | 0.0642 | | 0.967 | |  |
| rs28505515 | 7.30E-13 | 1.10E-29 | 0.94 | | 0.109 | | 0.86 | |  |
| rs56076311 | 1.58E-14 | 5.27E-35 | 0.944 | | 0.0335 | | 0.817 | |  |
| rs8041064 | 3.36E-12 | 2.40E-34 | 0.999 | | 0.0568 | | 0.79 | |  |
| rs66925868 | 1.96E-12 | 2.47E-34 | 0.988 | | 0.049 | | 0.869 | |  |
| rs7403608 | 2.20E-14 | 2.58E-34 | 0.971 | | 0.055 | | 0.858 | |  |
| rs12915192 | 3.93E-12 | 2.13E-34 | 0.968 | | 0.0548 | | 0.808 | |  |
| rs7165902 | 3.02E-12 | 2.42E-34 | 0.992 | | 0.0601 | | 0.813 | |  |
| rs7165075 | 2.91E-12 | 2.05E-34 | 0.988 | | 0.0597 | | 0.804 | |  |
| rs6495335 | 2.58E-12 | 2.73E-34 | 0.987 | | 0.0647 | | 0.777 | |  |
| rs8032908 | 1.76E-12 | 1.30E-29 | 0.92 | | 0.101 | | 0.865 | |  |
| rs12442476 | 2.36E-12 | 2.40E-34 | 0.982 | | 0.0614 | | 0.816 | |  |
| rs55870734 | 2.00E-14 | 2.24E-34 | 0.983 | | 0.0596 | | 0.879 | |  |
| rs7178051 | 1.89E-14 | 2.98E-34 | 0.991 | | 0.065 | | 0.85 | |  |
| rs7403714 | 2.16E-14 | 3.50E-34 | 0.99 | | 0.0597 | | 0.857 | |  |
| rs9920968 | 4.74E-12 | 3.21E-34 | 0.987 | | 0.0673 | | 0.788 | |  |
| rs58021857 | 1.38E-14 | 2.82E-34 | 0.98 | | 0.0647 | | 0.842 | |  |
| rs61043060 | 1.60E-14 | 3.36E-34 | 0.906 | | 0.0783 | | 0.892 | |  |
| rs62011001 | 2.44E-11 | 2.83E-24 | 0.919 | | 0.0803 | | 0.861 | |  |
| rs62011002 | 1.51E-12 | 2.63E-34 | 0.92 | | 0.0858 | | 0.822 | |  |
| rs8035093 | 1.53E-12 | 2.66E-34 | 0.921 | | 0.0862 | | 0.82 | |  |
| rs7176187 | 1.23E-12 | 3.34E-34 | 0.978 | | 0.0679 | | 0.775 | |  |
| rs4389113 | 1.64E-14 | 2.94E-34 | 0.959 | | 0.0608 | | 0.855 | |  |
| rs7179953 | 2.22E-15 | 3.11E-36 | 0.971 | | 0.0722 | | 0.974 | |  |
| rs4433781 | 1.12E-14 | 3.43E-34 | 0.982 | | 0.0657 | | 0.835 | |  |
| rs4389112 | 1.07E-12 | 3.50E-34 | 0.987 | | 0.0624 | | 0.846 | |  |
| rs4073322 | 1.12E-12 | 4.18E-34 | 0.97 | | 0.0693 | | 0.765 | |  |
| rs72749206 | 9.51E-13 | 3.11E-34 | 0.985 | | 0.0588 | | 0.845 | |  |
| rs7164479 | 8.88E-16 | 3.78E-36 | 0.906 | | 0.0833 | | 0.921 | |  |
| rs7165042 | 6.66E-16 | 4.35E-36 | 0.978 | | 0.0699 | | 0.958 | |  |
| rs7165081 | 7.77E-16 | 3.94E-36 | 0.989 | | 0.0743 | | 0.932 | |  |
| rs7165733 | 7.77E-16 | 3.53E-36 | 0.926 | | 0.0733 | | 0.943 | |  |
| rs7166764 | 8.88E-16 | 3.77E-36 | 0.93 | | 0.0799 | | 0.947 | |  |
| rs7182529 | 6.66E-16 | 1.85E-36 | 0.947 | | 0.0728 | | 0.985 | |  |
| rs7182716 | 6.66E-16 | 1.97E-36 | 0.947 | | 0.0727 | | 0.98 | |  |
| rs7181432 | 6.66E-16 | 2.07E-36 | 0.942 | | 0.0727 | | 0.977 | |  |
| rs7403708 | 6.77E-15 | 3.15E-34 | 0.946 | | 0.0613 | | 0.837 | |  |
| rs7182103 | 5.55E-16 | 3.56E-36 | 0.922 | | 0.0679 | | 0.952 | |  |
| rs10083696 | 7.77E-15 | 4.82E-34 | 0.93 | | 0.0625 | | 0.868 | |  |
| rs10083697 | 8.77E-15 | 4.88E-34 | 0.933 | | 0.0626 | | 0.875 | |  |
| rs4468572 | 4.44E-16 | 3.20E-36 | 0.908 | | 0.0606 | | 0.981 | |  |
| rs67745769 | 3.55E-15 | 5.89E-35 | 0.886 | | 0.0519 | | 0.794 | |  |
| rs11852830 | 2.18E-11 | 9.63E-38 | 0.704 | | 0.125 | | 0.727 | |  |
| rs6495337 | 1.21E-11 | 5.08E-27 | 0.688 | | 0.197 | | 0.946 | |  |
| rs8032842 | 8.53E-12 | 7.50E-37 | 0.755 | | 0.0667 | | 0.719 | |  |
| rs8032771 | 8.35E-12 | 7.45E-37 | 0.73 | | 0.0622 | | 0.735 | |  |
| rs8034274 | 1.12E-10 | 8.59E-42 | 0.769 | | 0.0545 | | 0.823 | |  |
| rs62011004 | 5.88E-14 | 1.02E-36 | 0.685 | | 0.0635 | | 0.706 | |  |
| rs12908341 | 1.15E-13 | 1.03E-36 | 0.687 | | 0.064 | | 0.705 | |  |
| rs7178750 | 8.17E-12 | 8.25E-37 | 0.667 | | 0.0609 | | 0.707 | |  |
| rs8024048 | 1.43E-11 | 6.14E-37 | 0.739 | | 0.0673 | | 0.71 | |  |
| rs6495338 | 7.74E-12 | 8.69E-18 | 0.522 | | 0.937 | | 0.964 | |  |
| rs6495339 | 9.43E-12 | 6.76E-37 | 0.734 | | 0.064 | | 0.707 | |  |
| rs6495340 | 9.68E-12 | 7.26E-37 | 0.725 | | 0.0635 | | 0.709 | |  |
| rs4539564 | 9.92E-12 | 6.49E-37 | 0.734 | | 0.0637 | | 0.712 | |  |
| rs7169511 | 1.01E-11 | 8.27E-37 | 0.734 | | 0.0639 | | 0.714 | |  |
| rs7168915 | 2.78E-10 | 1.05E-41 | 0.772 | | 0.0555 | | 0.813 | |  |
| rs12903613 | 1.87E-09 | 6.22E-37 | 0.702 | | 0.0603 | | 0.746 | |  |
| rs12903668 | 2.01E-09 | 1.02E-36 | 0.725 | | 0.0508 | | 0.743 | |  |
| rs8030937 | 2.69E-12 | 5.75E-37 | 0.726 | | 0.0645 | | 0.729 | |  |
| rs8034804 | 2.04E-09 | 7.59E-37 | 0.712 | | 0.0528 | | 0.751 | |  |
| rs8035039 | 1.69E-09 | 1.11E-36 | 0.711 | | 0.0585 | | 0.73 | |  |
| rs7403155 | 4.55E-09 | 1.41E-31 | 0.757 | | 0.141 | | 0.855 | |  |
| rs4551997 | 1.43E-11 | 1.27E-36 | 0.712 | | 0.0525 | | 0.748 | |  |
| rs11072810 | 2.45E-12 | 7.59E-33 | 0.814 | | 0.152 | | 0.659 | |  |
| rs11072811 | 4.68E-10 | 5.67E-33 | 0.818 | | 0.145 | | 0.683 | |  |
| rs11632963 | 4.91E-12 | 7.15E-33 | 0.794 | | 0.154 | | 0.634 | |  |
| rs62011027 | 2.30E-08 | 0.00564 | 0.658 | | 0.385 | | 0.71 | |  |
| rs28694044 | 3.17E-13 | 5.67E-37 | 0.914 | | 0.0795 | | 0.744 | |  |
| rs28580532 | 2.33E-13 | 5.60E-37 | 0.913 | | 0.0802 | | 0.747 | |  |
| rs12442036 | 2.03E-08 | 0.00688 | 0.705 | | 0.337 | | 0.668 | |  |
| rs56227704 | 7.74E-12 | 5.78E-33 | 0.948 | | 0.119 | | 0.43 | |  |
| rs12232282 | 2.98E-13 | 2.30E-37 | 0.951 | | 0.0753 | | 0.692 | |  |
| rs4420501 | 4.91E-13 | 2.49E-37 | 0.973 | | 0.079 | | 0.691 | |  |
| rs4438276 | 2.39E-13 | 2.56E-37 | 0.962 | | 0.0752 | | 0.697 | |  |
| rs4439728 | 4.71E-13 | 2.22E-37 | 0.968 | | 0.0832 | | 0.69 | |  |
| rs62011029 | 7.45E-09 | 0.00601 | 0.709 | | 0.352 | | 0.712 | |  |
| rs11637783 | 5.92E-14 | 3.27E-37 | 0.952 | | 0.0741 | | 0.702 | |  |
| rs8037171 | 1.08E-12 | 2.70E-37 | 0.971 | | 0.0866 | | 0.699 | |  |
| rs56031815 | 3.86E-09 | 0.00598 | 0.712 | | 0.354 | | 0.717 | |  |
| rs62011032 | 7.03E-09 | 0.00653 | 0.718 | | 0.353 | | 0.713 | |  |
| rs62011033 | 7.09E-09 | 0.00634 | 0.726 | | 0.346 | | 0.724 | |  |
| rs11072812 | 4.71E-13 | 5.37E-37 | 0.967 | | 0.0828 | | 0.706 | |  |
| rs62011034 | 7.52E-09 | 0.0068 | 0.726 | | 0.354 | | 0.727 | |  |
| rs4567668 | 4.81E-13 | 6.52E-37 | 0.976 | | 0.0797 | | 0.691 | |  |
| rs11857877 | 5.94E-13 | 1.15E-37 | 0.951 | | 0.0667 | | 0.667 | |  |
| rs4628930 | 7.00E-09 | 0.00716 | 0.724 | | 0.354 | | 0.726 | |  |
| rs4132786 | 3.60E-13 | 1.00E-36 | 0.976 | | 0.0731 | | 0.711 | |  |
| rs4344704 | 6.66E-16 | 1.15E-36 | 0.961 | | 0.0995 | | 0.704 | |  |
| rs7173743 | 5.55E-16 | 1.17E-36 | 0.971 | | 0.102 | | 0.699 | |  |
| rs147228279 | 6.70E-09 | 0.0066 | 0.746 | | 0.352 | | 0.723 | |  |
| rs11639335 | 1.88E-10 | 1.23E-26 | 0.601 | | 0.385 | | 0.397 | |  |
| rs11632020 | 3.23E-13 | 7.70E-37 | 0.973 | | 0.0803 | | 0.712 | |  |
| rs12440662 | 6.10E-09 | 0.00682 | 0.693 | | 0.302 | | 0.751 | |  |
| rs34184420 | 4.63E-13 | 5.34E-35 | 0.906 | | 0.0905 | | 0.7 | |  |
| rs12911870 | 2.05E-13 | 1.09E-35 | 0.796 | | 0.0696 | | 0.638 | |  |
| rs62011036 | 3.46E-08 | 0.00387 | 0.917 | | 0.239 | | 0.705 | |  |
| rs76247098 | 2.70E-08 | 0.00401 | 0.933 | | 0.241 | | 0.706 | |  |
| rs12903249 | 4.39E-13 | 2.42E-35 | 0.806 | | 0.068 | | 0.662 | |  |
| rs12903542 | 4.25E-13 | 2.83E-35 | 0.8 | | 0.07 | | 0.641 | |  |
| rs75023556 | 3.41E-08 | 0.00434 | 0.985 | | 0.256 | | 0.713 | |  |
| rs5029904 | 4.27E-13 | 2.25E-35 | 0.822 | | 0.066 | | 0.654 | |  |
| rs12442152 | 7.66E-08 | 0.00405 | 0.954 | | 0.254 | | 0.721 | |  |
| rs62011050 | 8.05E-08 | 0.00422 | 0.944 | | 0.259 | | 0.702 | |  |
| rs61157089 | 9.04E-08 | 0.00406 | 0.984 | | 0.276 | | 0.677 | |  |
| rs4491476 | 2.86E-10 | 8.45E-25 | 0.504 | | 0.417 | | 0.378 | |  |
| rs11632720 | 2.36E-10 | 7.35E-26 | 0.519 | | 0.305 | | 0.343 | |  |
| rs1807214 | 5.11E-08 | 0.237 | 0.386 | | 0.83 | | 0.796 | |  |
| rs745386 | 7.54E-08 | 0.247 | 0.399 | | 0.747 | | 0.76 | |  |
| rs8023801 | 9.49E-08 | 0.22 | 0.376 | | 0.821 | | 0.815 | |  |
| rs8042271 | 3.68E-08 | 0.769 | 0.882 | | 0.926 | | 0.984 | |  |
| rs2083457 | 9.67E-08 | 0.214 | 0.414 | | 0.734 | | 0.689 | |  |
| rs2083460 | 6.93E-08 | 0.215 | 0.377 | | 0.724 | | 0.736 | |  |
| rs7164299 | 9.17E-08 | 0.22 | 0.434 | | 0.743 | | 0.717 | |  |
| rs2071382 | 8.80E-08 | 0.103 | 0.106 | | 0.745 | | 0.164 | |  |
| rs2521501 | 5.01E-08 | 0.944 | 0.0265 | | 0.445 | | 0.77 | |  |
| rs66723169 | 7.54E-08 | 0.000222 | 0.366 | | 0.148 | | 3.21E-06 | |  |
| rs6567160 | 4.56E-08 | 0.00039 | 0.57 | | 0.281 | | 1.78E-06 | |  |
| rs663129 | 3.20E-08 | 0.000239 | 0.551 | | 0.25 | | 1.10E-06 | |  |
| rs571312 | 3.88E-08 | 0.000261 | 0.549 | | 0.252 | | 1.08E-06 | |  |
| rs523288 | 6.29E-08 | 0.00029 | 0.48 | | 0.217 | | 9.17E-07 | |  |
| rs538656 | 8.29E-08 | 0.000245 | 0.485 | | 0.206 | | 9.90E-07 | |  |
| rs11152213 | 9.52E-08 | 0.000221 | 0.63 | | 0.132 | | 2.71E-06 | |  |
| rs34633411 | 5.07E-08 | 0.000265 | 0.533 | | 0.128 | | 2.41E-06 | |  |
| rs12958167 | 7.14E-08 | 0.000388 | 0.274 | | 0.175 | | 7.75E-06 | |  |
| rs12954782 | 7.58E-08 | 0.00034 | 0.262 | | 0.18 | | 9.44E-06 | |  |
| rs1942872 | 9.45E-08 | 0.000715 | 0.286 | | 0.189 | | 5.72E-06 | |  |
| rs12955983 | 7.38E-08 | 0.000657 | 0.283 | | 0.201 | | 9.38E-06 | |  |
| rs11663816 | 5.02E-08 | 0.000766 | 0.253 | | 0.209 | | 9.95E-06 | |  |
| rs11664883 | 9.73E-08 | 0.000715 | 0.263 | | 0.215 | | 1.04E-05 | |  |
| rs2045438 | 7.37E-08 | 0.000674 | 0.265 | | 0.225 | | 1.06E-05 | |  |
| rs17175602 | 8.39E-08 | 0.000736 | 0.254 | | 0.224 | | 1.15E-05 | |  |
| rs2045439 | 6.48E-08 | 0.00071 | 0.252 | | 0.224 | | 1.19E-05 | |  |
| rs12957325 | 8.15E-08 | 0.000765 | 0.253 | | 0.228 | | 1.34E-05 | |  |
| rs12970134 | 9.09E-08 | 0.00075 | 0.236 | | 0.232 | | 1.40E-05 | |  |
| rs11660069 | 6.79E-08 | 0.00072 | 0.255 | | 0.229 | | 1.23E-05 | |  |
| rs35476226 | 6.77E-08 | 0.000754 | 0.264 | | 0.226 | | 1.13E-05 | |  |
| rs67713315 | 9.54E-08 | 0.00109 | 0.274 | | 0.239 | | 1.43E-05 | |  |
| rs67194783 | 7.48E-08 | 0.00102 | 0.308 | | 0.262 | | 1.55E-05 | |  |
| rs17175643 | 9.01E-08 | 0.00121 | 0.322 | | 0.241 | | 1.76E-05 | |  |
| rs12956871 | 8.50E-08 | 0.000992 | 0.261 | | 0.235 | | 1.50E-05 | |  |
| rs12960928 | 9.42E-08 | 0.00104 | 0.276 | | 0.24 | | 1.78E-05 | |  |
| rs11665052 | 8.45E-08 | 0.00182 | 0.284 | | 0.257 | | 1.99E-05 | |  |
| rs12966550 | 8.57E-08 | 0.00189 | 0.275 | | 0.271 | | 1.95E-05 | |  |
| rs117786851 | 8.07E-08 | 0.0231 | 0.721 | | 0.00753 | | 0.316 | |  |
| rs11880613 | 9.61E-08 | 0.0249 | 0.667 | | 0.00626 | | 0.394 | |  |
| rs1109375 | 4.31E-08 | 0.0157 | 0.702 | | 0.0145 | | 0.567 | |  |
| rs8111962 | 4.69E-08 | 0.0156 | 0.693 | | 0.0144 | | 0.561 | |  |
| rs2287029 | 3.04E-08 | 0.0164 | 0.688 | | 0.0146 | | 0.599 | |  |
| rs117159625 | 7.43E-08 | 0.000179 | 0.757 | | 0.293 | | 0.993 | |  |
| rs73007593 | 7.65E-08 | 0.000752 | 0.704 | | 0.176 | | 0.68 | |  |
| rs11881156 | 8.47E-08 | 0.0045 | 0.78 | | 0.0276 | | 0.983 | |  |
| rs11881438 | 1.40E-08 | 0.0145 | 0.885 | | 0.107 | | 0.91 | |  |
| rs12973042 | 8.14E-10 | 0.0107 | 0.642 | | 0.0856 | | 0.757 | |  |
| rs12979495 | 4.73E-10 | 0.012 | 0.624 | | 0.0925 | | 0.746 | |  |
| rs67987899 | 1.50E-08 | 0.0125 | 0.865 | | 0.116 | | 0.871 | |  |
| rs35443547 | 3.20E-08 | 0.0165 | 0.81 | | 0.101 | | 0.824 | |  |
| rs7260254 | 2.33E-08 | 0.0155 | 0.825 | | 0.107 | | 0.823 | |  |
| rs7256879 | 2.27E-08 | 0.0139 | 0.786 | | 0.102 | | 0.86 | |  |
| rs1971038 | 1.73E-08 | 0.0154 | 0.826 | | 0.107 | | 0.873 | |  |
| rs1971039 | 2.18E-08 | 0.0156 | 0.811 | | 0.102 | | 0.858 | |  |
| rs1541595 | 2.18E-08 | 0.0155 | 0.817 | | 0.1 | | 0.853 | |  |
| rs11880628 | 8.36E-10 | 0.0116 | 0.571 | | 0.0906 | | 0.696 | |  |
| rs12977506 | 1.09E-09 | 0.0112 | 0.564 | | 0.089 | | 0.696 | |  |
| rs36045835 | 7.62E-10 | 0.0123 | 0.563 | | 0.0887 | | 0.676 | |  |
| rs73009538 | 9.11E-10 | 0.0114 | 0.565 | | 0.0914 | | 0.685 | |  |
| rs35734575 | 9.38E-10 | 0.0116 | 0.568 | | 0.0917 | | 0.689 | |  |
| rs11879571 | 1.53E-09 | 0.0118 | 0.585 | | 0.0921 | | 0.684 | |  |
| rs4804142 | 1.99E-09 | 0.0114 | 0.59 | | 0.0959 | | 0.681 | |  |
| rs8105092 | 1.72E-08 | 0.0154 | 0.831 | | 0.103 | | 0.795 | |  |
| rs74179956 | 1.02E-09 | 0.01 | 0.594 | | 0.092 | | 0.684 | |  |
| rs34857893 | 2.26E-08 | 0.0147 | 0.823 | | 0.111 | | 0.786 | |  |
| rs12971616 | 2.00E-09 | 0.00868 | 0.576 | | 0.0945 | | 0.626 | |  |
| rs12976693 | 1.49E-09 | 0.00949 | 0.574 | | 0.0887 | | 0.626 | |  |
| rs17616105 | 2.46E-09 | 0.00933 | 0.56 | | 0.0962 | | 0.633 | |  |
| rs2304089 | 4.45E-08 | 0.0175 | 0.887 | | 0.0943 | | 0.856 | |  |
| rs35991287 | 2.39E-09 | 0.0099 | 0.652 | | 0.0888 | | 0.67 | |  |
| rs2053065 | 5.06E-08 | 0.0154 | 0.926 | | 0.107 | | 0.856 | |  |
| rs2053064 | 4.19E-08 | 0.0149 | 0.884 | | 0.102 | | 0.869 | |  |
| rs4804547 | 5.61E-09 | 0.00889 | 0.621 | | 0.102 | | 0.661 | |  |
| rs17850995 | 4.81E-08 | 8.19E-05 | 0.902 | | 0.298 | | 0.956 | |  |
| rs4804548 | 9.60E-09 | 0.0209 | 0.857 | | 0.087 | | 0.954 | |  |
| rs74179958 | 2.16E-09 | 0.0116 | 0.685 | | 0.157 | | 0.696 | |  |
| rs8106381 | 1.54E-09 | 0.0115 | 0.702 | | 0.0887 | | 0.7 | |  |
| rs113528897 | 1.09E-08 | 0.0209 | 0.885 | | 0.0836 | | 0.894 | |  |
| rs35887384 | 2.31E-09 | 0.0116 | 0.705 | | 0.091 | | 0.674 | |  |
| rs34370923 | 1.71E-09 | 0.012 | 0.71 | | 0.0856 | | 0.71 | |  |
| rs35479557 | 2.15E-09 | 0.0112 | 0.706 | | 0.0884 | | 0.706 | |  |
| rs35018144 | 1.54E-08 | 0.0203 | 0.864 | | 0.0893 | | 0.915 | |  |
| rs34837972 | 4.62E-09 | 0.0116 | 0.694 | | 0.0873 | | 0.696 | |  |
| rs34677258 | 2.24E-09 | 0.0117 | 0.699 | | 0.0873 | | 0.702 | |  |
| rs74179959 | 8.39E-09 | 0.0196 | 0.863 | | 0.0928 | | 0.948 | |  |
| rs35568987 | 1.22E-09 | 0.0244 | 0.703 | | 0.0927 | | 0.551 | |  |
| rs7257769 | 4.82E-09 | 0.0133 | 0.692 | | 0.0804 | | 0.672 | |  |
| rs8101254 | 5.02E-09 | 0.0147 | 0.697 | | 0.0832 | | 0.734 | |  |
| rs11085750 | 2.04E-08 | 0.0215 | 0.856 | | 0.0852 | | 0.906 | |  |
| rs56335280 | 1.58E-08 | 0.0288 | 0.289 | | 0.177 | | 0.365 | |  |
| rs12611283 | 3.55E-09 | 0.0313 | 0.358 | | 0.147 | | 0.538 | |  |
| rs73011343 | 7.68E-08 | 0.0553 | 0.403 | | 0.148 | | 0.839 | |  |
| rs112334024 | 1.40E-08 | 0.0372 | 0.292 | | 0.135 | | 0.62 | |  |
| rs35140030 | 4.97E-08 | 0.0611 | 0.403 | | 0.126 | | 0.856 | |  |
| rs12611017 | 1.77E-08 | 0.0341 | 0.236 | | 0.145 | | 0.61 | |  |
| rs113680403 | 2.27E-08 | 0.0353 | 0.217 | | 0.145 | | 0.617 | |  |
| rs56405759 | 1.41E-08 | 0.0373 | 0.234 | | 0.16 | | 0.664 | |  |
| rs113718993 | 7.90E-09 | 0.0363 | 0.216 | | 0.172 | | 0.723 | |  |
| rs112651246 | 1.69E-08 | 0.0388 | 0.233 | | 0.146 | | 0.605 | |  |
| rs111380365 | 1.20E-08 | 0.0464 | 0.246 | | 0.132 | | 0.71 | |  |
| rs12610374 | 2.29E-09 | 0.0859 | 0.317 | | 0.114 | | 0.78 | |  |
| rs4804553 | 1.26E-08 | 0.0449 | 0.256 | | 0.128 | | 0.698 | |  |
| rs73011374 | 1.07E-08 | 0.0468 | 0.257 | | 0.131 | | 0.709 | |  |
| rs4804554 | 1.65E-08 | 0.0443 | 0.276 | | 0.147 | | 0.707 | |  |
| rs12610607 | 1.16E-08 | 0.0428 | 0.283 | | 0.148 | | 0.721 | |  |
| rs4804557 | 2.58E-09 | 0.087 | 0.312 | | 0.121 | | 0.803 | |  |
| rs56385993 | 1.36E-08 | 0.0477 | 0.314 | | 0.149 | | 0.79 | |  |
| rs2288844 | 3.08E-09 | 0.0577 | 0.264 | | 0.145 | | 0.772 | |  |
| rs17766161 | 4.08E-09 | 0.0592 | 0.268 | | 0.147 | | 0.746 | |  |
| rs56122482 | 6.19E-09 | 0.119 | 0.296 | | 0.129 | | 0.824 | |  |
| rs73013159 | 2.35E-08 | 0.00283 | 0.583 | | 0.453 | | 0.756 | |  |
| rs73013166 | 2.25E-08 | 0.00422 | 0.684 | | 0.527 | | 0.715 | |  |
| rs12611191 | 4.38E-09 | 0.0909 | 0.266 | | 0.138 | | 0.663 | |  |
| rs1019193 | 2.08E-08 | 0.0465 | 0.234 | | 0.16 | | 0.652 | |  |
| rs1019194 | 1.95E-08 | 0.0499 | 0.243 | | 0.164 | | 0.632 | |  |
| rs4804561 | 2.18E-08 | 0.0517 | 0.23 | | 0.158 | | 0.627 | |  |
| rs881227 | 1.66E-09 | 0.0914 | 0.157 | | 0.233 | | 0.787 | |  |
| rs12052200 | 5.17E-08 | 0.0576 | 0.00888 | | 0.982 | | 0.0571 | |  |
| rs12052201 | 6.73E-10 | 0.337 | 0.0205 | | 0.161 | | 0.542 | |  |
| rs12052058 | 1.31E-10 | 0.327 | 0.0276 | | 0.175 | | 0.586 | |  |
| rs55948246 | 8.86E-11 | 0.332 | 0.0235 | | 0.159 | | 0.623 | |  |
| rs3786722 | 3.76E-11 | 0.374 | 0.0311 | | 0.148 | | 0.605 | |  |
| rs1122608 | 2.73E-11 | 0.503 | 0.0232 | | 0.134 | | 0.564 | |  |
| rs10417578 | 3.14E-08 | 0.367 | 0.00364 | | 0.11 | | 0.416 | |  |
| rs112369586 | 2.55E-11 | 0.476 | 0.0278 | | 0.133 | | 0.599 | |  |
| rs8108935 | 3.12E-08 | 0.347 | 0.0044 | | 0.122 | | 0.4 | |  |
| rs6511718 | 3.57E-08 | 0.311 | 0.00384 | | 0.109 | | 0.408 | |  |
| rs60314748 | 8.27E-08 | 0.265 | 0.00222 | | 0.138 | | 0.131 | |  |
| rs55677033 | 5.34E-08 | 0.266 | 0.0029 | | 0.112 | | 0.196 | |  |
| rs3786723 | 1.83E-08 | 0.377 | 0.00415 | | 0.132 | | 0.323 | |  |
| rs3786725 | 1.92E-09 | 0.354 | 0.00356 | | 0.133 | | 0.381 | |  |
| rs12609863 | 1.35E-11 | 0.535 | 0.0261 | | 0.129 | | 0.5 | |  |
| rs3786727 | 1.78E-08 | 0.334 | 0.00321 | | 0.117 | | 0.54 | |  |
| rs3786728 | 1.62E-11 | 0.483 | 0.0204 | | 0.128 | | 0.7 | |  |
| rs112186070 | 1.78E-11 | 0.527 | 0.0243 | | 0.127 | | 0.501 | |  |
| rs60448955 | 1.89E-08 | 0.366 | 0.00252 | | 0.158 | | 0.463 | |  |
| rs7275 | 6.03E-08 | 0.302 | 0.00284 | | 0.125 | | 0.556 | |  |
| rs11670205 | 4.17E-08 | 0.343 | 0.00354 | | 0.133 | | 0.537 | |  |
| rs68010235 | 6.48E-09 | 0.369 | 0.00378 | | 0.141 | | 0.533 | |  |
| rs11666990 | 6.46E-09 | 0.365 | 0.00512 | | 0.13 | | 0.394 | |  |
| rs11668364 | 7.19E-09 | 0.409 | 0.00525 | | 0.119 | | 0.375 | |  |
| rs11673439 | 8.09E-09 | 0.405 | 0.00512 | | 0.125 | | 0.38 | |  |
| rs12609500 | 4.05E-11 | 0.513 | 0.028 | | 0.139 | | 0.528 | |  |
| rs12609502 | 4.65E-09 | 0.392 | 0.00496 | | 0.135 | | 0.375 | |  |
| rs73013198 | 1.93E-11 | 0.513 | 0.0283 | | 0.13 | | 0.506 | |  |
| rs8103309 | 1.39E-08 | 0.307 | 0.00218 | | 0.164 | | 0.198 | |  |
| rs11666925 | 5.26E-08 | 0.312 | 0.00339 | | 0.159 | | 0.545 | |  |
| rs11085757 | 3.56E-08 | 0.384 | 0.00467 | | 0.129 | | 0.362 | |  |
| rs876794 | 1.06E-08 | 0.366 | 0.00401 | | 0.14 | | 0.503 | |  |
| rs10409001 | 5.30E-08 | 0.36 | 0.00413 | | 0.14 | | 0.511 | |  |
| rs10415811 | 7.08E-08 | 0.373 | 0.00424 | | 0.14 | | 0.504 | |  |
| rs11879026 | 3.84E-08 | 0.393 | 0.00596 | | 0.126 | | 0.37 | |  |
| rs73013202 | 6.47E-12 | 0.513 | 0.0271 | | 0.15 | | 0.657 | |  |
| rs8102273 | 1.68E-08 | 0.379 | 0.00494 | | 0.15 | | 0.509 | |  |
| rs113113862 | 1.76E-12 | 0.591 | 0.0437 | | 0.143 | | 0.548 | |  |
| rs73015007 | 1.04E-12 | 0.551 | 0.0398 | | 0.149 | | 0.376 | |  |
| rs9305019 | 4.77E-08 | 0.15 | 0.789 | | 0.087 | | 0.38 | |  |
| rs143020224 | 5.88E-13 | 0.0528 | 0.544 | | 0.0573 | | 0.907 | |  |
| rs144826254 | 2.84E-14 | 0.043 | 0.562 | | 0.0573 | | 0.888 | |  |
| rs112736558 | 6.04E-13 | 0.0564 | 0.595 | | 0.0587 | | 0.891 | |  |
| rs111989435 | 1.68E-13 | 0.053 | 0.552 | | 0.0574 | | 0.914 | |  |
| rs55997232 | 5.11E-15 | 0.0335 | 0.583 | | 0.0489 | | 0.879 | |  |
| rs55791371 | 4.66E-15 | 0.0333 | 0.617 | | 0.0575 | | 0.773 | |  |
| rs56125973 | 1.89E-14 | 0.038 | 0.617 | | 0.0566 | | 0.842 | |  |
| rs56289821 | 4.44E-15 | 0.0335 | 0.639 | | 0.0606 | | 0.753 | |  |
| rs112898275 | 2.84E-14 | 0.0455 | 0.58 | | 0.055 | | 0.834 | |  |
| rs112374545 | 6.11E-15 | 0.0413 | 0.62 | | 0.0572 | | 0.84 | |  |
| rs148898583 | 5.11E-15 | 0.0338 | 0.626 | | 0.0587 | | 0.771 | |  |
| rs113722226 | 2.69E-14 | 0.0434 | 0.53 | | 0.0627 | | 0.811 | |  |
| rs73015011 | 7.82E-14 | 0.0428 | 0.607 | | 0.0539 | | 0.856 | |  |
| rs114821903 | 6.80E-13 | 0.0469 | 0.598 | | 0.0539 | | 0.87 | |  |
| rs138175288 | 3.26E-13 | 0.0458 | 0.598 | | 0.0536 | | 0.879 | |  |
| rs112107114 | 3.08E-13 | 0.0487 | 0.614 | | 0.0569 | | 0.869 | |  |
| rs115594766 | 3.53E-13 | 0.0425 | 0.625 | | 0.059 | | 0.852 | |  |
| rs112032422 | 2.29E-14 | 0.033 | 0.647 | | 0.0574 | | 0.792 | |  |
| rs142158911 | 2.00E-12 | 0.077 | 0.765 | | 0.146 | | 0.72 | |  |
| rs142130958 | 7.15E-14 | 0.0361 | 0.651 | | 0.0607 | | 0.82 | |  |
| rs73015013 | 8.35E-14 | 0.0446 | 0.623 | | 0.0605 | | 0.854 | |  |
| rs73015016 | 3.63E-12 | 0.0474 | 0.599 | | 0.0461 | | 0.837 | |  |
| rs10402112 | 8.63E-12 | 0.0458 | 0.613 | | 0.0508 | | 0.755 | |  |
| rs138294113 | 1.69E-14 | 0.0356 | 0.647 | | 0.0615 | | 0.84 | |  |
| rs61194703 | 3.88E-12 | 0.0415 | 0.623 | | 0.0559 | | 0.793 | |  |
| rs73015020 | 1.11E-11 | 0.044 | 0.625 | | 0.0559 | | 0.774 | |  |
| rs77140532 | 1.31E-11 | 0.0538 | 0.607 | | 0.0489 | | 0.806 | |  |
| rs73015021 | 4.64E-11 | 0.0524 | 0.584 | | 0.0471 | | 0.786 | |  |
| rs112552009 | 5.73E-14 | 0.0323 | 0.714 | | 0.0627 | | 0.758 | |  |
| rs10412048 | 4.45E-11 | 0.0633 | 0.565 | | 0.0471 | | 0.721 | |  |
| rs8106503 | 8.45E-11 | 0.051 | 0.519 | | 0.105 | | 0.849 | |  |
| rs12151108 | 1.59E-13 | 0.0356 | 0.636 | | 0.0664 | | 0.84 | |  |
| rs73015024 | 2.89E-14 | 0.0372 | 0.639 | | 0.0669 | | 0.811 | |  |
| rs17248720 | 8.91E-12 | 0.0529 | 0.507 | | 0.0914 | | 0.836 | |  |
| rs17248727 | 8.55E-14 | 0.0429 | 0.654 | | 0.0758 | | 0.752 | |  |
| rs6511720 | 1.42E-13 | 0.04 | 0.727 | | 0.0739 | | 0.783 | |  |
| rs6511721 | 3.10E-08 | 0.938 | 0.0648 | | 0.371 | | 0.508 | |  |
| rs17242381 | 1.87E-11 | 0.0684 | 0.55 | | 0.228 | | 0.538 | |  |
| rs17242395 | 1.93E-11 | 0.0679 | 0.477 | | 0.256 | | 0.543 | |  |
| rs117423069 | 2.41E-11 | 0.0725 | 0.528 | | 0.22 | | 0.572 | |  |
| rs17248769 | 3.37E-11 | 0.0907 | 0.471 | | 0.266 | | 0.552 | |  |
| rs73015033 | 3.38E-11 | 0.0966 | 0.476 | | 0.247 | | 0.573 | |  |
| rs73015034 | 3.94E-11 | 0.103 | 0.474 | | 0.294 | | 0.586 | |  |
| rs74857287 | 3.67E-11 | 0.109 | 0.489 | | 0.286 | | 0.62 | |  |
| rs17248776 | 4.44E-11 | 0.0971 | 0.47 | | 0.25 | | 0.577 | |  |
| rs17248783 | 4.48E-11 | 0.0976 | 0.468 | | 0.25 | | 0.576 | |  |
| rs2228671 | 5.00E-11 | 0.104 | 0.461 | | 0.26 | | 0.583 | |  |
| rs118147862 | 4.09E-10 | 0.775 | 0.59 | | 0.237 | | 0.876 | |  |
| rs41290120 | 1.37E-10 | 0.862 | 0.688 | | 0.398 | | 0.796 | |  |
| rs6857 | 6.83E-08 | 0.0913 | 0.0951 | | 0.0177 | | 0.000122 | |  |
| rs769449 | 6.13E-08 | 0.0759 | 0.122 | | 0.0367 | | 0.129 | |  |
| rs429358 | 2.17E-09 | 0.00967 | 0.232 | | 0.00412 | | 0.00316 | |  |
| rs7412 | 8.17E-11 | 0.419 | 0.304 | | 0.159 | | 0.947 | |  |
| rs10414043 | 2.23E-08 | 0.124 | 0.104 | | 0.0182 | | 0.0366 | |  |
| rs7256200 | 1.78E-08 | 0.11 | 0.109 | | 0.0202 | | 0.0408 | |  |
| rs12721046 | 1.70E-09 | 0.226 | 0.466 | | 0.000325 | | 0.0383 | |  |
| rs12721051 | 1.98E-10 | 0.0841 | 0.709 | | 6.05E-06 | | 0.0015 | |  |
| rs56131196 | 8.24E-11 | 0.0833 | 0.687 | | 7.13E-06 | | 0.00505 | |  |
| rs4420638 | 7.07E-11 | 0.0966 | 0.681 | | 7.40E-06 | | 0.00483 | |  |
| rs111789331 | 1.18E-08 | 0.174 | 0.568 | | 0.000438 | | 0.0553 | |  |
| rs66626994 | 1.42E-08 | 0.188 | 0.639 | | 0.000652 | | 0.0605 | |  |
| rs9979847 | 1.81E-09 | 0.32 | 0.452 | | 0.883 | | 0.985 | |  |
| rs9980151 | 2.84E-09 | 0.282 | 0.435 | | 0.931 | | 0.84 | |  |
| rs28451064 | 1.33E-15 | 0.153 | 0.00994 | | 0.627 | | 0.189 | |  |
| rs9305545 | 5.63E-14 | 0.272 | 0.0719 | | 0.677 | | 0.496 | |  |
| rs9976596 | 1.21E-13 | 0.377 | 0.0692 | | 0.701 | | 0.53 | |  |
| rs9982601 | 1.33E-13 | 0.243 | 0.0243 | | 0.738 | | 0.168 | |  |
| rs9980618 | 1.77E-13 | 0.251 | 0.0216 | | 0.748 | | 0.196 | |  |
| rs60687229 | 1.47E-11 | 0.451 | 0.0385 | | 0.532 | | 0.161 | |  |
| rs9977419 | 1.21E-13 | 0.409 | 0.0685 | | 0.877 | | 0.138 | |  |
| rs9977093 | 1.32E-13 | 0.41 | 0.0687 | | 0.883 | | 0.138 | |  |
| rs8131284 | 5.26E-14 | 0.601 | 0.104 | | 0.705 | | 0.0486 | |  |
| rs146711790 | 1.08E-12 | 0.733 | 0.774 | | 0.976 | | 0.163 | |  |
| rs7279799 | 1.18E-13 | 0.658 | 0.0866 | | 0.764 | | 0.0564 | |  |
| rs9978407 | 1.57E-13 | 0.674 | 0.0722 | | 0.789 | | 0.059 | |  |
| rs7281592 | 2.30E-11 | 0.941 | 0.106 | | 0.955 | | 0.0376 | |  |
| rs7278204 | 2.46E-13 | 0.708 | 0.0706 | | 0.778 | | 0.0585 | |  |
| rs8131303 | 3.51E-13 | 0.701 | 0.0761 | | 0.78 | | 0.0559 | |  |
| rs28593428 | 4.79E-13 | 0.713 | 0.0944 | | 0.755 | | 0.0516 | |  |
| rs73203255 | 3.01E-13 | 0.676 | 0.103 | | 0.778 | | 0.0579 | |  |
| rs973754 | 4.86E-13 | 0.769 | 0.262 | | 0.55 | | 0.0819 | |  |
| rs7280612 | 4.96E-13 | 0.691 | 0.106 | | 0.792 | | 0.0531 | |  |
| rs7278845 | 4.60E-13 | 0.692 | 0.105 | | 0.792 | | 0.0557 | |  |
| rs7283231 | 4.09E-11 | 0.93 | 0.103 | | 0.969 | | 0.0467 | |  |
| rs9983490 | 2.64E-13 | 0.713 | 0.0785 | | 0.753 | | 0.0534 | |  |
| rs2834430 | 8.12E-11 | 0.876 | 0.11 | | 0.984 | | 0.0598 | |  |
| rs9974878 | 2.69E-11 | 0.952 | 0.116 | | 0.892 | | 0.0364 | |  |
| rs28591415 | 3.58E-13 | 0.68 | 0.105 | | 0.843 | | 0.0425 | |  |
| rs2834431 | 7.62E-11 | 0.845 | 0.11 | | 0.96 | | 0.0392 | |  |
| rs16991453 | 3.16E-11 | 0.994 | 0.137 | | 0.973 | | 0.0434 | |  |
| rs2834435 | 7.83E-11 | 0.9 | 0.135 | | 0.964 | | 0.0553 | |  |
| rs8132042 | 4.26E-13 | 0.768 | 0.0847 | | 0.64 | | 0.0478 | |  |
| rs9978142 | 3.32E-11 | 0.959 | 0.104 | | 0.953 | | 0.0441 | |  |
| rs10470171 | 1.06E-12 | 0.655 | 0.0656 | | 0.623 | | 0.0612 | |  |
| rs762158 | 9.98E-12 | 0.191 | 0.0169 | | 0.894 | | 0.0613 | |  |
| rs743339 | 2.23E-11 | 0.244 | 0.0237 | | 0.818 | | 0.154 | |  |
| rs7277800 | 8.95E-11 | 0.254 | 0.0239 | | 0.918 | | 0.174 | |  |
| rs9982672 | 2.38E-10 | 0.404 | 0.00748 | | 0.993 | | 0.158 | |  |
| rs7280276 | 3.31E-10 | 0.348 | 0.0073 | | 0.955 | | 0.15 | |  |
| rs2096467 | 4.02E-08 | 0.362 | 0.00705 | | 0.99 | | 0.148 | |  |
| rs2211695 | 1.17E-08 | 0.366 | 0.00498 | | 0.97 | | 0.131 | |  |
| rs2211693 | 1.44E-08 | 0.361 | 0.00492 | | 0.956 | | 0.135 | |  |
| rs4817636 | 1.35E-08 | 0.348 | 0.00612 | | 0.899 | | 0.138 | |  |
| rs2211694 | 9.38E-09 | 0.337 | 0.00557 | | 0.934 | | 0.135 | |  |
| rs7279974 | 1.08E-08 | 0.335 | 0.00574 | | 0.903 | | 0.145 | |  |
| rs8129119 | 9.61E-09 | 0.33 | 0.00606 | | 0.897 | | 0.145 | |  |
| rs4817637 | 1.23E-08 | 0.306 | 0.00645 | | 0.916 | | 0.142 | |  |
| rs1018757 | 1.39E-09 | 0.17 | 0.00377 | | 0.837 | | 0.197 | |  |
| rs2834439 | 1.11E-08 | 0.296 | 0.00705 | | 0.971 | | 0.173 | |  |
| rs2096469 | 8.73E-09 | 0.278 | 0.00627 | | 0.905 | | 0.144 | |  |
| rs8128536 | 1.27E-08 | 0.283 | 0.00571 | | 0.928 | | 0.132 | |  |
| rs1023354 | 1.30E-08 | 0.261 | 0.00752 | | 0.878 | | 0.226 | |  |
| rs4817639 | 5.39E-08 | 0.117 | 0.0248 | | 0.624 | | 0.412 | |  |
| rs79158929 | 3.64E-10 | 0.524 | 0.17 | | 0.495 | | 0.663 | |  |
| rs5760293 | 1.65E-09 | 0.874 | 0.181 | | 0.06 | | 0.991 | |  |
| rs5760302 | 2.56E-09 | 0.905 | 0.195 | | 0.0504 | | 0.959 | |  |
| rs62233066 | 2.40E-09 | 0.94 | 0.207 | | 0.0566 | | 0.948 | |  |
| rs28361287 | 2.41E-09 | 0.888 | 0.204 | | 0.053 | | 0.934 | |  |
| rs5751836 | 4.31E-09 | 0.883 | 0.211 | | 0.0516 | | 0.937 | |  |
| rs57636940 | 2.53E-09 | 0.873 | 0.254 | | 0.057 | | 0.921 | |  |
| rs62233084 | 6.96E-09 | 0.931 | 0.253 | | 0.0558 | | 0.868 | |  |
| rs62233085 | 6.96E-09 | 0.927 | 0.255 | | 0.0568 | | 0.868 | |  |
| rs5751841 | 1.17E-08 | 0.912 | 0.26 | | 0.0417 | | 0.89 | |  |
| rs9624456 | 8.48E-09 | 0.888 | 0.266 | | 0.0509 | | 0.911 | |  |
| rs2298379 | 2.46E-08 | 0.913 | 0.283 | | 0.0483 | | 0.917 | |  |
| rs5760347 | 1.59E-08 | 0.894 | 0.566 | | 0.0133 | | 0.938 | |  |
| rs5760350 | 1.65E-08 | 0.897 | 0.262 | | 0.0458 | | 0.917 | |  |
| rs62233133 | 2.55E-08 | 0.877 | 0.243 | | 0.0437 | | 0.921 | |  |
| rs62233136 | 4.28E-08 | 0.871 | 0.196 | | 0.0338 | | 0.969 | |  |
| rs5760359 | 4.47E-08 | 0.859 | 0.268 | | 0.0408 | | 0.946 | |  |
| rs5760368 | 2.48E-08 | 0.861 | 0.247 | | 0.0423 | | 0.949 | |  |
